# Calibrating Catalytic DNA Nanostructures for Site‐Selective Protein Modification**

**DOI:** 10.1002/chem.202200895

**Published:** 2022-07-25

**Authors:** Jordi F. Keijzer, Han Zuilhof, Bauke Albada

**Affiliations:** ^1^ Laboratory of Organic Chemistry Wageningen University & Research Stippeneng 4 6708 WE Wageningen The Netherlands; ^2^ School of Pharmaceutical Science & Technology Tianjin University 92 Weijin Road, Nankai District Tianjin 300072 P. R. China; ^3^ Department of Chemical and Materials Engineering Faculty of Engineering King Abdulaziz University 21589 Jeddah Saudi Arabia

**Keywords:** acylation catalyst, bioconjugation chemistry, hGQ DNAzyme, protein-DNA conjugate, site-selective modification

## Abstract

Many biomedical fields rely on proteins that are selectively modified. These can be attached using reactive or catalytic moieties, but the position where these moieties are attached is often poorly controlled. We assessed how catalyst position affects the efficiency and selectivity of protein modification. For this, we anchored a template DNA strand to three different proteins, which were subsequently hybridized to DNA strands that contained catalysts at different positions. We found a strong correlation between the catalyst‐to‐protein distance and the efficiency of protein modification for acyl transfer catalysts, which operate via a covalently bound reactant intermediate. Additionally, we found that the catalyst's distance and orientation with respect to the protein surface, also influences its site‐selectivity. A catalyst operating with unbound reactant intermediates showed only enhanced efficiency. Our results are rationalized using computational simulations, showing that one‐point anchoring of the DNA construct leads to notable differences in the site of modification.

## Graphical abstract

**Three protein‐DNA conjugates** were designed to calibrate the protein modification abilities of catalyst‐functionalized DNA. For two catalysts, a clear correlation between distance and efficiency was found and both linker between DNA and protein as well as position and type of catalyst influenced the site of modification. Computational simulations were used to corroborate the findings.

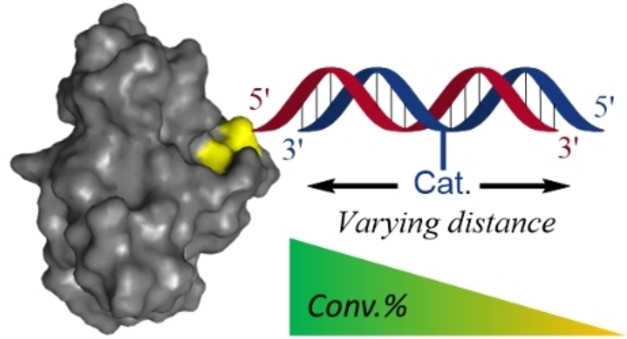

## Introduction

Modified proteins are important to a large variety of scientific and commercial applications, including biomaterials,^[1]^ therapeutics^[2]^ and proteomics.^[3]^ Generally applicable approaches for artificial chemical modification of proteins are thus of great importance. But despite recent progress,[^4^, ^5^, ^6^, ^7^] widely applicable methods for site‐specific protein modification remain elusive, because the outcome of many strategies changes when they are applied in alternate settings or on different proteins with other micro‐environments. Methods have been established to derivatize specific residues in some proteins, for example, via genetic incorporation of orthogonal groups, such as azides or alkynes,[^5^, ^6^] recombinant proteins with unique micro‐environments,^[8]^ or by optimized reagents designed to target single residues.[^9^, ^10^, ^11^] Even the total synthesis of small proteins is a possibility.^[12]^ However, none of these have been proven to be generally applicable to a variety of wild‐type proteins, and most depend for their specificity on specific details of the protein involved.

As alternative to the approaches mentioned above, catalytic protein modification applies a molecular unit that activates an inert moiety, which in turn reacts with amino acid residues on the protein surface.[^5^, ^13^] Such protein modification catalysts can be organometallic,[^14^, ^15^] organic,[^16^, ^17^, ^18^] enzymatic^[19]^ or even based on DNA.[^20^, ^21^] In order to achieve site selectivity in the modification, catalysts rely on a protein‐binding element that brings them to a specific site of the target protein.[^5^, ^13^] These elements can bind proteins covalently, such as linchpins,[^18^, ^22^] or non‐covalently, such as ligands[^15^, ^16^, ^17^] and DNA aptamers.^[23]^ Although effective, the issue that arises in this strategy, is finding the ideal position of the catalyst with respect to the interface between protein and protein‐binding moiety.[^23^, ^24^, ^25^]

Previous work from our group showed that the position of acyl transfer catalysts on a protein‐binding aptamer with respect to the protein, affected the conversion to modified protein and site‐selectivity of the modification.^[23]^ However, due to the dynamic interaction between aptamer and protein, we were not able to gain sufficient insight in the interplay between dimensions of the nanostructure and positioning of the catalyst and its effect on protein modification.

Therefore, we covalently linked catalytic DNA constructs to a protein in order to extract design principles that would allow us to develop next‐generation protein modification tools. Specifically, we covalently conjugated the 5’ end of a template DNA strand (DNA_temp_) to a protein and used it to hybridize its complementary strand that carries a protein‐modifying catalyst at various distances from the 3’ end (DNA_catalyst_). For this, we synthesized protein‐DNA_temp_ constructs using three proteins of various size and analyzed the effective catalyst distance of three different catalysts: the acylation catalysts dimethylaminopyridine (DMAP) and pyridinecarbaldehyde oxime (PyOx), and the oxidative cross‐coupling catalyst hemin/G‐Quadruplex (Figure S3, Supporting Information). Whereas the first two catalysts acylate nucleophilic residues such as Lys and Ser by means of a catalyst‐bound reactive intermediate,^[23]^ the latter catalyst generates a soluble reactive species that reacts with the electron‐rich aromatic rings of Tyr and Trp.^[20]^

## Results and Discussion

Glutaredoxin 1 (GRX) is a small 9.5 kDa protein that contains a single disulfide‐bridge as its active site (Figure S1, Supporting Information). Using a slight excess (2 equiv.) of dithiolthreitol (DTT), the bridge was reduced and 1‐azidomethyl‐3,5‐bis(bromomethyl)benzene could be conjugated to the active site by reacting with both Cys residues, resulting in the installation of a single azide group on GRX. After purification by spin‐filtration, mass spectrometry confirmed the identity of the product and that no crosslinking of GRX had occurred. Then, DNA_temp_ was attached to the azide using CuAAC, and the formed GRX‐DNA_temp_ conjugate could be purified using ion‐exchange FPLC. The identity and purity of the GRX‐DNA_temp_ conjugate was confirmed with HPLC‐MS and SDS‐PAGE. The complementary DNA‐catalyst strand (that is, DNA_diDMAP_ or DNA_diPyOx_) was prepared by CuAAC ligation of azido‐diDMAP or azido‐diPyOx to different complementary DNA strands. These strands contained one octynyl‐modified thymine at different positions in the oligomer, namely at position 1, 2, 3, 4, 6 or 8 with respect to the 3’ end of the oligomer (Scheme S4, Supporting Information). After purification by spin filtration, HPLC‐MS confirmed that the correct constructs were formed and obtained with >95 % purity.

**Figure 1:**
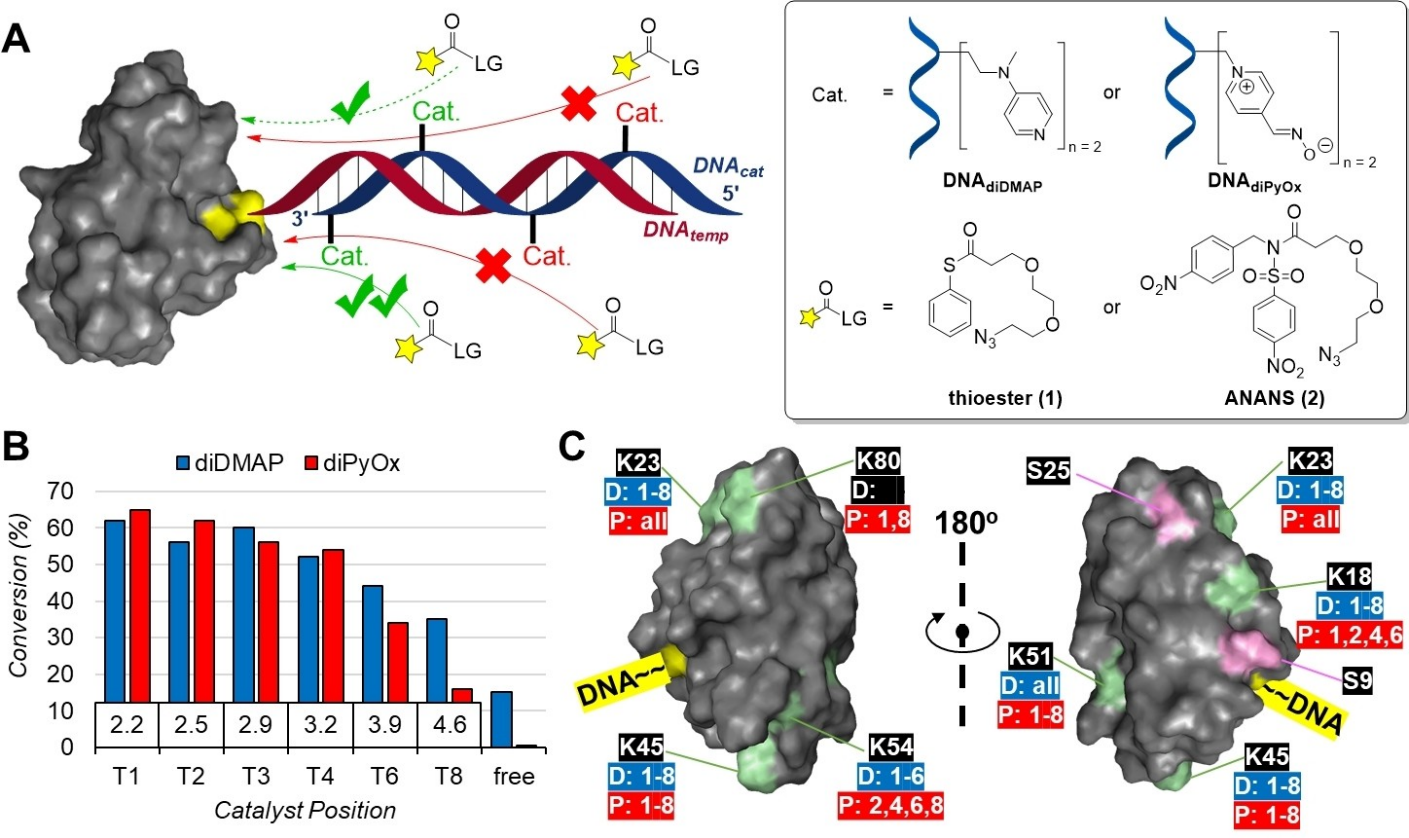
A) Structures of acyl donors **1** and **2** for diDMAP and diPyOx, respectively; B) Diagram showing the decline in conversion percentages of GRX‐DNA_temp_ by diDMAP or diPyOx when positioned further away from the protein surface with T1 being the closest. The numbers in the boxes show the distance between nucleobase and protein surface in nm. C) Crystal structure(s) of GRX (PDB code: 1EGO) showing Lys residues (green) with the numbers of catalyst positions that modify them (D=diDMAP (blue), P=diPyOx (red), all=including free catalyst) as well as the attachment site on the DNA strand. Conditions: 20 μM GRX‐DNA_temp_ with (a) 22 μM DNA_diDMAP_ and 100 μM thioester **1**, pH: 8.0, at 37 °C for 2 h or (b) 22 μM DNA_diPyOx_ and 300 μM ANANS **2**, pH: 7.2, at 37 °C for 6 h.

After the different components required for the protein modification studies were obtained, we assessed how the efficiency of modification was affected by the details of the protein‐catalyst interaction. For this, we first incubated the DNA_diDMAP_ strands in a 1 : 1 ratio with GRX‐DNA_temp_ in HEPES buffer (pH: 8.0) for 30 min. Then, thioester **1** (Figure 1A) was added and after 2 h the reaction was quenched by addition of an excess ethanolamine. Depending on the success of the DNA‐bound catalyst we thus obtain none, one or more N_3_‐terminated chains on the GRX surface. Subsequently, the acylated protein was clicked using SPAAC with a bicyclononyne (BCN)‐functionalized 2 kDa PEG unit, which causes a different band shift for modified bands on SDS‐PAGE. This allowed the separation of modified and unmodified protein and subsequent integration and quantification of the protein bands with ImageJ. We found that positioning of the diDMAP more to the end of the 5’ DNA strand and thus further away from the protein surface did not markedly vary with positioning in the first three sites but resulted in a gradual decrease in acylation of GRX‐DNA_temp_ when the catalyst was moved further out (Figure 1B). This demonstrates a clear correlation between catalyst‐to‐protein distance and modification efficiency.

We also incubated GRX‐DNA_temp_ with various DNA‐diPyOx strands in HEPES buffer (pH: 7.2) for 30 min, after which its dedicated substrate alkyl‐*N*‐acyl‐*N*‐sulfonamide (ANANS) **2** (Figure 1A) was added. In this case, the reaction was quenched by the addition of citrate buffer (pH: 5.0) and worked up as described above for the diDMAP‐catalyzed modifications. For diPyOx, we found a similar decrease in acylation by distancing the catalyst from the protein surface and again T1 being the most efficient position (Figure 1B). Surprisingly, the total conversion performed by the diDMAP catalyst far exceeded that of the diPyOx system, as the former generated similar conversions using only a third of the concentrations of acyl donor (Figure 1B).

This finding is opposed to what was observed in aptamer‐based experiments, in which diPyOx generated higher yields.^[23]^

After this, we analyzed the site selectivity of the modification by tryptic digestion and follow‐up MS/MS for both of these catalysts. GRX contains a total of six Lys residues: K18 (SAA: 269 Å^2^), K23 (SAA: 276 Å^2^), K45 (SAA: 280 Å^2^), K51 (SAA: 275 Å^2^), K54 (SAA: 299 Å^2^), K80 (SAA: 274 Å^2^); and two Ser residues: S9 (SAA: 207 Å^2^) and S25 (SAA: 206 Å^2^). All positions of diDMAP (T1‐T8) acylate five of the six Lys residues, the most remotely positioned residue K80 remained unmodified (Figure 1C). Similarly, diDMAP on T8 also cannot reach K54, which is likely again caused by distance. Interestingly, diPyOx resulted in a more varied modification landscape: K80 was modified when diPyOx was positioned on T1 and T8, but not on the intermediate positions T2–T6. Similarly, K18 remained unmodified by diPyOx on T3 and T8, and K54 was unmodified by diPyOx on T1 and T3. We hypothesize that this results not only from increasing distances, but that also the helical shape of the dsDNA plays a role, which yields with this variation in distance also a distance in spatial orientation. Apparently, this is not critical from diDMAP, but is critical for diPyOx, suggesting that subtle differences in catalyst and linker between the two systems also contribute to preferences in site‐selectivity of the performed modification.

**Figure 2:**
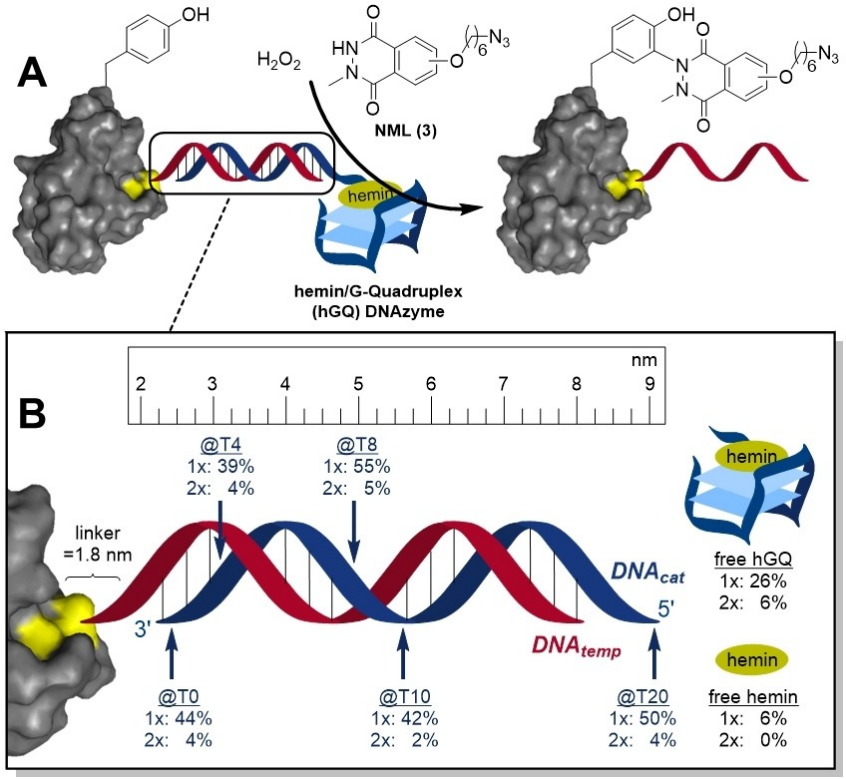
A) The G‐Quadruplex‐forming sequence PW17 is included in the hybridizing strand and by addition of hemin, a protein‐bound hGQ DNAzyme is formed. When H_2_O_2_ is present, the DNAzyme conjugates *N*‐methyl‐luminol **3** (NML) to tyrosine residues on the protein, which can be visualized after removal of the hGQ‐containing DNA strand. B) The different positions where PW17 was included with the percentages of single and double modification that the DNAzyme generated. Conditions: 20 μM GRX‐DNA_temp_, 22 μM DNA‐hGQ, 30 μM NML **3** and 100 μM H_2_O_2_, pH: 7.0, at 25 °C for 30 min.

We also assessed if a similar level of control over the selectivity of the modification was available by a hemin/G‐quadruplex (hGQ) catalyst. As the hGQ DNAzyme generates a soluble unbound radical reactant that has to diffuse to a proximal reactive site, we anticipated to see an effect of the distance on the modification efficiency (Figure 2A). Therefore, the hybridizing DNA strand was designed to include a G‐Quadruplex folding sequence, i. e., PW17, so that upon hybridization with GRX‐DNA_temp_ and addition of hemin, a protein‐bound hGQ DNAzyme was formed. As the PW17 DNAzyme forms a hybrid GQ structure with the 3’‐ and 5’‐end positioned in close proximity, we anticipated that a functional hGQ DNAzyme would be formed even when placing the GQ sequence non‐terminally.^[26]^ By positioning the PW17 sequence at different sites on the dsDNA unit, we would be able to assess the effect of distance on the modification. We performed the conjugation using azide‐linked *N*‐methyl‐luminol (NML) **3** and H_2_O_2_, followed by quenching with catalase and a subsequent SPAAC reaction between the azide‐functionalized protein and BCN‐PEG_2000_. The PEGylated proteins were then separated using SDS‐PAGE and the Coomassie‐stained bands of the modified proteins were integrated. Even though bound hGQ resulted in higher conversions than unbound hGQ, different positions of the catalyst on the DNA oligomer did not generate much variation, not even when the catalyst was positioned at T20, which is ∼9 nm away from the protein surface (Figure 2B).^[27]^ Apparently, the produced NML radical survives sufficiently long to diffuse to over at least 9 nm to reactive sites on the protein. In view of the similarities of the level of modification at different positions, the site‐selectivity of the modification was not further examined. Clearly, use of a catalyst that generates a soluble reactive species requires additional levels of control when compared to an approach that relies on an activated catalyst‐bound intermediate.

After these encouraging results for the modification of the small protein GRX using DMAP and PyOx catalysts, we applied larger proteins in order to determine how the distance between catalyst and protein would affect the region of acylation. We thus devised a way to synthesize one or more protein‐DNA conjugates, large enough to observe a possible difference between sites of modification. Paraoxon is a selective inhibitor for serine proteases and binds the serine residue in their active site.^[28]^ Indeed, incubation of serine proteases chymotrypsin (25 kDa) and human α‐thrombin (36 kDa) with paraoxon generated nearly quantitative single modification. We thus synthesized an azido‐functionalized paraoxon **4**, which could easily be made from commercially available tris(*p*‐nitrophenol)phosphate in a single reaction with azido‐ethanol (Scheme S3, Supporting Information).

**Figure 3:**
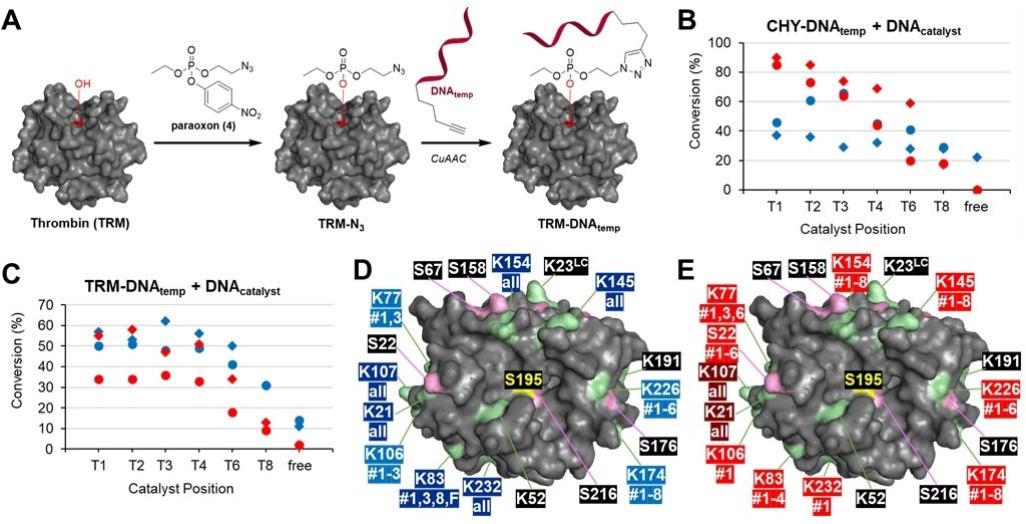
A) Synthesis of thrombin‐DNA (TRM‐DNA_temp_) and chymotrypsin‐DNA (CHY‐DNA_temp_) by using paraoxon derivative **1** (or **2**); B and C) Graphs showing the decline in conversion percentages of B) CHY‐DNA_temp_ and C) TRM‐DNA_temp_ by DNA_diDMAP_ or DNA_diPyOx_ when positioned further away from the protein surface (T1 is 3’ end). The shapes indicate ⧫ for ethyl and • for EG_2_ linkers, where the colors indicate blue for diDMAP and red for diPyOx. Conversions in B) are normalized values. Conditions: 20–26 μM protein‐DNA_temp_ with (i) 23–28 μM PMET‐diDMAP and 100 μM thioester **1**, pH: 8.0, at 37 °C for 2 h or (ii) 23–28 μM DNA‐diPyOx and 300 μM ANANS **2**, pH: 7.2, at 37 °C for 6 h; D and E) Crystal structure of thrombin showing its Lys (green) and Ser (pink) residues with respect to the active site where DNA_temp_ is attached (S195) (PDB‐code: 5EW1^[32]^). LC=Light Chain. D) Modification sites by diDMAP. E) Modification sites by diPyOx. Light colored numbers indicate residues modified with only bound catalysts and dark colored numbers indicate residues also modified with unbound catalyst.

Incubation of chymotrypsin (CHY) and thrombin (TRM) with this azido‐paraoxon (Figure 3A) and subsequent purification by spin filtration, resulted in quantitative formation of singly modified proteases as determined by LC‐MS. DNA_temp_ was then attached by means of CuAAC, generating yields of 67 % and 50 % for chymotrypsin (CHY‐Et‐DNA_temp_) and thrombin (TRM‐Et‐DNA_temp_), respectively. As we anticipated that the potentially hidden azide in this first approach might hamper attachment of the DNA anchor strand, we also synthesized azido‐EG_2_‐paraoxon **5** (Scheme S3, Supporting Information) to make the protein‐bound azide moiety more accessible.

Indeed, conjugation yields of the DNA to the proteins increased to 72 % for chymotrypsin (CHY‐EG_2_‐DNA_temp_) and 65 % for thrombin (TRM‐EG_2_‐DNA_temp_), respectively. These two different tethers enabled us to correlate their effect on the modification performance of the different DNA‐based catalysts.

Purification of these protein‐DNA_temp_ conjugates was again performed by ion‐exchange FPLC. Unfortunately, whereas product formation was confirmed with SDS‐PAGE, the products suffered from degradation during or after the ion‐exchange FPLC purification. By carefully monitoring the product in each synthetic step, we discovered that our protein‐DNA_temp_ conjugates degraded in the absence of glycerol. As such, we choose to purify these larger protein‐DNA_temp_ conjugates only by spin filtration using a molecular weight cut off value of 10 kDa. Fortunately, this was sufficient to remove the bulk of the remaining DNA_temp_ (∼6 kDa), while it still allowed the use of glycerol in the solutions to retain the stability of the constructs.

After obtaining our protein‐DNA_temp_ constructs, we hybridized the corresponding DNA_diDMAP_ or DNA_diPyOx_ strands and performed our protein acylation studies. When applying diDMAP and diPyOx for the acylation of CHY‐DNA_temp_, the results were difficult to interpret due to protein degradation during the modification steps, thus only normalized values for the conversion could eventually be obtained (Figure 3B). Thrombin‐DNA, however, did not degrade during the reaction and after PEGylation in the second step, we could derive product concentrations from the integrated intensities of SDS‐PAGE gels with ImageJ (Figure 3C). For both diDMAP and diPyOx we found the same inverse correlation between acylation percentage and catalyst distance as we observed for GRX‐DNA_temp_ (Figure 1B), and that the best conversions are obtained when the catalyst is connected at the first few nucleotides of the protein‐bound dsDNA unit. Unbound DNA‐diDMAP or DNA‐diPyOx strands led to marginal protein modification, that is, <3 % and <6 %, respectively (Figures S13 and S20, Supporting Information). Remarkably, for both PEG‐linked conjugates, the ideal position of diDMAP seems to be T3. This does, however, not seem the case for the ethyl‐linked conjugates where T4 appears to be slightly higher. It appears that diDMAP is optimally positioned at roughly 4 nucleobases distance when the dsDNA is connected to the protein by means of an ethyl unit, whereas for the slightly longer EG_2_ linker the optimal position is at nucleotide T3. Notably, tests of TRM‐DNA_temp_ with the hGQ DNAzyme revealed little to no variation in modification percentage (Figures S18 and S19, Supporting Information), which confirms our finding for GRX‐DNA_temp_ that this protein‐modifying catalyst does, within this distance range, not benefit from a shorter distance to the protein.

Analysis of the site‐selectivity of the modifications by these dsDNA‐bound catalysts was performed by tryptic digestion and follow‐up MS/MS. To our delight, we found that both catalysts primarily acylated residues in close proximity to the active site where the catalytic dsDNA construct was anchored (Figure 3D and E). In fact, distant residues are only modified when the catalyst is positioned close enough to the anchor point of the dsDNA. As such, K226 is modified by diDMAP at a distance of <8 nucleobases and K106 only at <4 nucleobases. Similarly, K226 and S22 are modified by diPyOx at <8 nucleobases, K83 only at <6 nucleobases and K106 and K232 only at 1 nucleobase distance. This implies that by positioning the catalyst further away, a trade‐off between conversion and selectivity can be made where conversion could be reduced to attain a higher level of site‐selectivity, and *vice versa*. Interestingly, although diPyOx can also acylate Ser residues (in contrast to diDMAP), of all nearby residues only Ser22 was modified. As the solvent‐accessible area (SAA) of Ser22 is in the same range as that of the other serine residues (Ser22=206 Å^2^, Ser67=206 Å^2^, Ser158=203 Å^2^, Ser176=205 Å^2^ and Ser216=201 Å^2^), distance rather than accessibility is important, so that this is likely the only serine residue within reach. Once more, however, the spatial orientation caused by the helicity of the dsDNA has to be considered as well, as modification of some residues (K77 and K83 for diDMAP and K77 for diPyOx) does not directly correlate to distance.

**Figure 4:**
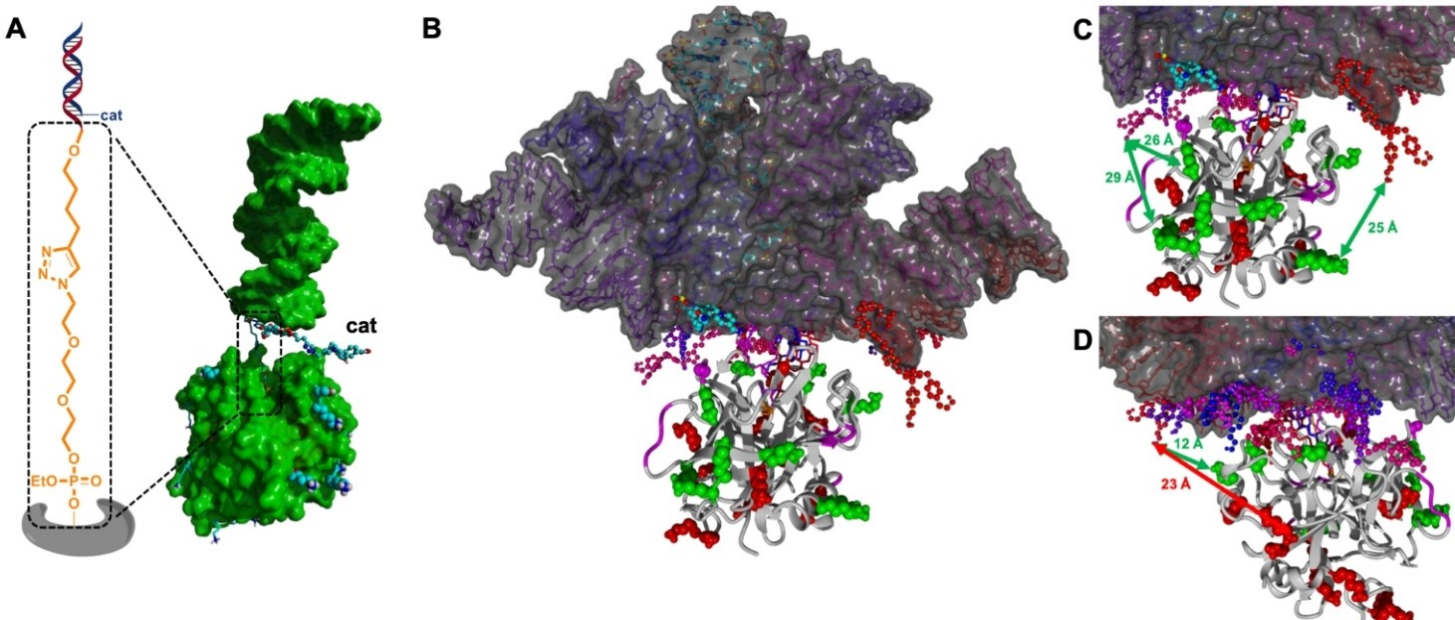
Molecular model of the thrombin‐dsDNA‐diPyOx construct to rationalize the observed modifications. A) Details of the model, with emphasis on the linker between protein and dsDNA; the linker is shown in sticks, the diPyOx catalyst in ball‐and‐stick, the modified Lys residues and one Ser residue in balls, and the unmodified Lys residues in sticks; surfaces of the protein and DNA are depicted in green, except for the previously described details. In the model, the dsDNA unit is shown on top and the protein at the bottom. B) Resulting 10 structures of sampling the dihedral angles of the spacer between protein and dsDNA, and of diPyOx‐functionalized T1. Modified Lys residues are shown as green balls, modified Ser residue as magenta balls, unmodified Lys residues as red balls. The position of the protein is fixed, the 10 differently positioned dsDNA‐diPyOx units are coloured from blue to red, their surfaces are shown in grey. C and D) Zoomed parts of the interface between dsDNA‐diPyOx and thrombin.

Finally, in order to obtain a better understanding of how the positioning of the catalyst on the dsDNA affects the site of modification, we performed computational simulations using thrombin as our protein of interest. First, we constructed the dsDNA‐thrombin conjugate using the single crystal X‐ray structure of the protein (PDB‐code 5EW1),^[29]^ and a model of the appropriate dsDNA equipped with a diPyOx catalyst on position T1. As the difference in residues that were modified by ethyl and EG_2_‐linked dsDNA constructs was minimal, we focused this analysis on the EG_2_‐linked constructs (Figure 4A). We sampled the conformational space of the construct using internal coordinates of the model, with focus on the linker between Ser195 and dsDNA and on the diPyOx‐functionalized T1. Furthermore, dihedral angles were set to be limited to energetically favor the staggered conformation, and bumps between structures were minimized. As a result, an umbrella of 10 different structures was generated (Figure 4B–D), revealing an impression of the reach of the catalyst when attached at T1. Closer inspection of the positions of the catalysts confirms that all the modified residues lie within reach of the catalyst, and that unmodified residues are either outside of the range that can interact with the catalyst, or are via its spatial orientation hidden from the catalyst by the dsDNA unit, which is the case for K52 that is next to the active site (Figure 3D and E). As the distances between some of the more remotely positioned residues are large, this analysis reveals the presence of considerable freedom in this system. This could be expected based on the degrees of freedom in the linkers, which translates to the rotational freedom of the dsDNA part. Therefore, more control over the precision of the modification can likely be obtained by additional anchoring of the catalytic DNA constructs.

## Conclusion

The catalytic activity and site‐selectivity of DNA‐bound protein‐modifying moieties was tested as a function of distance of the catalyst to the reactive site at three proteins, including two serine proteases. Two specific relations were found: i) If the catalyst has to reach at the site of the protein to perform its action, then a strong tapering down of activity was observed with increasing distance. This situation takes place, for example, with DMAP and PyOx modification of Lys and Ser residues on the protein surface. Basically, no distance dependence was observed for a distance of 1–2 base pairs, after which a gradual decline to far lower reactivity was observed for catalysts up to 8 base pairs distance from the reactive site. ii) If, in contrast, the catalyst produces a reactive, soluble intermediate that itself reacts at the protein surface, then there is basically no distance dependence if the intermediate is sufficiently long‐lived to allow diffusion‐mediated reactivity. This situation occurs with the hGQ DNAzyme –which uses H_2_O_2_ to produce a long‐lived, yet sufficiently reactive intermediate radical– then no decline in reactivity is observed even with up to 20 nucleobases distance. Finally, we noticed that in specific cases, the effect of even one additional nucleobase could be quite significant, which we attribute to a combination of distance and spatial orientation. Following these conclusions, we anticipate that these results will help the design of future probes with either of the two types of catalysts (activation via a bound reactant intermediate or an unbound radical reactant) investigated in this work by taking into account the described effects of distance and spatial orientation. We also note that our work adds another strategy to create single DNA‐protein conjugates, which are in high demand for applications in the fields of diagnostics, medicine and nanotechnology. More specifically, for bioassays, caged‐enzymes or carriers for enzymes or nucleic acids for gene editing and disease treatment.[^29^, ^30^, ^31^]

## Experimental Section

**Synthesis of GRX‐DNA_temp_**: Glutaredoxin 1 was incubated with DTT (1.5 equiv.) in 15 mM NH_4_HCO_3_ (pH: 8.0) at 37 °C for 30 min, followed by incubation with 1‐(azidomethyl)‐3,5‐bis(bromomethyl)benzene (2.5 Equation) at 37 °C for 2.5 h and subsequently purified by spin filtration over 3 kDa MWCO Amicon® Ultra‐15 Centrifugal Filter Units, washing 3 times with 50 mM NaCl solution in 25 mM Tris (pH: 8.0). The synthesized azido‐protein was treated with 2 equiv. of DNA_temp_ (from 500 μM stock in oxygen‐poor ddH_2_O) with respect to the protein concentration, 100 μM [Cu ⋅ THPTA] (complex of CuSO_4_ and THPTA mixed in a ratio of 1 : 5 in ddH_2_O) and 10 mM sodium ascorbate (from a freshly made stock of 100 mM in oxygen‐poor ddH_2_O) and incubated in the dark at 12 °C for 16–20 h. The formed GRX‐DNA_temp_ construct was purified by FPLC, using an ion‐exchange MonoQ column (Vol: 1 mL) using a gradient from 0–1 M NaCl in 20 mM Tris (pH: 8.0). The collected fractions were and concentrated by spin filtration over 3 kDa MWCO Amicon® Ultra‐15 Centrifugal Filter Units, washing 3 times with 50 mM NaCl solution in 25 mM Tris (pH: 8.0). The concentration of GRX‐DNA_temp_ was quantified from absorption values determined with a Scientific^TM^ Nanodrop 2000, using a 1 : 1 mixture of native GRX with DNA_temp_ as a reference.

**Synthesis of CHY‐DNA_temp_ and TRM‐DNA_temp_**: Thrombin or Chymotrypsin was incubated with azido‐paraoxon (20 equv.) in 20 % glycerol in 50 mM HEPES (pH: 7.2) at 37 °C for 3 h, and subsequently purified by spin filtration over 10 kDa MWCO Amicon® Ultra‐15 Centrifugal Filter Units, washing 3 times with 20 % glycerol in 50 mM NaCl solution in 25 mM Tris (pH: 8.0) for thrombin and 50 mM NaCl solution in 25 mM Tris (pH: 8.0) for chymotrypsin. The respective azido‐protein was treated with 2–3 equiv. of DNA_temp_ (from 500 μM stock in oxygen‐poor ddH_2_O) with respect to the protein concentration, 100–150 μM [Cu ⋅ THPTA] (complex of CuSO_4_ and THPTA mixed in a ratio of 1 : 5 in ddH_2_O) and 7–10 mM sodium ascorbate (from a freshly made stock of 100 mM in oxygen‐poor ddH_2_O) and incubated in the dark at 12 °C for 16–20 h. The synthesized protein‐DNA_temp_ constructs were purified by spin filtration over 10 kDa MWCO Amicon® Ultra‐15 Centrifugal Filter Units, washing 3 times with 20 % glycerol in 50 mM NaCl solution in 25 mM Tris (pH: 8.0) for thrombin and 50 mM NaCl solution in 25 mM Tris (pH: 8.0) for chymotrypsin. The concentrations were quantified from absorption values determined with a Scientific^TM^ Nanodrop 2000, using a 1 : 1 mixture of native protein with DNA_temp_ as a reference.

**Synthesis of DNA_diDMAP_ and DNA_diPyOx_**: DNA‐alkyne sequences with alkyne‐thymine modification were purchased as HPLC‐purified lyophilized powders from Integrated DNA technologies. The powders were dissolved in oxygen‐poor ddH_2_O. The DNA was treated with 10 equiv. of compound azido‐diDMAP or azido‐diPyOx (from 100 mM stock in DMSO) with respect to the DNA concentration, 100 μM [Cu ⋅ THPTA] (complex of CuSO_4_ and THPTA mixed in a ratio of 1 : 5 in ddH_2_O) and 10 mM sodium ascorbate (from a freshly made stock of 100 mM in ddH_2_O) and incubated in the dark at 12 °C for 16–20 h. The synthesized PMET‐catalyst construct was purified by spin filtration over 3 kDa MWCO Amicon® Ultra‐15 Centrifugal Filter Units, washing 3 times with 400 mM NaCl solution in ddH_2_O. Purity and concentration were determined by HPLC‐MS and UV‐Vis.

**Modification of protein‐DNA_temp_ with DNA_diDMAP_**: A mixture was typically prepared containing (a) 20 μM GRX‐DNA_temp_ (from 100 μM stock in 20 mM NH_4_HCO_3_ pH: 8.0) (b) 20–25 μM CHY‐DNA_temp_ (from 150–185 μM stock in 20 mM NH_4_HCO_3_ pH: 8.0) (c) 20–26 μM TRM‐DNA_temp_ (from a 150–200 μM stock solution in 20 % glycerol in 20 mM NH_4_HCO_3_ pH: 8.0), 30 μM DNA_diDMAP_ (from varying stock concentrations in ddH_2_O) in HEPES buffer [50 mM, pH: 8.0, with 350 mM NaCl and 50 mM KCl]. This mixture was incubated in the dark for 20–30 min at 37 °C, after which thioester **1** (from varying stock concentrations in DMSO) was added. The reaction mixture was incubated in the dark at 37 °C for 2 h, shaking the tubes at 500 rpm.

**Modification of protein‐DNA_temp_ with DNA_diPyOx_**: A mixture was typically prepared containing (a) 20 μM GRX‐DNA_temp_ (from 100 μM stock in 20 mM NH_4_HCO_3_ pH: 8.0) (b) 20–25 μM CHY‐DNA_temp_ (from 150–185 μM stock in 20 mM NH_4_HCO_3_ pH: 8.0) (c) 20–26 μM TRM‐DNA_temp_ (from a 150–200 μM stock solution in 20 % glycerol in 20 mM NH_4_HCO_3_ pH: 8.0), 30 μM DNA_diPyOx_ (from varying stock concentrations in ddH_2_O) in HEPES buffer [50 mM, pH: 7.2, with 350 mM NaCl and 50 mM KCl]. This mixture was incubated in the dark for 20–30 min at 37 °C, after which ANANS **2** (from varying stock concentrations in DMSO) was added. The reaction mixture was incubated in the dark at 37 °C for 6 h, shaking the tubes at 500 rpm.

**Modification of protein‐DNA_temp_ with DNA_cat_‐hGQ**: A mixture was typically prepared containing (a) 20 μM GRX‐DNA_temp_ (from 100 μM stock in 20 mM NH_4_HCO_3_ pH: 8.0) (b) 20–25 μM CHY‐DNA_temp_ (from 150–185 μM stock in 20 mM NH_4_HCO_3_ pH: 8.0) (c) 20–26 μM TRM‐DNA_temp_ (from a 150–200 μM stock solution in 20 % glycerol in 20 mM NH_4_HCO_3_ pH: 8.0), 30 μM DNA_cat_‐PW17, 30 μM hemin (from 225 μM stock in DMSO) and 500 μM NML **2** (taken from a 7 mM stock solution in DMSO) in PO_4_ buffer [50 mM, pH=7.0, with 400 mM NaCl and 5 mM KCl]. This mixture was allowed to stand for 20 min after which H_2_O_2_ (from 5 mM stock in in PO_4_ buffer [50 mM, pH=7.0, with 800 mM NaCl and 10 mM KCl]) was added to a final concentration of 500 μM. The reaction mixture was then kept in the dark at 25 °C for 30 min, shaking the tubes at 500 rpm. Afterwards, the reaction was quenched by adding catalase to a final concentration of 0.01 mg/mL (from 0.2 mg/mL stock in (NH_4_)_2_SO_4_ buffer).

**Secondary SPAAC reaction after acylation**: Prior to SDS‐PAGE analysis, additional functionalization is required to visualize the modifications. Two approaches were used: band shifting or fluorescent staining. Band shift assay: modified protein was treated with at least 6 equiv. of BCN‐PEG_2000_ (purchased from Synaffix B.V.) with respect to the concentration of acyl donor and incubated at 12 °C overnight. Fluorescent stain assay: modified protein was treated with 6 equiv. of BCN‐sulphorhodamine B with respect to the acyl donor, 1 and incubated at 12 °C overnight.

**Protocol for SDS‐PAGE analysis**: Acrylamide gels (12 %) were prepared according to Bio‐Rad bulletin 6201 protocol. Specifically, reaction mixtures containing 2–5 μg of protein were diluted with one volume equiv. of SDS‐PAGE sample buffer (2×) containing 10 % BME and incubated for 10 minutes at 95 °C. The denatured sample was then used for SDS‐PAGE analysis (12 % acrylamide gel). Precision Plus Protein™ Dual Color Standards was used as a reference protein ladder. After running, if one of the proteins was modified with a fluorophore, a UV‐photo of the gel was taken. Gels were then stained using Coomassie brilliant blue (0.1 % Coomassie Blue R250 in 10 % acetic acid, 50 % methanol and 40 % demineralized water) by shaking gently for 0.5 h, and destained with destaining solution (10 % acetic acid, 50 % methanol, and 40 % demineralized water) by shaking gently for 1 h. Afterwards, the destaining solution was replaced with H_2_O and shaken gently overnight at room temperature. When the BCN‐PEG_2000_ mass‐tag was used, quantification was performed by integrating the intensity of the Coomassie stained bands of de SDS‐PAGE gel using ImageJ software.

**Tryptic digestion MS/MS analysis of modified protein‐DNA conjugates**: Modified protein samples were subjected to SDS‐PAGE separation and the desired protein bands cut from the gel and cut up to small pieces. The pieces were washed by incubating three times with 50 mM NH_4_HCO_3_ (pH: 8.0) in 50 % ACN in ddH_2_O and subsequently dried in a Speedyvac vacuum centrifuge. The dry pieces were swollen in 50 μL DTT [10 mM in 100 mM NH_4_HCO_3_ (pH: 8.0)] and incubated for 45 min at 56 °C. The supernatant was removed and 50 μL of IAA (55 mM in 100 mM NH_4_HCO_3_ (pH: 8.0)) was added and the pieces were incubated in the dark at rt for 30 min. The supernatant was removed and the pieces were washed by incubating once with 50 mM NH_4_HCO_3_ (pH: 8.0) in 50 % ACN in ddH_2_O and subsequently dried in a vacuum centrifuge. The gel pieces were swollen in 40 μL trypsin gold (125 ng/μL) and incubated at 37 °C for 16–18 h. The initial supernatant was collected and the gel pieces were washed by incubating 15 min at 37 °C with 20 μL NH_4_HCO_3_ (100 mM, pH: 8.0) and 15 min at 37 °C when diluted with 20 μL. The collected supernatants were combined and dried in a vacuum centrifuge and the dry peptide digest dissolved in 20 μL 0.1 % FA. Peptide digests were analyzed on an EASY nanoLC connected to Thermo Scientific^TM^ Q Exactive PLUS. Peptides were trapped onto a PepSep trap column (2 cm×100 μm ID, 5 μm C18 ReproSil) and subsequently separated on a PepSep analytical column (8 cm×75 μm ID, 3 μm C18 ReproSil, PepSep). Elution was achieved using a gradient that started with 5 % (ACN+0.1 % FA) ending with 40 % (ACN+0.1 % FA) in (H_2_O+0.1 % FA), washing the column with 80 % (ACN+0.1 % FA) afterwards. The eluted peaks were then analyzed using MaxQuant software.

## Conflict of interest

The authors declare no conflict of interest.

1

## Supporting information

As a service to our authors and readers, this journal provides supporting information supplied by the authors. Such materials are peer reviewed and may be re‐organized for online delivery, but are not copy‐edited or typeset. Technical support issues arising from supporting information (other than missing files) should be addressed to the authors.

Supporting Information

## Data Availability

The data that support the findings of this study are available in the supplementary material of this article.
