# Supplementary material for: Calibrating Catalytic DNA Nanostructures for Site‐Selective Protein Modification
Source: Chemistry. 2022 Jul 25;28(51):e202200895. doi: 10.1002/chem.202200895 (PMC9546015; doi:10.1002/chem.202200895)
Supplement: Supplementary file 1 — Supporting Information [file CHEM-28-0-s001.pdf]

Chemistry–A European Journal

Supporting Information

Calibrating Catalytic DNA Nanostructures for Site-Selective Protein Modification

Jordi F. Keijzer, Han Zuilhof, and Bauke Albada*

Table of Contents

TABLE OF CONTENTS **2**

LIST OF ABBREVIATIONS **4**

GENERAL INFORMATION **5**

1.1 MATERIALS 5
1.2 DNA SEQUENCES 5

EXPERIMENTAL PROCEDURES **7**

2.1 SYNTHESIS OF ORGANIC COMPOUNDS 7
2.1.1 1,3,5-TRIS(AZIDOMETHYL)BENZENE: 7
2.1.2 ALKYNE-DMAP: 7
2.1.3 AZIDO-DIDMAP: 8
2.1.4 AZIDO-THIOESTER (**1**): 8
2.1.5 1-(AZIDOMETHYL)-3,5-BIS(BROMOMETHYL)BENZENE: 9
2.1.6 AZIDO-DIPYOX: 9
2.1.7 AZIDO-ANANS PRECURSOR: 10
2.1.8 AZIDO-ANANS (**2**): 10
2.1.9 AZIDO-ETHYL-PARAOXON (**4**): 11
2.1.10 AZIDO-EG_2-PARAOXON (**5**): 11

2.2 SYNTHESIS OF PROTEIN-DNA_{TEMP} AND $DNA_{CATALYST}$ **13**

2.2.1 SYNTHESIS OF GRX-DNA_{TEMP} 13
2.2.2 SYNTHESIS OF CHY-DNA_{TEMP} AND TRM-DNA_{TEMP} 14
2.2.3 SYNTHESIS OF DNA_{DMAP} AND DNA_{PYOX} 15

2.3 PROTEIN MODIFICATION STUDIES **16**

2.3.1 PROTOCOL FOR MODIFICATION OF PROTEIN-DNA_{TEMP} WITH DNA_{DMAP} 16
2.3.2 PROTOCOL FOR MODIFICATION OF PROTEIN-DNA_{TEMP} WITH DNA_{PYOX} 16
2.3.3 PROTOCOL FOR MODIFICATION OF PROTEIN-DNA_{TEMP} WITH DNA_{CAT}-HGQ 16
2.3.2 PROTOCOL FOR SDS-PAGE ANALYSIS 17
2.3.3 PROTOCOL FOR THE ANALYSIS OF PROTEIN MODIFICATION ON HPLC(-MS) 17
2.3.4 TRYPTIC DIGESTION MS/MS ANALYSIS OF MODIFIED PROTEIN-DNA CONJUGATES 17

2.4 SDS-PAGE GEL RESULTS **19**

2.4.1 GRX-DNA_{TEMP} WITH DNA_{DIDMAP} & 300 MM THIOESTER 19
2.4.2 GRX-DNA_{TEMP} WITH DNA_{DIDMAP} & 100 MM THIOESTER 19
2.4.3 GRX-DNA_{TEMP} WITH DNA_{DIPYOX} 20
2.4.4 GRX-DNA_{TEMP} WITH DNA_{CAT}-HGQ DNAZYME 20
2.4.5 DEGRADATION OF TRM-DNA_{TEMP} 21
2.4.6 CHY-ET-DNA_{TEMP} WITH DNA_{DIDMAP} 21
2.4.7 CHY-ET-DNA_{TEMP} WITH DNA_{DIPYOX} 22
2.4.8 CHY-EG_2-DNA_{TEMP} WITH DNA_{DIDMAP} 22
2.4.9 CHY-EG_2-DNA_{TEMP} WITH DNA_{DIPYOX} 23
2.4.10 TRM-ET-DNA_{TEMP} WITH DNA_{DIDMAP} 24

2.4.11 TRM-ET-DNA_{TEMP} WITH DNA_{DIPYOX} 24
2.4.12 TRM-EG_2-DNA_{TEMP} WITH DNA_{DIDMAP} 25
2.4.13 TRM-EG_2-DNA_{TEMP} WITH DNA_{DIPYOX} 25
2.4.14 TRM-ET-DNA_{TEMP} WITH DNA_{CAT}-HGQ DNAZYME 26
2.4.15 TRM-EG_2-DNA_{TEMP} WITH DNA_{CAT}-HGQ DNAZYME 26

2.5 HPLC AND MS DATA 28

2.5.1 MS DATA OF CHY-EG_2-N_3 AND TRM-EG_2-N_3 28
2.5.2 HPLC AND MS DATA OF DNA STRANDS 30

2.6 NMR DATA OF ORGANIC COMPOUNDS 37

2.7 HPLC TRACES AND PURITY OF FINAL COMPOUNDS 45

List of Abbreviations

ACN = acetonitrile

ANANS = alkylated *N*-acyl-*N*-sulfonamide

Anh. = anhydrous

BCN = *endo*-bicyclo[6.1.0]nonyne

BME = 2-mercaptoethanol / β-mercaptoethanol

$CDCl_3$ = deuterated chloroform

CHY = chymotrypsin

ddH_2O = double-distilled water

DMAP = dimethylaminopyridine

DCM = dichloromethane

DIPEA = *N,N*-diisopropylethylamine

DMSO = dimethyl sulfoxide

$DNA_{catalyst}$ = complementary DNA strand with bound catalyst

DNA_{temp} = template DNA strand

DTT = dithiothreitol

EtOAc = ethyl acetate

ESI = Electron Spray Ionisation

FA = formic acid

GRX = Glutaredoxin 1

HEPES = 4-(2-hydroxyethyl)-1-piperazineethanesulfonic acid

hGQ = hemin/G-Quadruplex

HPLC = high-performance liquid chromatography

IAA = iodoacetic acid

LC-MS = liquid chromatography–mass spectrometry

^{LC} = on the light chain

MeOD = methanol-d_4

NML = *N*-Methylluminol

PyOx = pyridinium oxime

SAA = surface accessible area

SDS-PAGE = sodium dodecyl sulfate–polyacrylamide gel electrophoresis

SPAAC = strain-promoted alkyne-azide click

TAMN = (0.5% trifluoroacetic acid + 30% ACN + 70% ddH_2O + 400 mM NaCl)

Thrombin = human alpha thrombin

THPTA = Tris(3-hydroxypropyltriazolylmethyl)amine

General Information

1.1 Materials

- Solvents were purchased from VWR-TS and used without prior purification, unless otherwise specified.
- Reagents were purchased from Sigma Aldrich and used without prior purification, unless otherwise specified.
- Sulforhodamine B PEG_3 azide was purchased from Tenova Pharmaceuticals.
- BCN-PEG(2000) was purchased from Synaffix BV.
- Glutaredoxin 1 was purchased from Sanbio B.V.
- Chymotrypsin was purchased from Sigma Aldrich.
- Human alpha-thrombin was purchased from Haematologic Technologies.
- Trypsin Gold was purchased from PROMEGA BENELUX BV.
- DNA strands were purchased from Integrated DNA technologies.
- MabPac™ RP Column (4 µm, 3.0 mm x 100 mm) was purchased from Thermo Fischer.
- Reprosil-Gold 300 C4, 3 µm. 250 mm x 4.0 mm was purchased from Screening Devices.
- HPLC runs were performed using a Finnigan Surveyor Plus HPLC system (Thermo Fisher).
- MS measurements were performed using a Q Exactive Mass Spectrometer (Thermo Fisher).
- Absorption measurements to determine DNA and protein concentrations were performed using a Scientific™ Nanodrop 2000 (Thermo Fisher).

1.2 DNA sequences

Table S1. The codes and nucleobase sequences for each DNA strand used in this study. "5Hexynyl" indicates a hexynyl attached at the 5' end; "35OctdU" indicates a 3' end thymine nucleobase with a octynyl attached; "i5OctdU" indicates an internal thymine nucleobase with an octynyl attached.

Code	DNA Sequence (5' to 3')
**DNA_{temp}**	AAA ATA TAT ATA TAT AAA A
**alkyne-DNA_{temp}**	/5Hexynyl/ AAA ATA TAT ATA TAT AAA A
**$DNA_{catalyst}$**	TTT TAT ATA TAT ATA TTT T
**DNA_{cat}-T1-alkyne**	TTT TAT ATA TAT ATA TTT /35OctdU/
**DNA_{cat}-T2-alkyne**	TTT TAT ATA TAT ATA TT/i5OctdU/ T
**DNA_{cat}-T3-alkyne**	TTT TAT ATA TAT ATA T/i5OctdU/T T
**DNA_{cat}-T4-alkyne**	TTT TAT ATA TAT ATA /i5OctdU/TT T
**DNA_{cat}-T6-alkyne**	TTT TAT ATA TAT A/i5OctdU/A TTT T
**DNA_{cat}-T8-alkyne**	TTT TAT ATA TA/i5OctdU/ ATA TTT T
**PW17**	GGG TAG GGC GGG TTG GG
**DNA_{cat}-T0-PW17**	TTTTATATATATATATTTT GGGTAGGGCGGGTTGGG
**DNA_{cat}-T4-PW17**	TTTTATATATATATA GGGTAGGGCGGGTTGGG
**DNA_{cat}-T8-PW17**	TTTTATATATA GGGTAGGGCGGGTTGGG
**DNA_{cat}-T10-PW17**	TTTTATATAT GGGTAGGGCGGGTTGGG ATATATTTT
**DNA_{cat}-T20-PW17**	GGGTAGGGCGGGTTGGG TTTTATATATATATATTTT

Table S2. The DNA duplexes used in this study with their calculated melting temperatures and the codes of the single strands. Values calculated using the NUPACK web application (http://www.nupack.org/, on 18-04-2022).

dsDNA-catalyst	ssDNA's	T_{melt}
**dsDNA-diPyOx**	DNA_{temp}<br>$DNA_{catalyst}$	**52°C**
**dsDNA-diDMAP**	DNA_{temp}<br>$DNA_{catalyst}$	**52°C**
**dsDNA-T0-PW17**	DNA_{temp}<br>DNA_{cat}-T0-PW17	**52°C**
**dsDNA-T4-PW17**	DNA_{temp}<br>DNA_{cat}-T4-PW17	**41°C**
**dsDNA-T8-PW17**	DNA_{temp}<br>DNA_{cat}-T8-PW17	**26°C**
**dsDNA-T10-PW17**	DNA_{temp}<br>DNA_{cat}-T10-PW17	**30°C**
**dsDNA-T20-PW17**	DNA_{temp}<br>DNA_{cat}-T20-PW17	**51°C**

Experimental Procedures

2.1 Synthesis of Organic Compounds

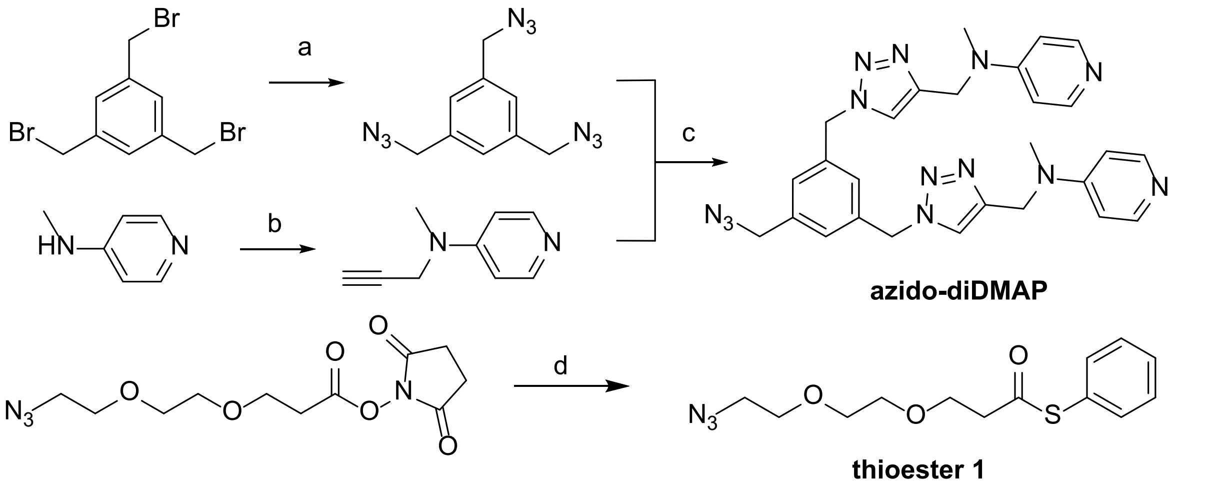

*Scheme S1. Synthesis of azido-diDMAP and thioester **1**. (a) NaN_3, DMF, rt, 16 h, **98%**; (b) n-BuLi, propargylbromide, anh. THF, -80-0 °C, 30 min, **57%**; (c) Cu(I)(ACN)$_4$PF$_6$, O_2-poor THF, rt, 18 h, **11%**; (d) thiophenol, toluene, rt, 16 h, **85%**.*

2.1.1 1,3,5-tris(azidomethyl)benzene:

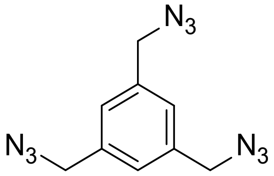

1,3,5-tris(bromomethyl)benzene (200 mg, 560 µmol, 1.0 eq) and sodium azide (219 mg, 3.36 mmol, 6.0 eq) were dissolved in 0.5 mL DMF and stirred at rt for 16 h. The mixture was blow-dried and the residue dissolved in 1 mL DCM. The mixture was washed with H_2O and extracted with DCM (3 × 5 mL). The organic layer was dried over Na_2SO_4 and concentrated under reduced pressure, yielding a clear oil (134 mg, 98%). ^{1}H NMR (400 MHz, $CDCl_3$) δ 7.24 (s, 3H), 4.38 (s, 6H) ppm. ^{13}C NMR (101 MHz, $CDCl_3$) δ 137.0, 127.4, 54.3 ppm.

2.1.2 alkyne-DMAP:

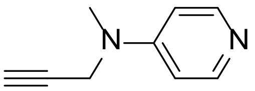

4-(methylamino)-pyridine (200 mg, 1.9 mmol, 1.0 eq) was placed in a flame-dried 25 mL flask under argon and dissolved in anhydrous THF (1.5 mL). The solution was cooled to -90 °C and n-butyllithium (0.8 mL, 2.0 mmol, 1.1 eq) was added and the mixture was stirred for 15 min @ -90 °C. Propargyl bromide (200 µL, 2.77 mmol, 1.5 eq) was added and the reaction mixture was stirred for 15 min at -90 °C and 15 min at 0 °C. The mixture was quenched with sat. NH_4Cl (5 mL) and extracted with THF (3 × 6 mL). The organic layer was dried over Na_2SO_4 and concentrated under reduced pressure. The residue was purified by flash column chromatography (SiO_2, 5% (10% [25% NH_3 in H_2O] in MeOH) in EtOAc) yielding a brown oil (152.8 mg, 57%). HRMS (ESI) calculated for $[M+H]^+$: 147.0922; found $[M+H]^+$: 147.0917. ^{1}H NMR (400 MHz, $CDCl_3$) δ 8.13 (s, 2H), 6.44 (d, J = 5.8 Hz, 2H), 3.90 (d, J = 2.6 Hz, 2H), 2.85 (s, 3H), 2.14 (t, J = 2.5 Hz, 1H) ppm. ^{13}C NMR (101 MHz, $CDCl_3$) δ 153.0, 149.7, 107.4, 78.0, 72.4, 40.5, 37.4 ppm.

2.1.3 azido-diDMAP:

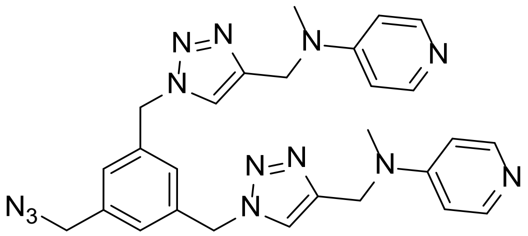

1,3,5-tris(azidomethyl)benzene (30 mg, 123 µmol, 1.0 eq), *N*-methyl-*N*-(prop-2-yn-1-yl)pyridin-4-amine (36 mg, 247 µmol, 2.0 eq) and diisopropylethylamine (107 µL, 617 µmol, 5.0 eq) were dissolved in acetonitrile 3 mL) and the mixture was bubbled with argon for 30 minutes. Tetrakis(acetonitrile)copper(I) hexafluorophosphate (230 mg, 617 µmol, 5.0 eq) was added and the reaction was stirred at rt overnight. The mixture was washed with 10% 3M NaOH in brine (5 mL) and extracted with EtOAc (3 × 10 mL). The organic layer was dried over Na_2SO_4 and concentrated under reduced pressure. The residue was purified via flash column chromatography (SiO_2, 8-12% (10% [25% NH_3 in H_2O] in MeOH) in DCM)) yielding a pale yellow solid (7 mg, 11 %). HRMS (ESI) calculated for $[M+H]^+$: 536.2747; found $[M+H]^+$: 536.2740. ^1H NMR (400 MHz, CD_3CN) δ 8.14 (s, 4H), 7.70 (s, 2H), 7.19 (d, *J* = 1.7 Hz, 2H), 7.13 (d, *J* = 1.8 Hz, 1H), 6.75 (s, 4H), 5.49 (s, 4H), 4.66 (s, 4H), 4.35 (s, 2H), 3.08 (s, 6H) ppm. ^{13}C NMR (101 MHz, CD_3CN) δ 155.0, 148.6, 144.7, 138.5, 138.2, 128.5, 128.0, 123.7, 108.3, 54.5, 53.8, 47.4, 38.2 ppm.

2.1.4 azido-thioester (**1**):

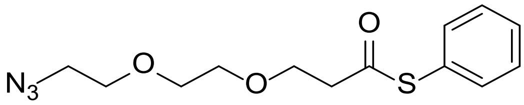

2,5-dioxopyrrolidin-1-yl 3-(2-(2-azidoethoxy)ethoxy)propanoate (150 mg, 500 µmol, 1.03 eq) was dissolved in 2 mL toluene. Thiophenol (49 µL, 480 µmol, 1.0 eq) was added. Triethylamine (81 µL, 580 µmol, 1.2 eq) was added dropwise. The mixture was stirred at rt for 16 h. The reaction was quenched with brine (8 mL) and the product was extracted with EtOAc (3 × 6 mL). The organic layer was then dried over Na_2SO_4 and concentrated under reduced pressure. The residue was purified via flash column chromatography (SiO_2, 20% EtOAc in petroleum ether (40/60)), yielding a colourless oil (121 mg, 85%). HRMS (ESI) calculated for $[M+Na]^+$: 318.0888; found $[M+Na]^+$: 318.0877. ^1H NMR (400 MHz, $CDCl_3$) δ 7.45–7.35 (m, 5H), 3.81 (t, *J* = 6.3 Hz, 2H), 3.71–3.58 (m, 6H), 3.35 (t, *J* = 5.1 Hz, 2H), 2.92 (t, *J* = 6.3 Hz, 2H) ppm. ^{13}C NMR (101 MHz, $CDCl_3$) δ 195.3, 134.4, 129.4, 129.1, 127.6, 70.5, 70.5, 70.0, 66.6, 50.6, 43.9 ppm.

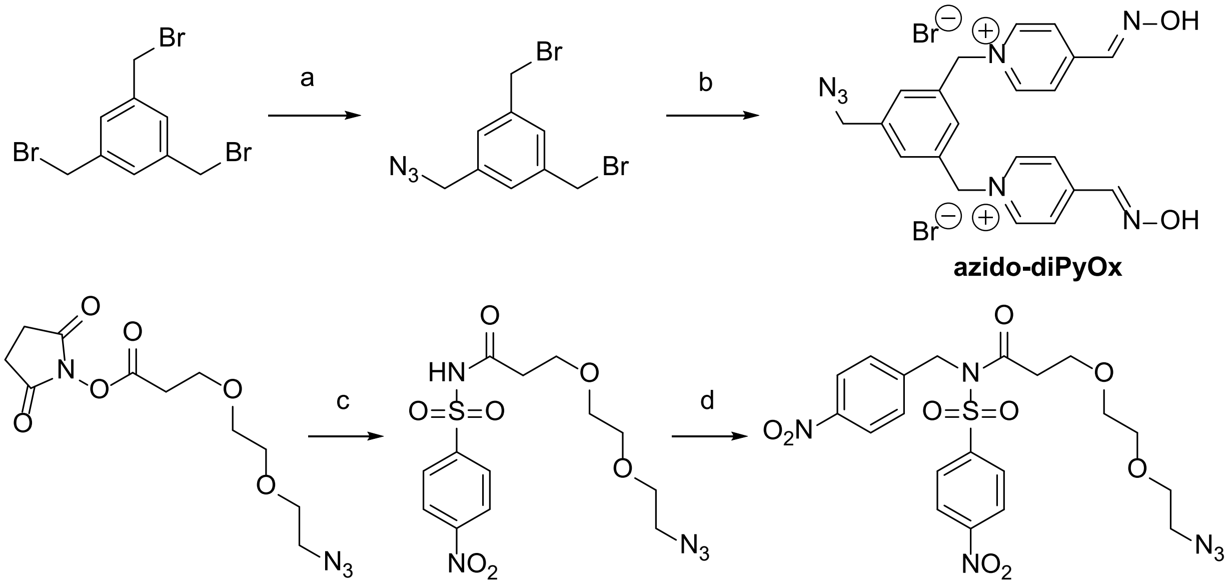

ANANS (2)

*Scheme S2. Synthesis of azido-diPyOx and ANANS **2**. (a) NaN_3, DMF, rt, 16 h, **57%**; (b) pyridine-4-aldoxime, ACN, 65 °C, 32 h, **64%**; (c) 4-nitrobenzenesulfonamide, DIPEA, DCM, rt, 20 h, **90%**; (d) DIPEA, 1-(bromomethyl)-4-nitrobenzene, anh. THF, 50 °C, 17 h, **72%**.*

2.1.5 1-(azidomethyl)-3,5-bis(bromomethyl)benzene:

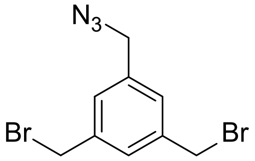

1,3,5-tris(bromomethyl)benzene (30 mg, 84 µmol, 1.0 eq) and sodium azide (5.5 mg, 84 µmol, 1.0 eq) were dissolved in 100 µL DMF and stirred at rt for 16 h. The mixture was blow-dried and the residue dissolved in 400 µL DCM. The products were separated by means of preparative TLC (5% diethylether in petroleum ether(40-60)) and the desired product recovered with diethyl ether, filtered and concentrated under reduced pressure, yielding a white solid (15.1 mg, 57%). HRMS (ESI) calculated for $[M+H]^+$: 317.9241 / 319.9240; found $[M+H]^+$: 317.9318 / 319.9297. ^1H NMR (400 MHz, $CDCl_3$) δ 7.39 (s, 1H), 7.27 (d, J = 8.3 Hz, 2H), 4.46 (d, J = 7.5 Hz, 4H), 4.37 (s, 2H) ppm. ^{13}C NMR (101 MHz, $CDCl_3$) δ 139.3, 137.1, 129.6, 128.7, 54.3, 32.4 ppm.

2.1.6 azido-diPyOx:

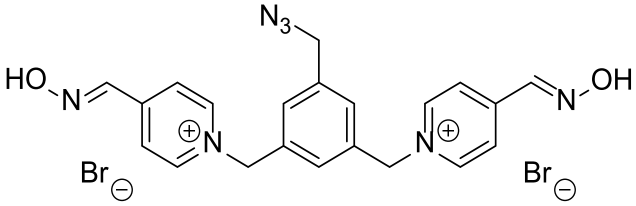

1-(azidomethyl)-3,5-bis(bromomethyl)benzene (8.0 mg, 25 µmol, 1.0 eq) and pyridine-4-aldoxime (15 mg, 125 µmol, 5 eq.) were dissolved in 500 µL ACN and the mixture was stirred at 65 °C for 32 h. The mixture was transferred to an Eppendorf tube, washing the vessel with ACN and centrifuged with a tabletop centrifuge. The ACN was carefully removed and the solid washed with clean ACN (3 × 1 mL). The residue was allowed to dry overnight, yielding a brown

solid (6.5 mg, 64%). HRMS (ESI) calculated for $[M–H]^+$: 402.1678; found $[M–H]^+$: 402.1678. ^1H NMR (400 MHz, D_2O) δ 8.86 (dt, *J* = 9.9, 4.8 Hz, 4H), 8.39 (d, *J* = 2.3 Hz, 2H), 8.25–8.19 (m, 4H), 7.55 (d, *J* = 2.8 Hz, 2H), 7.51 (s, 1H), 5.86 (d, *J* = 2.4 Hz, 4H), 4.49 (d, *J* = 2.3 Hz, 2H) ppm. ^{13}C NMR (101 MHz, D_2O) δ 149.3, 146.2, 144.8, 144.7, 138.7, 134.8, 129.9, 129.0, 125.0, 63.4, 53.3 ppm.

2.1.7 azido-ANANS precursor:

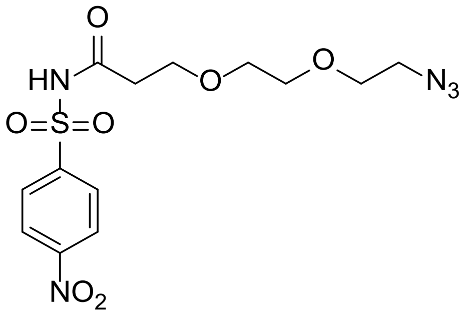

4-nitrobenzenesulfonamide (162 mg, 799 µmol, 1.2 eq) was dissolved in 1 mL DCM and *N,N*-diisopropylethylamine (232 µL, 1.33 mmol, 2.0 eq) was added to the mixture and stirred for 5 min. 2,5-dioxopyrrolidin-1-yl 3-(2-(2-azidoethoxy)ethoxy)propanoate (200 mg, 666 µmol, 1.0 eq) was added and the mixture was stirred at rt for 20 h. The reaction was washed with 1 M HCl (8 mL) and the product extracted with DCM (3 × 8 mL). The organic layer was washed with brine (25 mL) and the product extracted with DCM (2 × 15 mL). The organic layer was dried over Na_2SO_4 and concentrated under reduced pressure. The residue was purified via flash column chromatography (SiO_2, starting with 3% [10% (25% NH_3 in H_2O) in MeOH] in DCM, then 5% MeOH in DCM), yielding a yellow oil (233 mg, 90%). HRMS (ESI) calculated for $[M+H]^+$: 410.0746; found $[M+H]^+$: 410.0730. ^1H NMR (400 MHz, $CDCl_3$) δ 8.23 (d, *J* = 8.5 Hz, 2H), 8.16 (d, *J* = 8.4 Hz, 2H), 3.60 (dt, *J* = 12.1, 5.4 Hz, 8H), 3.45 (t, *J* = 4.9 Hz, 2H), 2.49 (t, *J* = 5.7 Hz, 2H) ppm. ^{13}C NMR (101 MHz, $CDCl_3$) δ 177.3, 149.8, 147.9, 128.7, 123.9, 70.3, 70.0, 69.7, 67.7, 50.7, 39.0 ppm.

2.1.8 azido-ANANS (**2**):

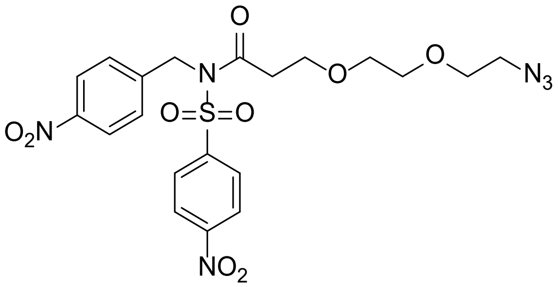

3-(2-(2-azidoethoxy)ethoxy)-*N*-((4-nitrophenyl)sulfonyl)propanamide (120 mg, 307 µmol, 1.0 eq) was dissolved in 1 mL anh. THF. *N,N*-diisopropylethylamine (268 µL, 1.54 mmol, 5.0 eq) was added to the mixture and stirred at rt for 5 min. 1-(bromomethyl)-4-nitrobenzene (265 mg, 1.23 mmol, 4.0 eq) was dissolved in 0.7 mL anh. THF and added to the solution and the mixture was stirred at 50 °C for 17 h. The mixture was washed with brine (5 mL) and extracted with EtOAc (3 × 5 mL). The organic layer was dried over Na_2SO_4 and concentrated under reduced pressure. The residue was purified via flash column chromatography (SiO_2, 30–40% EtOAc in petroleum ether(40-60)) yielding a yellow oil (121 mg, 76%). HRMS (ESI) calculated for $[M+H]^+$: 545.1067; found $[M+H]^+$: 545.1049. ^1H NMR (400 MHz, $CDCl_3$) δ 8.38 (d, *J* = 8.5 Hz, 2H), 8.23 (d, *J* = 8.4 Hz, 2H), 8.11 (d, *J* = 8.6 Hz, 2H), 7.55 (d, *J* = 8.3 Hz, 2H), 5.17 (s, 2H), 3.70 (t, *J* =

6.0 Hz, 2H), 3.62 (t, J = 4.9 Hz, 2H), 3.54 (q, J = 5.3, 4.2 Hz, 4H), 3.35 (t, J = 4.9 Hz, 2H), 2.84 (t, J = 6.0 Hz, 2H) ppm. ^{13}C NMR (101 MHz, $CDCl_3$) δ 171.4, 150.9, 147.8, 144.7, 143.3, 129.5, 128.4, 124.6, 124.2, 70.6, 70.1, 66.4, 50.8, 49.5, 37.0 ppm.

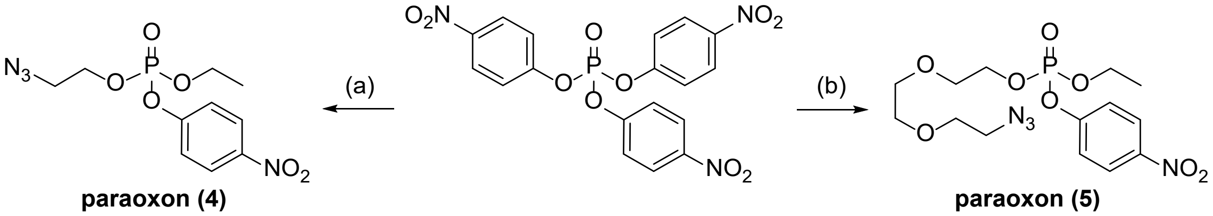

Scheme S3. Synthesis of the two azido-functionalized paraoxon species. (a) 2-azidoethanol, DBU, DCM, EtOH, 0°C, 2 h, ***35%****; (b) 2-(2-(2-azidoethoxy)ethoxy)ethanol, DBU, DCM, EtOH, 0°C, 2 h,* ***25%****.*

2.1.9 azido-ethyl-paraoxon (**4**):

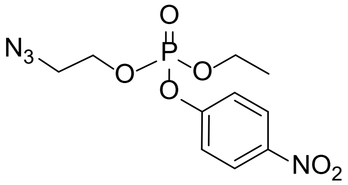

Tris(4-nitrophenyl) phosphate (277 mg, 600 μmol, 1.2 eq.) and 2-azidoethanol (47 μL, 550 μmol, 1.1 eq.) were dissolved in DCM (12 mL) and stirred at 0°C for 5 min. 1,8-Diazabicyclo[5.4.0]undec-7-ene (75 μL, 500 μmol, 1.0 eq) was dissolved in DCM (0.3 mL) and added dropwise to the reaction mixture after which it was stirred for 1 h. EtOH (59 μL, 1.0 mmol, 2.0 eq.) was added to the reaction mixture. 1,8-Diazabicyclo[5.4.0]undec-7-ene (75 μL, 500 μmol, 1.0 eq) was dissolved in DCM (0.3 mL) and added dropwise to the reaction mixture after which it was stirred for 1 h. The reaction mixture was concentrated by blow-drying and purified via flash column chromatography (SiO_2, 40% EtOAc in petroleum ether (40/60)), yielding a colourless oil (55 mg, 35%). ^{1}H NMR (400 MHz, $CDCl_3$) δ 8.31–8.19 (m, 2H), 7.45–7.34 (m, 2H), 4.38–4.19 (m, 4H), 3.58–3.49 (m, 2H), 1.39 (td, J = 7.1, 1.2 Hz, 3H) ppm. ^{13}C NMR (101 MHz, $CDCl_3$) δ 155.4, 145.0, 125.9, 120.7, 67.2, 65.8, 50.9, 16.2 ppm.

2.1.10 azido-EG$_2$-paraoxon (**5**):

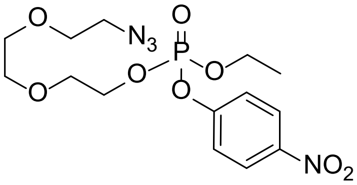

Tris(4-nitrophenyl) phosphate (277 mg, 600 μmol, 1.2 eq.) and 2-(2-(2-azidoethoxy)ethoxy)ethanol (96 mg, 550 μmol, 1.1 eq.) were dissolved in DCM (12 mL) and stirred at 0 °C for 5 min. 1,8-Diazabicyclo[5.4.0]undec-7-ene (75 μL, 500 μmol, 1.0 eq) was dissolved in DCM (0.3 mL) and added dropwise to the reaction mixture after which it was stirred for 1 h. EtOH (160 μL, 2.0 mmol, 4.0 eq.) was added to the reaction mixture. 1,8-Diazabicyclo[5.4.0]undec-7-ene (95 μL, 630 μmol, 1.2 eq) was dissolved in DCM (0.3 mL) and added dropwise to the reaction mixture after which it was stirred for 1 h. The reaction mixture was concentrated by blow-drying and purified via flash column chromatography (SiO_2, 50% EtOAc in petroleum ether (40/60)) and (SiO_2, 2% MeOH in DCM), yielding a colourless oil (50 mg, 25%). ^{1}H NMR (400 MHz, $CDCl_3$) δ 8.28–8.12 (m, 2H), 7.38 (dd, J = 9.2, 1.0 Hz, 2H),

4.33–4.20 (m, 4H), 3.72 (ddd, *J* = 5.5, 4.1, 1.5 Hz, 2H), 3.64–3.60 (m, 6H), 3.35 (t, *J* = 5.0 Hz, 2H), 1.35 (td, *J* = 7.1, 1.2 Hz, 3H) ppm. ^{13}C NMR (101 MHz, $CDCl_3$) δ 155.6, 144.8, 125.7, 120.8, 70.7, 70.2, 70.0, 67.9, 65.4, 61.8, 50.7, 14.2 ppm.

2.2 Synthesis of protein-DNA_{temp} and $DNA_{catalyst}$

2.2.1 Synthesis of GRX-DNA_{temp}

Glutaredoxin 1 was incubated with DTT (1.5 eq.) in 15 mM NH_4HCO_3 (pH: 8.0) at 37 °C for 30 min, followed by incubation with 1-(azidomethyl)-3,5-bis(bromomethyl)benzene (2.5 eq.) at 37 °C for 2.5 h and subsequently purified by spin filtration over 3 kDa MWCO Amicon® Ultra-15 Centrifugal Filter Units, washing 3 times with 50 mM NaCl solution in 25 mM Tris (pH: 8.0). The synthesized azido-protein was treated with 2 equivalents of DNA_{temp} (from 500 µM stock in oxygen-poor ddH_2O) with respect to the protein concentration, 100 µM [Cu•THPTA] (complex of $CuSO_4$ and THPTA mixed in a ratio of 1:5 in ddH_2O) and 10 mM sodium ascorbate (from a freshly made stock of 100 mM in oxygen-poor ddH_2O) and incubated in the dark at 12 °C for 16–20 hours. The formed GRX-DNA_{temp} construct was purified by FPLC, using an ion-exchange MonoQ column (Vol: 1 mL) using a gradient from 0–1 M NaCl in 20 mM Tris (pH: 8.0). The collected fractions were and concentrated by spin filtration over 3 kDa MWCO Amicon® Ultra-15 Centrifugal Filter Units, washing 3 times with 50 mM NaCl solution in 25 mM Tris (pH: 8.0). The concentration of GRX-DNA_{temp} was quantified from absorption values determined with a Scientific^{TM} Nanodrop 2000, using a 1:1 mixture of native GRX with DNA_{temp} as a reference.

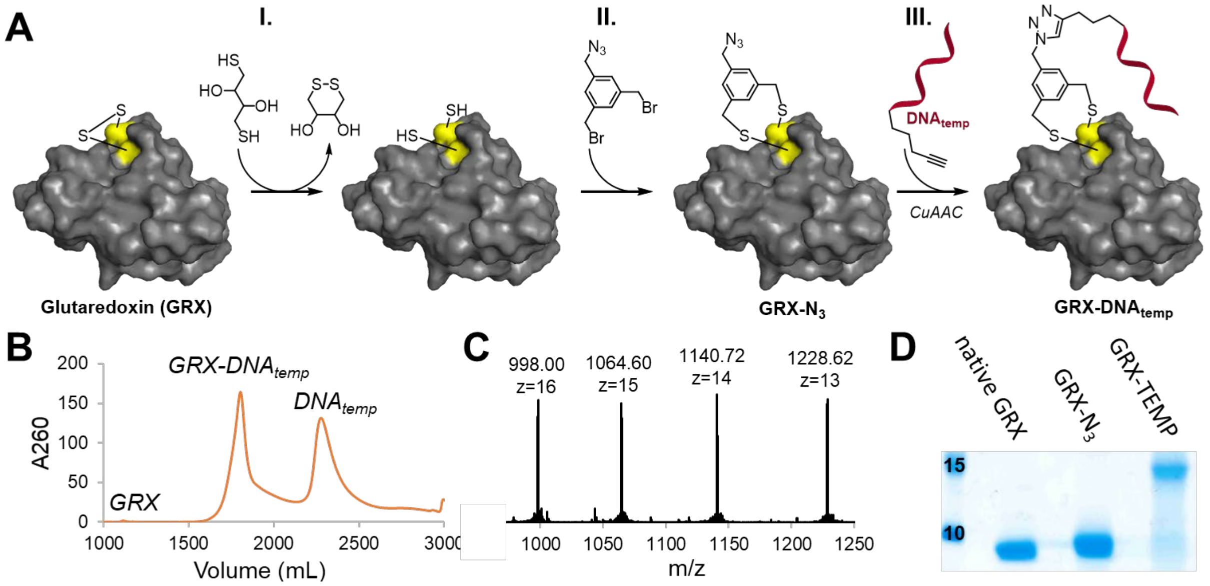

*Figure S1. Preparation of the building blocks used to calibrate our catalytic DNA systems. (**A**) Synthesis of GRX-DNA_{temp} by means of (I) DTT-mediated opening of the disulfide bridge, (II) insertion of 1-azidomethyl-3,5-bis(bromomethyl)benzene to install a uniquely reactive azide at the active site, and (III) attachment of alkyne-DNA_{temp} by means of copper-catalysed click. (**B**) FPLC trace of the separation of GRX, GRX-DNA_{temp} and DNA_{temp} by ion-exchange (detection at 260 nm). (**C**) Mass spectrometry data of GRX-DNA_{temp} (calculated peaks: 998.0(z=16), 1064.6(z=15), 1140.7(z=14), 1228.3(z=13)). (**D**) SDS-PAGE analysis of native GRX, azide-functionalized GRX and the GRX-DNA_{temp} conjugate, which has an upward shift of ~6 kDa.*

2.2.2 Synthesis of CHY-DNA$_{temp}$ and TRM-DNA$_{temp}$

Thrombin or Chymotrypsin was incubated with azido-paraoxon (20 eq.) in 20% glycerol in 50 mM HEPES (pH: 7.2) at 37 °C for 3 h and subsequently purified by spin filtration over 10 kDa MWCO Amicon® Ultra-15 Centrifugal Filter Units, washing 3 times with 20% glycerol in 50 mM NaCl solution in 25 mM Tris (pH: 8.0) for thrombin and 50 mM NaCl solution in 25 mM Tris (pH: 8.0) for chymotrypsin. The respective azido-protein was treated with 2–3 equivalents of DNA$_{temp}$ (from 500 µM stock in oxygen-poor ddH$_2$O) with respect to the protein concentration, 100–150 µM [Cu•THPTA] (complex of $CuSO_4$ and THPTA mixed in a ratio of 1:5 in ddH$_2$O) and 7–10 mM sodium ascorbate (from a freshly made stock of 100 mM in oxygen-poor ddH$_2$O) and incubated in the dark at 12 °C for 16–20 hours. The synthesized protein-DNA$_{temp}$ constructs were purified by spin filtration over 10 kDa MWCO Amicon® Ultra-15 Centrifugal Filter Units, washing 3 times with 20% glycerol in 50 mM NaCl solution in 25 mM Tris (pH: 8.0) for thrombin and 50 mM NaCl solution in 25 mM Tris (pH: 8.0) for chymotrypsin. The concentrations were quantified from absorption values determined with a Scientific™ Nanodrop 2000, using a 1:1 mixture of native protein with DNA$_{temp}$ as a reference.

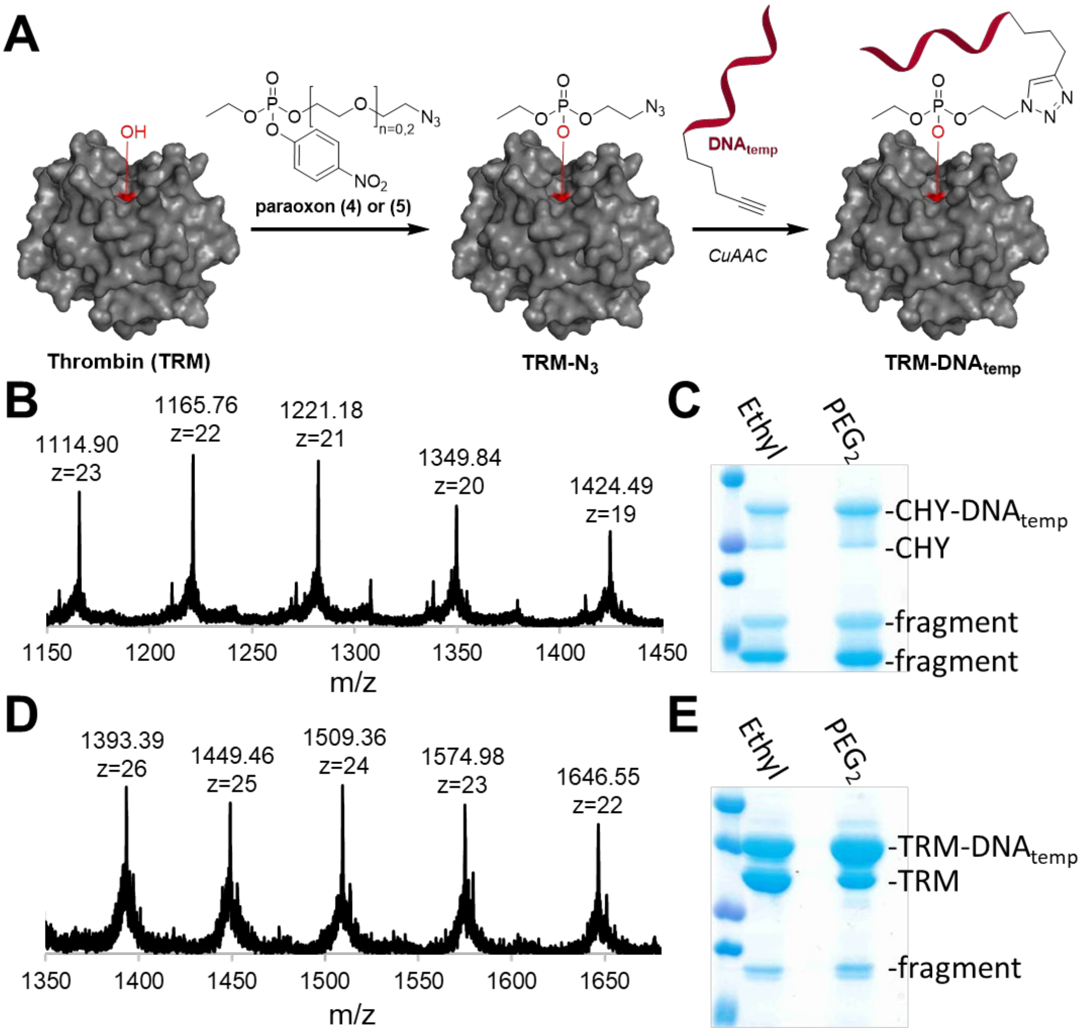

*Figure S2 (**A**) Synthesis of thrombin-DNA (TRM-DNA$_{temp}$) and chymotrypsin-DNA (CHY-DNA$_{temp}$) by using paraoxon derivative **1** (or **2**); (**B**) Mass spectrometry data of CHY-Et-N$_3$ (calculated peaks: 1115.0(z=23), 1165.7(z=22), 1221.2(z=21), 1282.2(z=20), 1349.8(z=19), 1424.5(z=18)); (**C**) CHY-DNA$_{temp}$ synthesized with the ethyl or EG$_2$ linker; (**D**) Mass spectrometry data of TRM-Et-N$_3$ (calculated peaks: 1393.4(z=26), 1449.1(z=25), 1509.3(z=24), 1575.0(z=23), 1646.5(z=22)); (**E**) SDS-PAGE result of synthesized TRM-DNA$_{temp}$ with ethyl or EG$_2$ linker.*

2.2.3 Synthesis of DNA_{DMAP} and DNA_{PyOx}

DNA-alkyne sequences with alkyne-thymine modification were purchased as HPLC-purified lyophilized powders from Integrated DNA technologies. The powders were dissolved in oxygen-poor ddH_2O. The DNA was treated with 10 equivalents of compound azido-diDMAP or azido-diPyOx (from 100 mM stock in DMSO) with respect to the DNA concentration, 100 µM [Cu•THPTA] (complex of $CuSO_4$ and THPTA mixed in a ratio of 1:5 in ddH_2O) and 10 mM sodium ascorbate (from a freshly made stock of 100 mM in ddH2O) and incubated in the dark at 12 °C for 16–20 hours. The synthesized $DNA_{catalyst}$ construct was purified by spin filtration over 3 kDa MWCO Amicon® Ultra-15 Centrifugal Filter Units, washing 3 times with 400 mM NaCl solution in ddH_2O. Purity and concentration were determined by HPLC-MS and UV-Vis.

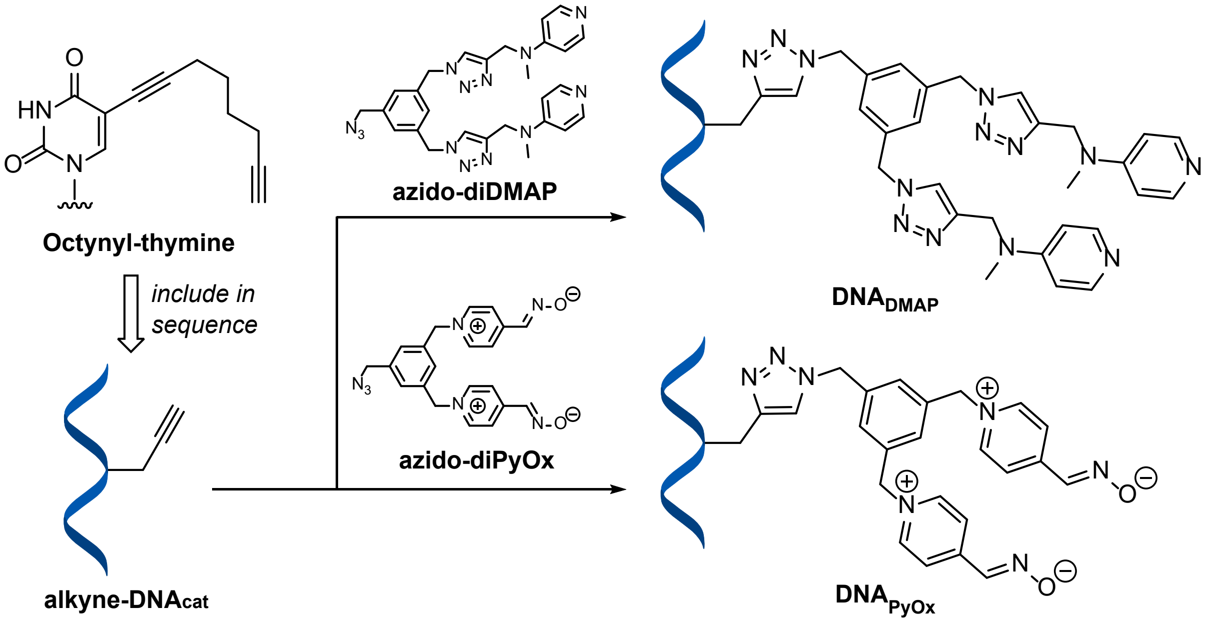

Scheme S4. Syntheses of $DNA_{catalyst}$ constructs. Alkyne-DNA_{temp} and azido-diDMAP or azido-diPyOx are coupled via copper-catalysed alkyne-azide click chemistry and purified via spin filtration.

Table S3. Lengths of all components in this work. Values were estimated using YASARA and with each molecule in the utmost linear state. The independent linker lengths include also the hexynyl chain of the DNA, thus indicating the distance between protein surface and the 5' end of DNA_{temp}.

Code	Length (nm)	Code	Length (nm)
GRX-DNA(T1)	2.2	*CHY/TRM-EG$_2$-DNA(T1)*	2.5
GRX-DNA(T2)	2.5	*CHY/TRM-EG$_2$-DNA(T2)*	2.8
GRX-DNA(T3)	2.9	*CHY/TRM-EG$_2$-DNA(T3)*	3.2
GRX-DNA(T4)	3.2	*CHY/TRM-EG$_2$-DNA(T4)*	3.5
GRX-DNA(T6)	3.9	*CHY/TRM-EG$_2$-DNA(T6)*	4.2
GRX-DNA(T8)	4.6	*CHY/TRM-EG$_2$-DNA(T8)*	4.9
CHY/TRM-Et-DNA(T1)	2.0	*GRX linker*	1.8
CHY/TRM-Et-DNA(T2)	2.4	*Et linker*	1.7
CHY/TRM-Et-DNA(T3)	2.7	*EG$_2$ linker*	2.1
CHY/TRM-Et-DNA(T4)	3.1	*Octynyl (thymine linker)*	1.0
CHY/TRM-Et-DNA(T6)	3.8	*diDMAP (N$_3$ to acyl group)*	1.4
CHY/TRM-Et-DNA(T8)	4.5	*diPyOx (N$_3$ to acyl group)*	1.3

2.3 Protein Modification Studies

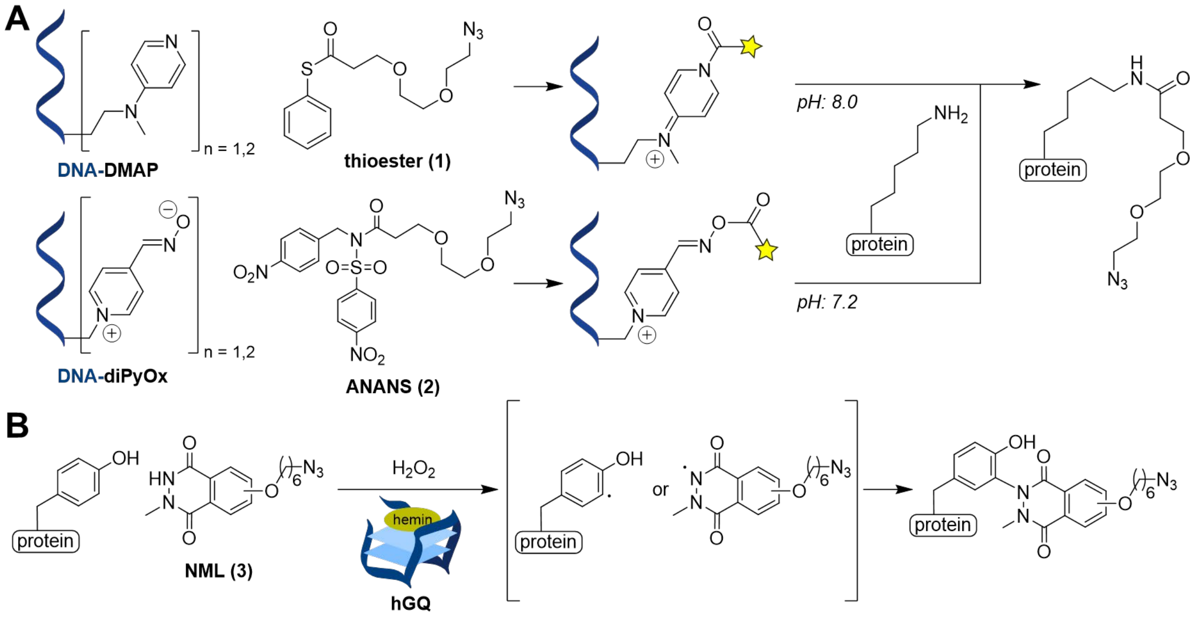

*Figure S3. The protein modification reactions used in this work. (**A**) DMAP and PyOx accept acyl groups from thioester **1** and ANANS **2**, respectively, and form an acylated intermediate that can be attack by Lys residues on the protein surface, resulting in stable peptide bonds. (**B**) Either Tyr residues or NML **3** are oxidatively activated by the hGQ DNAzyme in the presence of H_2O_2 and radically couple to one another.*

2.3.1 Protocol for modification of protein-DNA$_{temp}$ with DNA$_{DMAP}$

A mixture was typically prepared containing (a) 20 µM GRX-DNA$_{temp}$ (from 100 µM stock in 20 mM NH_4HCO_3 pH: 8.0) (b) 20–25 µM CHY-DNA$_{temp}$ (from 150-185 µM stock in 20 mM NH_4HCO_3 pH: 8.0) (c) 20–26 µM TRM-DNA$_{temp}$ (from a 150-200 µM stock solution in 20% glycerol in 20 mM NH_4HCO_3 pH: 8.0), 30 µM DNA$_{DMAP}$ (from varying stock concentrations in ddH_2O) in HEPES buffer [50 mM, pH: 8.0, with 350 mM NaCl and 50 mM KCl]. This mixture was incubated in the dark for 20–30 min at 37 °C, after which thioester **1** (from varying stock concentrations in DMSO) was added. The reaction mixture was incubated in the dark at 37 °C for 2 h, shaking the tubes at 500 rpm.

2.3.2 Protocol for modification of protein-DNA$_{temp}$ with DNA$_{PyOx}$

A mixture was typically prepared containing (a) 20 µM GRX-DNA$_{temp}$ (from 100 µM stock in 20 mM NH_4HCO_3 pH: 8.0) (b) 20–25 µM CHY-DNA$_{temp}$ (from 150-185 µM stock in 20 mM NH_4HCO_3 pH: 8.0) (c) 20–26 µM TRM-DNA$_{temp}$ (from a 150-200 µM stock solution in 20% glycerol in 20 mM NH_4HCO_3 pH: 8.0), 30 µM DNA$_{PyOx}$ (from varying stock concentrations in ddH_2O) in HEPES buffer [50 mM, pH: 7.2, with 350 mM NaCl and 50 mM KCl]. This mixture was incubated in the dark for 20–30 min at 37 °C, after which ANANS **2** (from varying stock concentrations in DMSO) was added. The reaction mixture was incubated in the dark at 37 °C for 6 h, shaking the tubes at 500 rpm.

2.3.3 Protocol for modification of protein-DNA$_{temp}$ with DNA$_{cat}$-hGQ

A mixture was typically prepared containing (a) 20 µM GRX-DNA$_{temp}$ (from 100 µM stock in 20 mM NH_4HCO_3 pH: 8.0) (b) 20–25 µM CHY-DNA$_{temp}$ (from 150-185 µM stock in 20 mM NH_4HCO_3 pH: 8.0) (c) 20–26 µM TRM-DNA$_{temp}$ (from a 150-200 µM stock solution in 20% glycerol in 20 mM NH_4HCO_3 pH: 8.0), 30 µM DNA$_{cat}$-PW17, 30 µM hemin (from 225 µM stock in DMSO) and 500 µM NML **3** (taken from a 7 mM stock solution in DMSO) in PO_4 buffer [50

mM, pH=7.0, with 400 mM NaCl and 5 mM KCl]. This mixture was allowed to stand for 20 min after which H_2O_2 (from 5 mM stock in in PO_4 buffer [50 mM, pH=7.0, with 800 mM NaCl and 10 mM KCl]) was added to a final concentration of 500 µM. The reaction mixture was then kept in the dark at 25 °C for 30 min, shaking the tubes at 500 rpm. Afterwards, the reaction was quenched by adding catalase to a final concentration of 0.01 mg/mL (from 0.2 mg/mL stock in $(NH_4)_2SO_4$ buffer).

2.3.2 Protocol for SDS-PAGE analysis

Prior to SDS-PAGE analysis, additional functionalization is required to visualize the modifications. Two approaches were used: band shifting or fluorescent staining. Band shifting: modified protein was treated with at least 6 equivalents of BCN-PEG_{2000} (purchased from Synaffix B.V.) with respect to the concentration of acyl donor and incubated at 12 °C overnight. Fluorescent staining: modified protein was treated with 6 equivalents of BCN-sulphorhodamine B with respect to the acyl donor and incubated at 12 °C overnight.

Acrylamide gels (12%) were prepared according to Bio-Rad bulletin 6201 protocol. Specifically, reaction mixtures containing 2–5 µg of protein were diluted with one volume equivalent of SDS-PAGE sample buffer (2×) containing 10% BME and incubated for 10 minutes at 95 °C. The denatured sample was then used for SDS-PAGE analysis (12% acrylamide gel). Precision Plus Protein™ Dual Color Standards was used as a reference protein ladder. After running, if one of the proteins was modified with a fluorophore, a UV-photo of the gel was taken. Gels were then stained using Coomassie brilliant blue (0.1% Coomassie Blue R250 in 10% acetic acid, 50% methanol and 40% demineralized water) by shaking gently for 0.5 hours, and destained with destaining solution (10% acetic acid, 50% methanol, and 40% demineralized water) by shaking gently for 1 hour. Afterwards, the destaining solution was replaced with H_2O and shaken gently overnight at room temperature. When the BCN-PEG_{2000} mass-tag was used, quantification was performed by integrating the intensity of the Coomassie stained bands of de SDS-PAGE gel using ImageJ software.

2.3.3 Protocol for the analysis of protein modification on HPLC(-MS)

The reaction mixture was aspirated three times with a pipette, after which 2–10 µL was added to an HPLC vial insert that already contained 18–10 µL of buffer (200 mM Citrate and 400 mM NaCl; pH: 5.5). The resulting mixture was also aspirated three times. This sample was then run over a Thermo Fischer MAbPAC RP column 3.0 × 100 mm, at 80 °C, the gradient varying per protein. For thrombin, the gradient started with 23% (ACN + 0.1% FA) ending with 33% (ACN + 0.1% FA) in (95% H_2O + 5% ACN + 0.1% FA) (flow rate 0.5 mL/min) over 25 min. The system used was an Agilent 1220 Infinity LC system with DAD detector.

For mass spectrometry analysis, reaction mixtures were diluted to a final protein concentration of 0.25 mg/mL. Protein samples were then analysed on a Thermo Scientific™ Q Exactive Focus Orbitrap using the same gradient as was used for the HPLC analyses.

2.3.4 Tryptic digestion MS/MS analysis of modified protein-DNA conjugates

Modified protein samples were subjected to SDS-PAGE separation and the desired protein bands cut from the gel and cut up to small pieces. The pieces were washed by incubating three times with 50 mM NH_4HCO_3 (pH: 8.0) in 50% ACN in ddH_2O and subsequently dried in a Speedyvac vacuum centrifuge. The dry pieces were swollen in 50 µL DTT [10 mM in 100 mM

NH_4HCO_3 (pH: 8.0)] and incubated for 45 minutes at 56 °C. The supernatant was removed and 50 µL of IAA (55 mM in 100 mM NH_4HCO_3 (pH: 8.0)) was added and the pieces were incubated in the dark at rt for 30 min. The supernatant was removed and the pieces were washed by incubating once with 50 mM NH_4HCO_3 (pH: 8.0) in 50% ACN in ddH_2O and subsequently dried in a vacuum centrifuge. The gel pieces were swollen in 40 µL trypsin gold (125 ng/µL) and incubated at 37 °C for 16–18 h. The initial supernatant was collected and the gel pieces were washed by incubating 15 min at 37 °C with 20 µL NH_4HCO_3 (100 mM, pH: 8.0) and 15 min at 37 °C when diluted with 20 µL. The collected supernatants were combined and dried in a vacuum centrifuge and the dry peptide digest dissolved in 20 µL 0.1% FA.

Peptide digests were analysed on an EASY nanoLC connected to Thermo Scientific^{TM} Q Exactive PLUS. Peptides were trapped onto a PepSep trap column (2 cm × 100 µm ID, 5 µm C18 ReproSil) and subsequently separated on a PepSep analytical column (8 cm × 75 µm ID, 3 µm C18 ReproSil, PepSep). Elution was achieved using a gradient that started with 5% (ACN + 0.1% FA) ending with 40% (ACN + 0.1% FA) in (H_2O + 0.1% FA), washing the column with 80% (ACN + 0.1% FA) afterwards.

The eluted peaks were analysed using MaxQuant software, searching for peptides with mass modification corresponding to H(11)O(3)C(7)N(3) (*i.e.*, the substitution of a proton on the protein by the acyl group of thioester **1** or ANANS **2**) and limiting criteria of 1% PSM FDR and a minimal peptide score of 80. As protein database human proteome was used, obtained from www.unitprot.org (code: UP000005640).

2.4 SDS-PAGE gel results

2.4.1 GRX-DNA$_{temp}$ with DNA$_{diDMAP}$ & 300 µM thioester

Lane#	1	2	3	4	5	6	7	8	9*	10*	11*
Cat pos	T1	T2	T3	T4	T6	T8	-	free	T8	free	-
**Conv%**	**100**	**100**	**100**	**98**	**90**	**74**	**-**	**31**	**27**	**24**	**-**

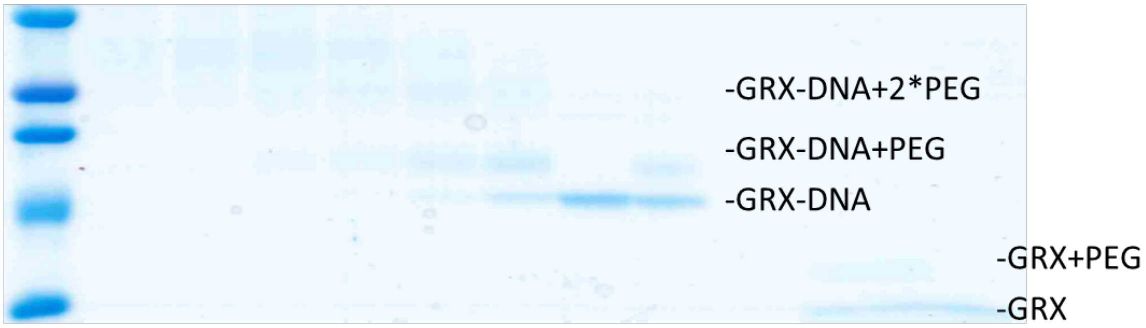

*Figure S4. GRX-DNA$_{temp}$ modified by DNA$_{DMAP}$ with thioester 1 and afterwards PEGylated with BCN-PEG$_{2000}$. Conversions were calculated using ImageJ. Conditions: 20 µM GRX-DNA$_{temp}$, 22 µM DNA$_{DMAP}$ and 300 µM thioester **1**, pH: 8.0, at 37 °C for 2 h.*

2.4.2 GRX-DNA$_{temp}$ with DNA$_{diDMAP}$ & 100 µM thioester

Lane#	1	2	3	4	5	6	7	8
Cat pos	T1	T2	T3	T4	T6	T8	-	free
**Conv%**	**62**	**56**	**60**	**52**	**44**	**36**	**-**	**15**

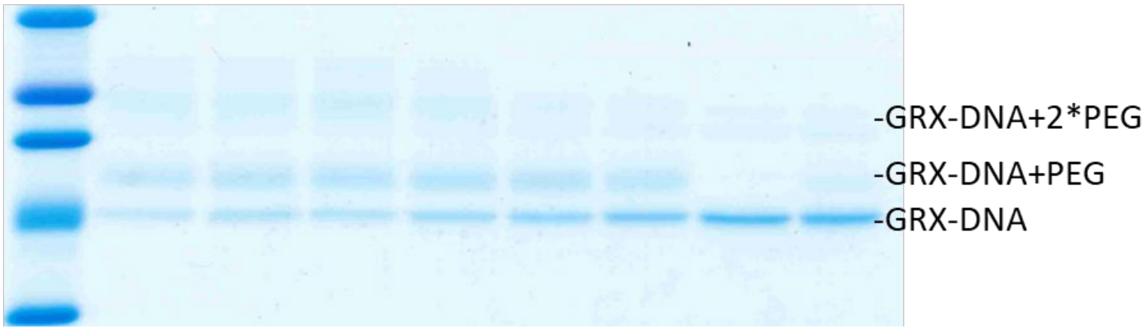

*Figure S5. GRX-DNA$_{temp}$ modified by DNA$_{DMAP}$ with thioester 1 and afterwards PEGylated with BCN-PEG$_{2000}$. Conversions were calculated using ImageJ. Conditions: 20 µM GRX-DNA$_{temp}$, 22 µM DNA$_{DMAP}$ and 100 µM thioester **1**, pH: 8.0, at 37 °C for 2 h.*

2.4.3 GRX-DNA$_{temp}$ with DNA$_{diPyOx}$

Lane#	1	2	3	4	5	6	7	8	9*	10*	11*
Cat pos	T1	T2	T3	T4	T6	T8	free	-	T8	free	-
**Conv%**	**65**	**62**	**56**	**54**	**34**	**16**	**0**	-	**1**	**0**	-

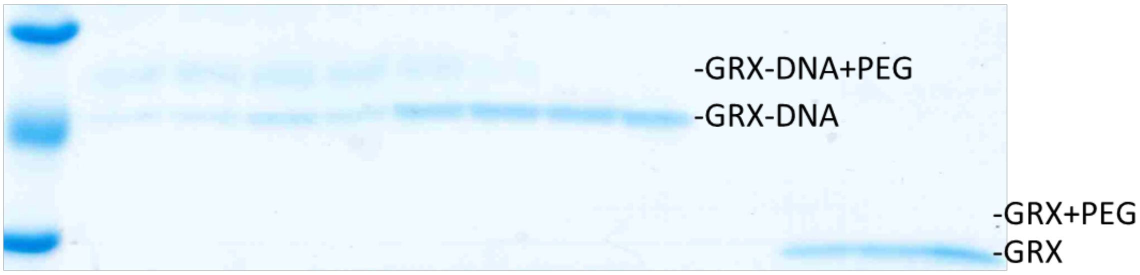

*Figure S6. GRX-DNA$_{temp}$ modified by DNA$_{PyOx}$ with ANANS **2** and afterwards PEGylated with BCN-PEG$_{2000}$. Conversions were calculated using ImageJ. Slots 9-11 contain native GRX. Conditions: 20 µM GRX-DNA$_{temp}$, 22 µM DNA$_{PyOx}$ and 300 µM ANANS **2**, pH: 7.2, at 37 °C for 6 h.*

2.4.4 GRX-DNA$_{temp}$ with DNA$_{cat}$-hGQ DNAzyme

Lane#	1	2	3	4	5	6	7	8	9*	10*	11*	12*
Cat pos	PW17	T20	T10	T4	T8	T0	hemin	-	PW17	T20	T0	-
**Conv%**	**32**	**54**	**44**	**43**	**60**	**48**	**6**	-	**9**	**9**	**8**	-

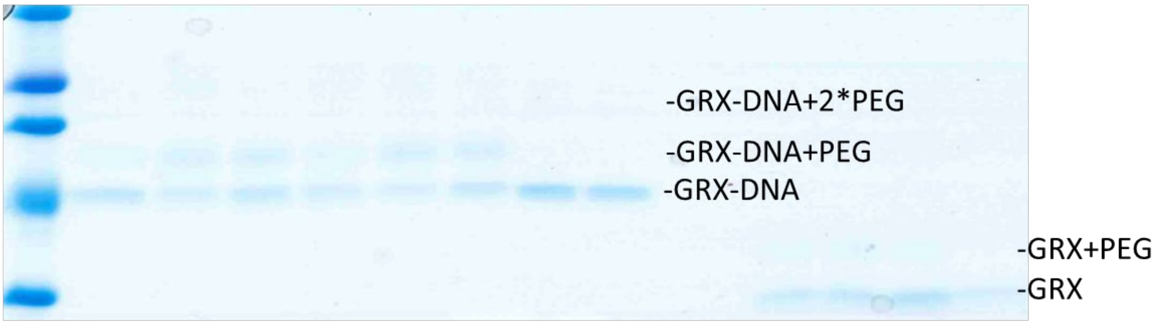

*Figure S7. GRX-DNA$_{temp}$ modified by DNA$_{cat}$-hGQ with th**3** and afterwards PEGylated with BCN-PEG$_{2000}$. Conversions were calculated using ImageJ. Slots 9-12 contain native GRX. Conditions: 20 µM GRX-DNA$_{temp}$, 22 µM DNA-hGQ, 30 µM NML **3** and 100 µM H_2O_2, pH: 7.0, at 25 °C for 30 min.*

2.4.5 Degradation of TRM-DNA_{temp}

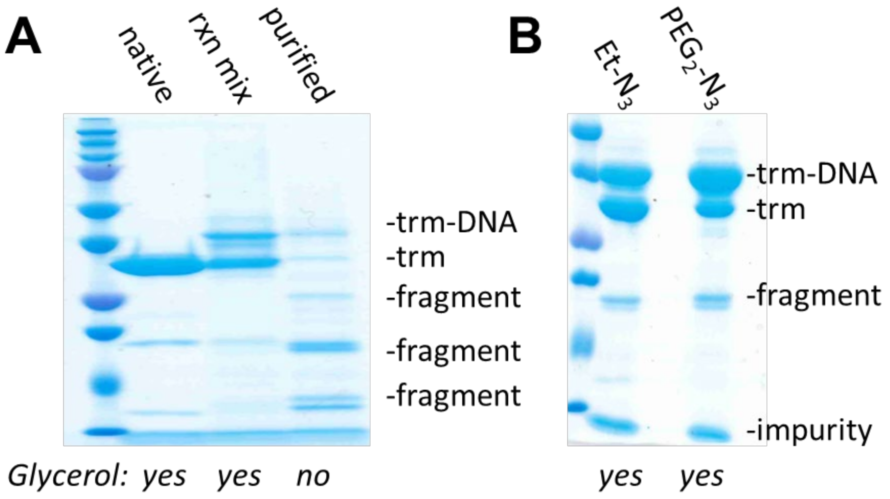

*Figure S8. (**A**) TRM-DNA_{temp} degrades after purification, which did not occur when it was still in the reaction mixture, which contained 5% glycerol. As such, (**B**) purification with spin filtration and 10% glycerol was performed and there TRM-DNA_{temp} did not degrade.*

2.4.6 CHY-Et-DNA_{temp} with DNA_{diDMAP}

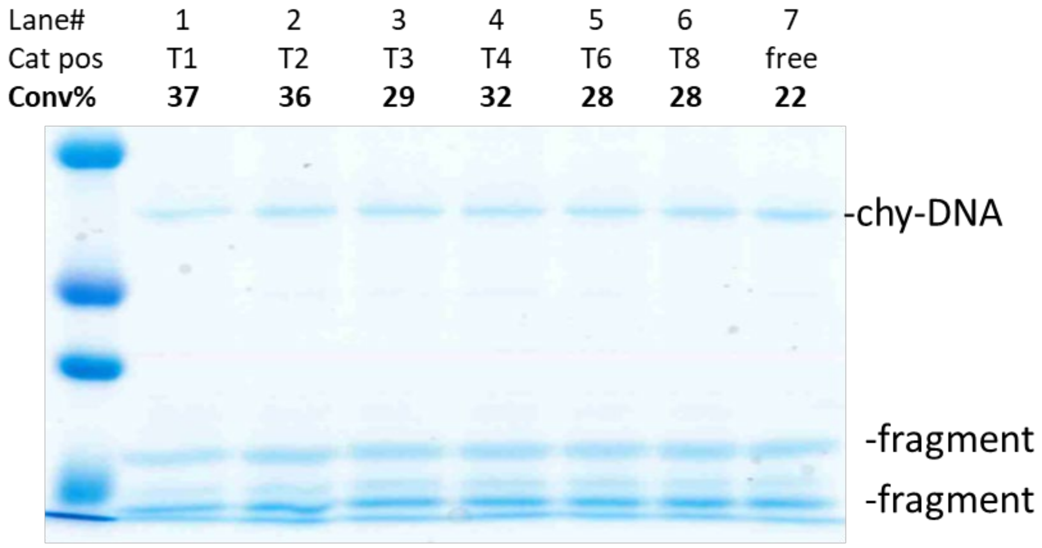

Figure S9. CHY-Et-DNA_{temp} modified by DNA_{DMAP} with thioester 1 and afterwards PEGylated with BCN-PEG_{2000}. Conversions are normalized and were calculated using ImageJ. Conditions: 20 µM CHY-Et-DNA_{temp} with 23 µM DNA_{DMAP} and 100 µM thioester 1, pH: 8.0, at 37 °C for 2 h.

2.4.7 CHY-Et-DNA$_{temp}$ with DNA$_{diPyOx}$

Lane#	1	2	3	4	5	6	7
Cat pos	T1	T2	T3	T4	T6	T8	free
**Conv%**	**95**	**91**	**82**	**75**	**62**	**22**	**0**

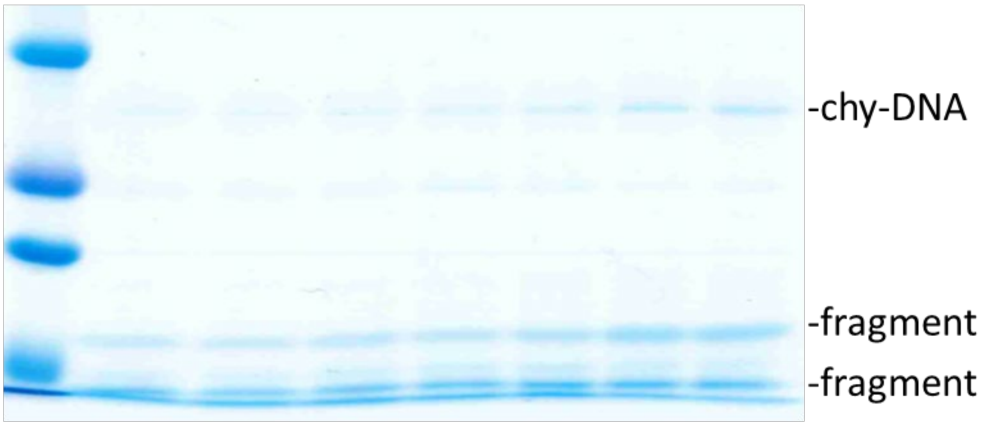

*Figure S10. CHY-EG$_2$-DNA$_{temp}$ modified by DNA$_{PyOx}$ with ANANS **2** and afterwards PEGylated with BCN-PEG(2000). Conversions are normalized and were calculated using ImageJ. Conditions: 20 µM CHY-EG$_2$-DNA$_{temp}$ with 23 µM DNA$_{PyOx}$ and 300 µM ANANS **2**, pH: 7.2, at 37 °C for 6 h.*

2.4.8 CHY-EG$_2$-DNA$_{temp}$ with DNA$_{diDMAP}$

Lane#	1	2	3	4	5	6	7
Cat pos	T1	T2	T3	T4	T6	T8	free
**Conv%**	**46**	**61**	**66**	**45**	**41**	**29**	**0**

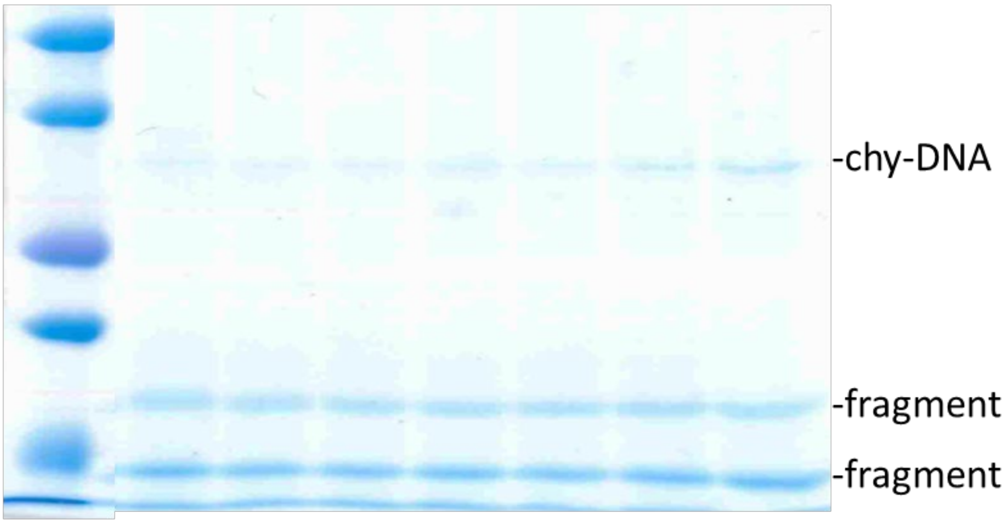

*Figure S11. CHY-EG$_2$-DNA$_{temp}$ modified by DNA$_{DMAP}$ with thioester **1** and afterwards PEGylated with BCN-PEG$_{2000}$. Conversions are normalized and were calculated using ImageJ. Conditions: 25 µM CHY-EG$_2$-DNA$_{temp}$ with 28 µM DNA$_{DMAP}$ and 100 µM thioester **1**, pH: 8.0, at 37 °C for 2 h.*

2.4.9 CHY-EG$_2$-DNA$_{temp}$ with DNA$_{diPyOx}$

Lane#	1	2	3	4	5	6	7
Cat pos	T1	T2	T3	T4	T6	T8	free
**Conv%**	**85**	**73**	**64**	**44**	**20**	**28**	**0**

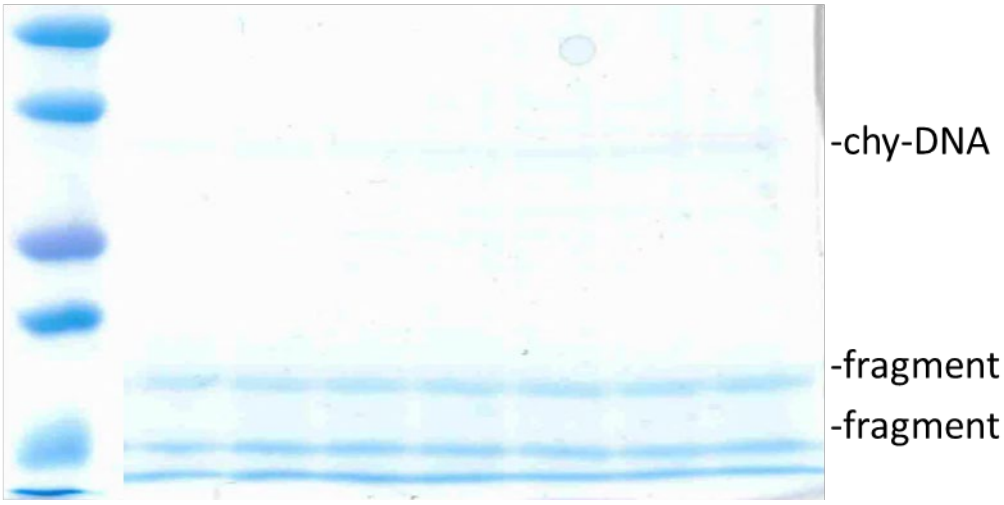

*Figure S12. CHY-EG$_2$-DNA$_{temp}$ modified by DNA$_{PyOx}$ with ANANS **2** and afterwards PEGylated with BCN-PEG(2000). Conversions are normalized and were calculated using ImageJ. Conditions: 25 µM CHY-EG$_2$-DNA$_{temp}$ with 28 µM DNA$_{PyOx}$ and 300 µM ANANS **2**, pH: 7.2, at 37 °C for 6 h.*

2.4.10 CHY with loose DNA$_{cat}$

Lane#	1	2	3	4	5	6	7
Cat	T2dP	T6dP	-	T2dD	T6dD	-	-
Tag	(2)	(2)	(2)	(1)	(1)	(1)	-
Tag (uM)	300	300	300	200	200	200	-
**Conv%**	**0**	**3**	**2**	**24**	**30**	**22**	**0**

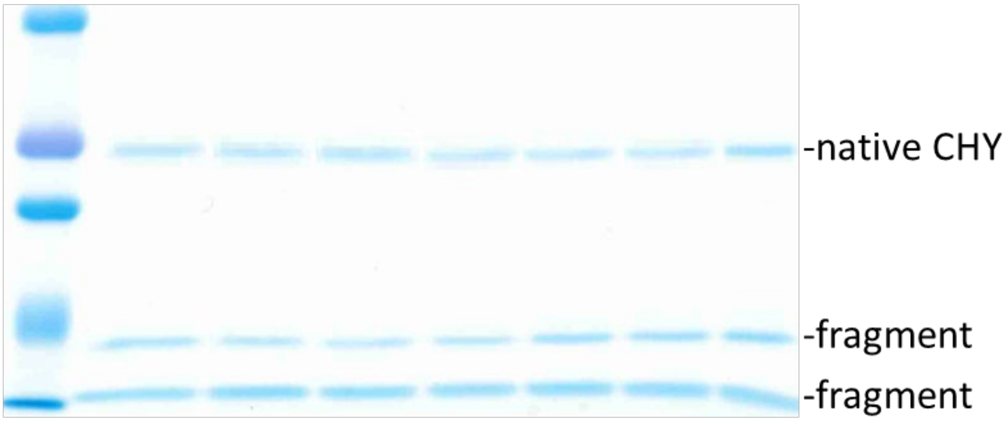

*Figure S13. Native CHY modified by unbound DNA$_{diPyOx}$ and DNA$_{diDMAP}$ with ANANS **2** and thioester **1**, respectively, and afterwards PEGylated with BCN-PEG$_{2000}$. Conversions are normalized and were calculated using ImageJ. Conditions (slot 2-4): 20 µM CHY with 30 µM DNA$_{DMAP}$ and 300 µM ANANS **2**, pH: 7.2, at 37 °C for 6 h. Conditions (slot 5-7): 20 µM CHY with 30 µM DNA$_{DMAP}$ and 200 µM thioester **1**, pH: 8.0, at 37 °C for 2 h.*

2.4.110 TRM-Et-DNA$_{temp}$ with DNA$_{diDMAP}$

Lane#	1	2	3	4	5	6	7
Cat pos	T1	T2	T3	T4	T6	T8	free
**Conv%**	**50**	**51**	**48**	**49**	**41**	**31**	**14**

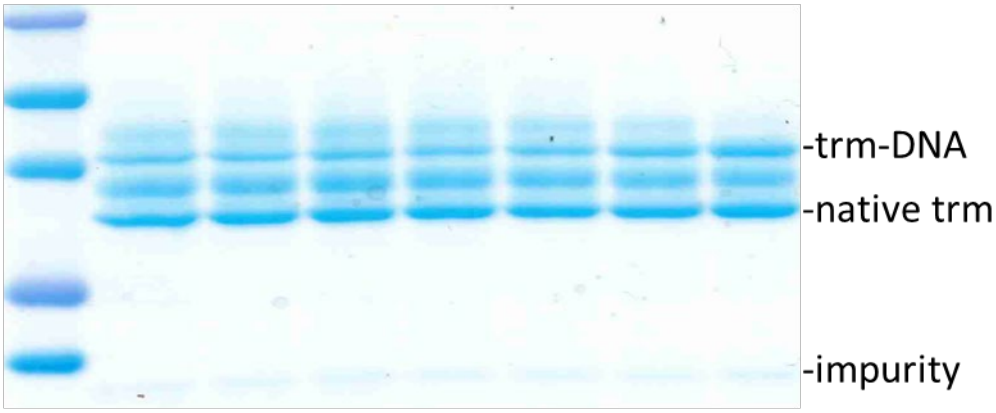

*Figure S14. TRM-Et-DNA$_{temp}$ modified by DNA$_{DMAP}$ with thioester **1** and afterwards PEGylated with BCN-PEG$_{2000}$. Conversions are normalized and were calculated using ImageJ. Conditions: 20 µM TRM-Et-DNA$_{temp}$ with 23 µM DNA$_{DMAP}$ and 100 µM thioester **1**, pH: 8.0, at 37 °C for 2 h.*

2.4.12 TRM-Et-DNA$_{temp}$ with DNA$_{diPyOx}$

Lane#	1	2	3	4	5	6	7
Cat pos	T1	T2	T3	T4	T6	T8	free
**Conv%**	**34**	**34**	**36**	**33**	**18**	**9**	**2**

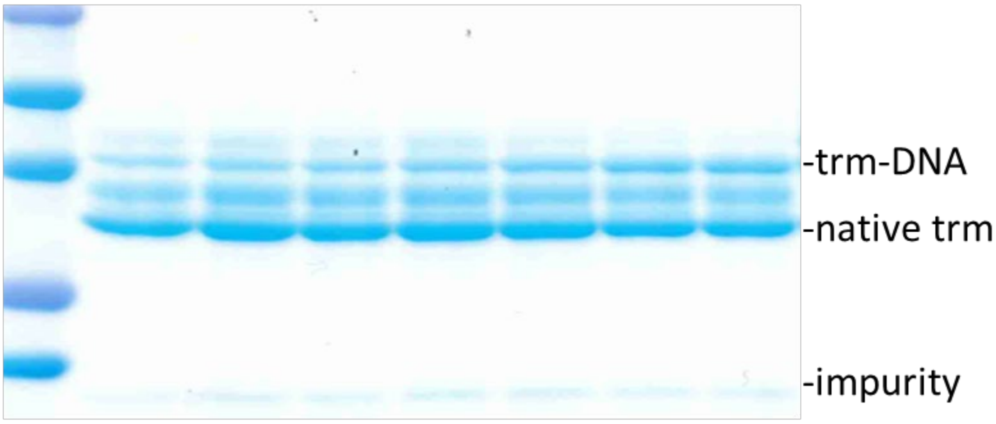

*Figure S15. TRM-Et-DNA$_{temp}$ modified by DNA$_{PyOx}$ with ANANS **2** and afterwards PEGylated with BCN-PEG(2000). Conversions are normalized and were calculated using ImageJ. Conditions: 20 µM TRM-Et-DNA$_{temp}$ with 23 µM DNA$_{PyOx}$ and 300 µM ANANS **2**, pH: 7.2, at 37 °C for 6 h.*

2.4.13 TRM-EG$_2$-DNA$_{temp}$ with DNA$_{diDMAP}$

Lane#	1	2	3	4	5	6	7
Cat pos	T1	T2	T3	T4	T6	T8	free
**Conv%**	**57**	**53**	**62**	**56**	**50**	**31**	**11**

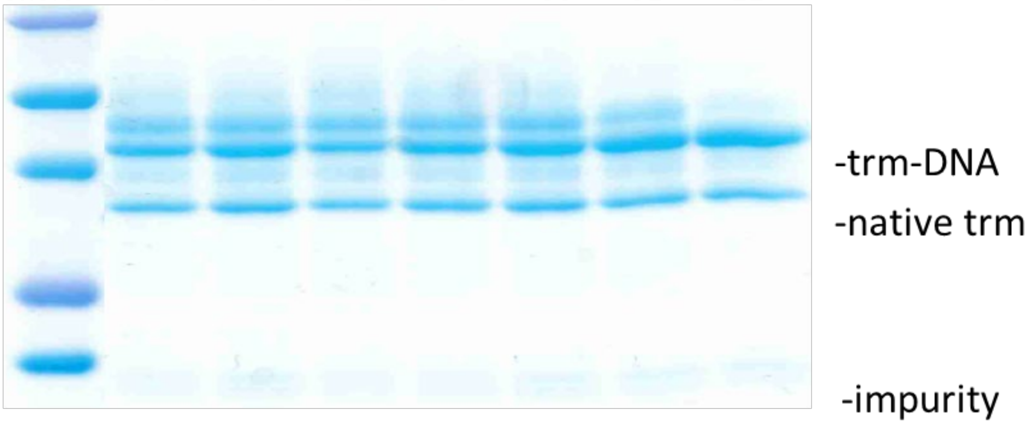

*Figure S16. TRM-EG$_2$-DNA$_{temp}$ modified by DNA$_{DMAP}$ with thioester **1** and afterwards PEGylated with BCN-PEG$_{2000}$. Conversions are normalized and were calculated using ImageJ. Conditions: 26 µM TRM-EG$_2$-DNA$_{temp}$ with 28 µM DNA$_{DMAP}$ and 100 µM thioester **1**, pH: 8.0, at 37 °C for 2 h.*

2.4.14 TRM-EG$_2$-DNA$_{temp}$ with DNA$_{diPyOx}$

Lane#	1	2	3	4	5	6	7
Cat pos	T1	T2	T3	T4	T6	T8	free
**Conv%**	**55**	**58**	**47**	**51**	**34**	**13**	**2**

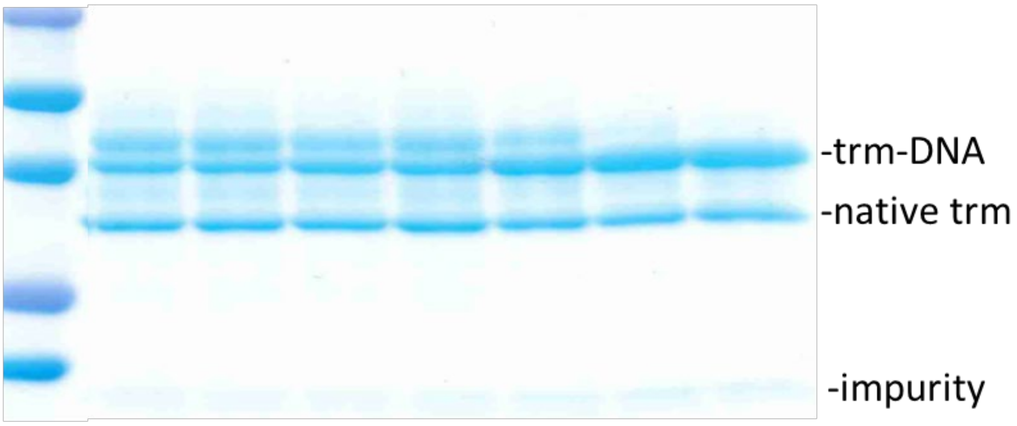

*Figure S17. TRM-EG$_2$-DNA$_{temp}$ modified by DNA$_{PyOx}$ with ANANS **2** and afterwards PEGylated with BCN-PEG(2000). Conversions are normalized and were calculated using ImageJ. Conditions: 26 µM TRM-EG$_2$-DNA$_{temp}$ with 28 µM DNA$_{PyOx}$ and 300 µM ANANS **2**, pH: 7.2, at 37 °C for 6 h.*

2.4.15 TRM-Et-DNA_{temp} with DNA_{cat}-hGQ DNAzyme

Lane#	1	2	3	4	5	6	7	8	9
[NML] (uM)	66	66	66	66	133	133	133	133	-
Cat pos	T0	T10	T20	PW17	T0	T10	T20	PW17	-
**Conv%**	**40**	**41**	**26**	**34**	**56**	**63**	**55**	**62**	-

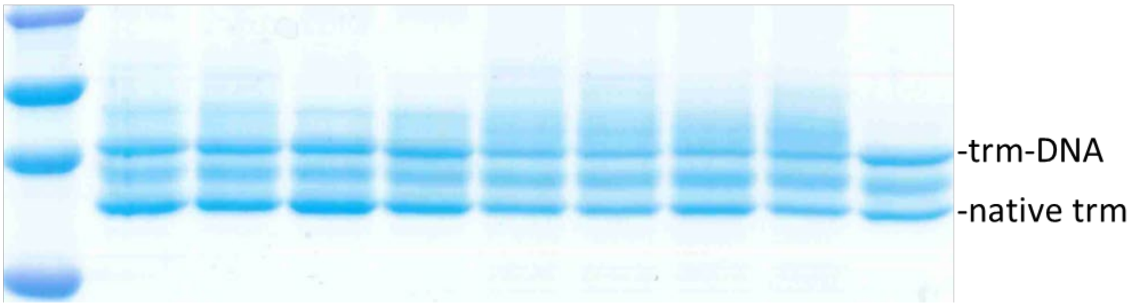

*Figure S18. TRM-Et-DNA_{temp} modified by DNA-hGQ DNAzyme with NML **3** and afterwards PEGylated with BCN-PEG_{2000}. Conversions were calculated using ImageJ. Conditions: 20 µM TRM-Et-DNA_{temp}, 30 µM DNA_{cat}-hGQ and [NML **3**] = [H_2O_2] = 66 or 133 µM, pH: 7.0, at 25 °C for 30 min.*

2.4.16 TRM-EG_2-DNA_{temp} with DNA_{cat}-hGQ DNAzyme

Lane#	1	2	3	4	5	6	7	8	9
[NML] (uM)	66	66	66	66	133	133	133	133	-
Cat pos	T0	T10	T20	PW17	T0	T10	T20	PW17	-
**Conv%**	**36**	**36**	**26**	**33**	**53**	**52**	**59**	**58**	-

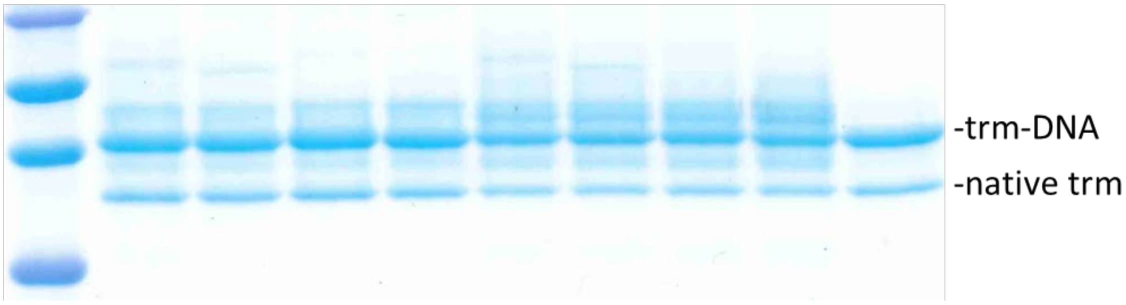

*Figure S19. TRM-EG_2-DNA_{temp} modified by DNA-hGQ DNAzyme with NML **3** and afterwards PEGylated with BCN-PEG_{2000}. Conversions were calculated using ImageJ. Conditions: 26 µM TRM-EG_2-DNA_{temp}, 30 µM DNA-hGQ and [NML **3**] = [H_2O_2] = 66 or 133 µM, pH: 7.0, at 25 °C for 30 min.*

2.4.17 TRM with loose DNA_{cat}

Lane#	1	2	3	4	5	6	7
Cat	-	T2dP	T6dP	-	T2dD	T6dD	-
Tag	-	(2)	(2)	(2)	(1)	(1)	(1)
Tag (uM)	-	300	300	300	150	150	150
**Conv%**	-	**3**	**2**	**2**	**6**	**5**	**5**

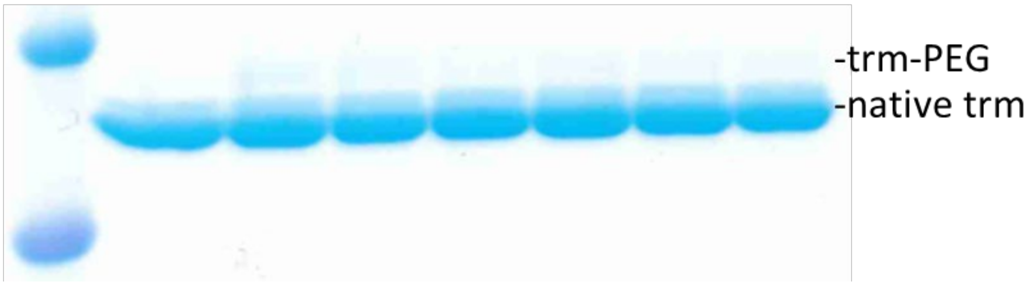

*Figure S20. Native TRM modified by unbound DNA_{diPyOx} and DNA_{diDMAP} with ANANS **2** and thioester **1**, respectively, and afterwards PEGylated with BCN-PEG_{2000}. Conversions were calculated using ImageJ. Conditions (slot 2-4): 20 µM TRM with 30 µM DNA_{DMAP} and 300 µM ANANS **2**, pH: 7.2, at 37 °C for 6 h. Conditions (slot 5-7): 20 µM TRM with 30 µM DNA_{DMAP} and 150 µM thioester **1**, pH: 8.0, at 37 °C for 2 h.*

2.5 HPLC and MS data

2.5.1 MS data of protein-N_3 and GRX-DNA_{temp}

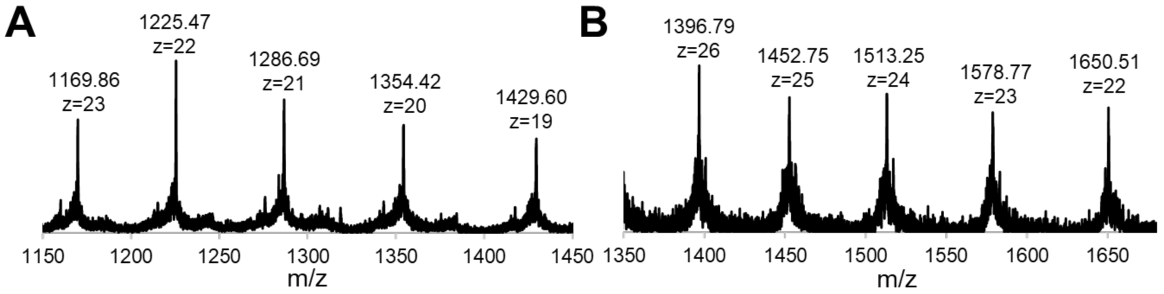

*Figure S21. (**A**) Mass spectrometry data of CHY-EG$_2$-N$_3$. Calculated peaks: 1169.8(z=22), 1225.4(z=21), 1286.7(z=20), 1354.3(z=19), 1429.5(z=18). (**B**) Mass spectrometry data of TRM-EG$_2$-N$_3$. Calculated peaks: 1396.8 (z=26), 1452.7(z=25), 1513.1(z=24), 1578.9(z=23), 1650.6(z=22).*

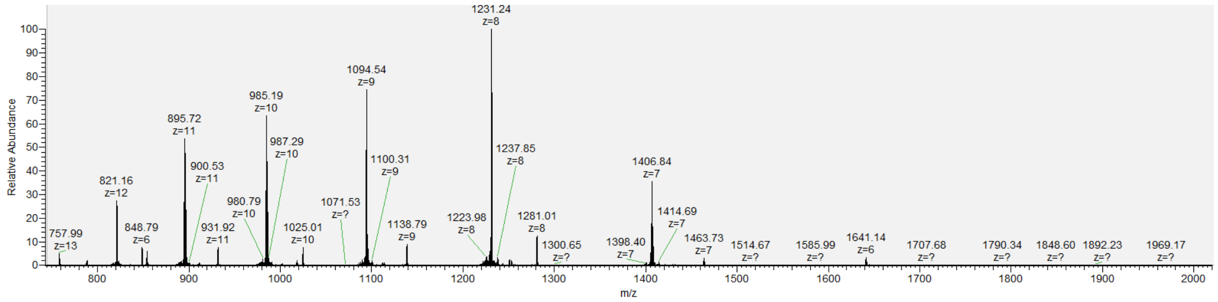

Figure S22. Mass spectrometry data of GRX-N$_3$. Total mass = 9840 Da.

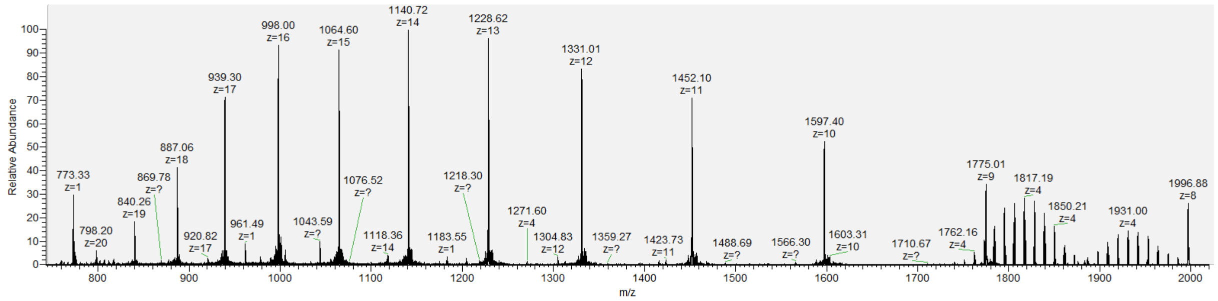

Figure S23. Mass spectrometry data of GRX-DNA$_{temp}$. Total mass = 15835 Da.

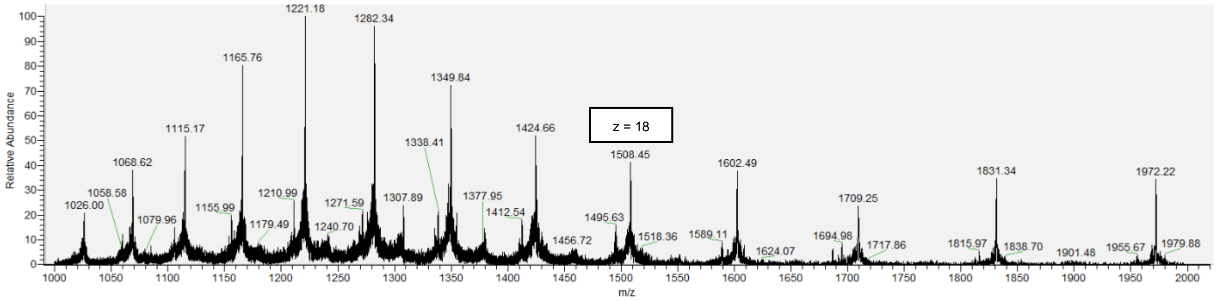

Figure S24. Mass spectrometry data of CHY-Et-N$_3$. Total mass = 25625 Da.

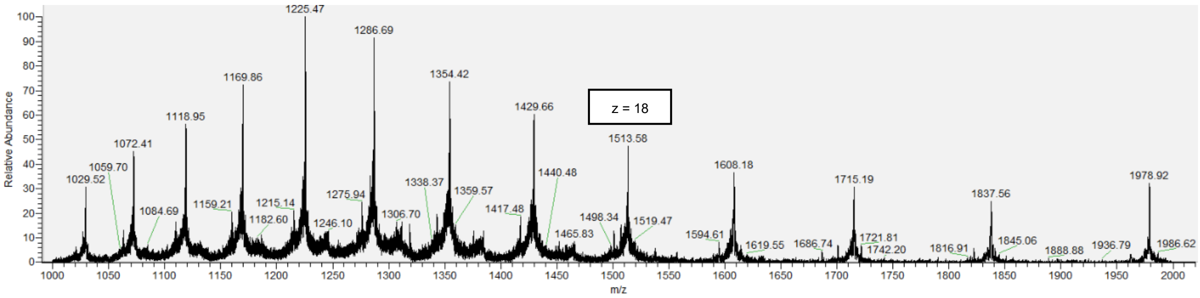

Figure S25. Mass spectrometry data of CHY-EG$_2$-N$_3$.Total mass = 25713 Da.

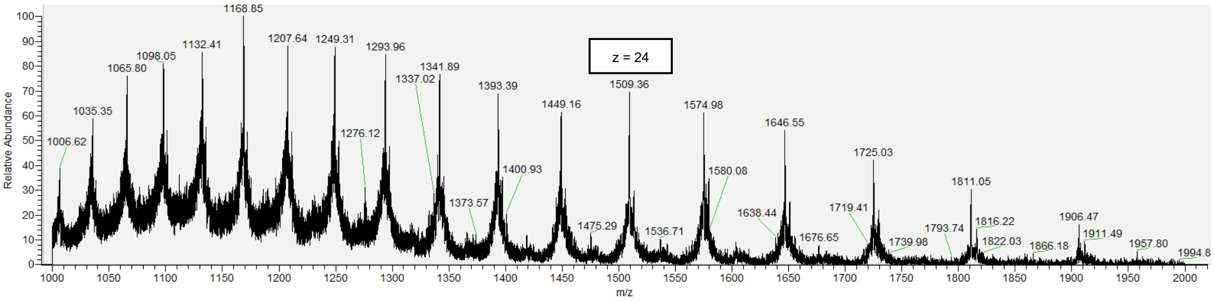

Figure S26. Mass spectrometry data of TRM-Et-N$_3$. Total mass = 36203 Da.

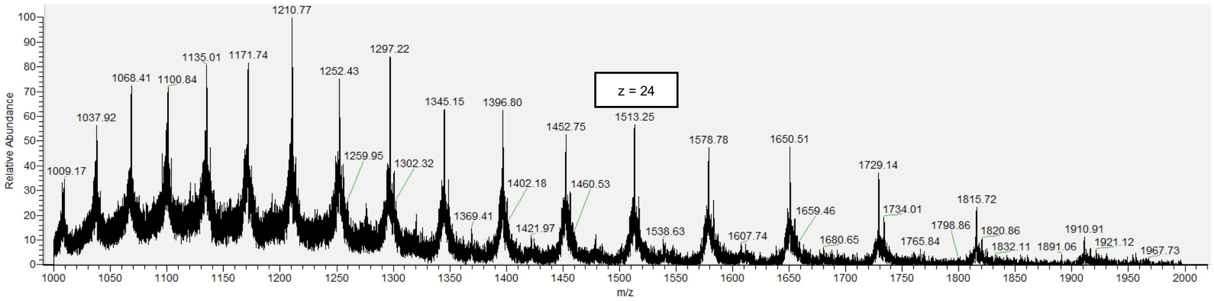

Figure S27. Mass spectrometry data of TRM-EG$_2$-N$_3$. Total mass = 36291 Da.

2.5.2 HPLC and MS data of DNA strands

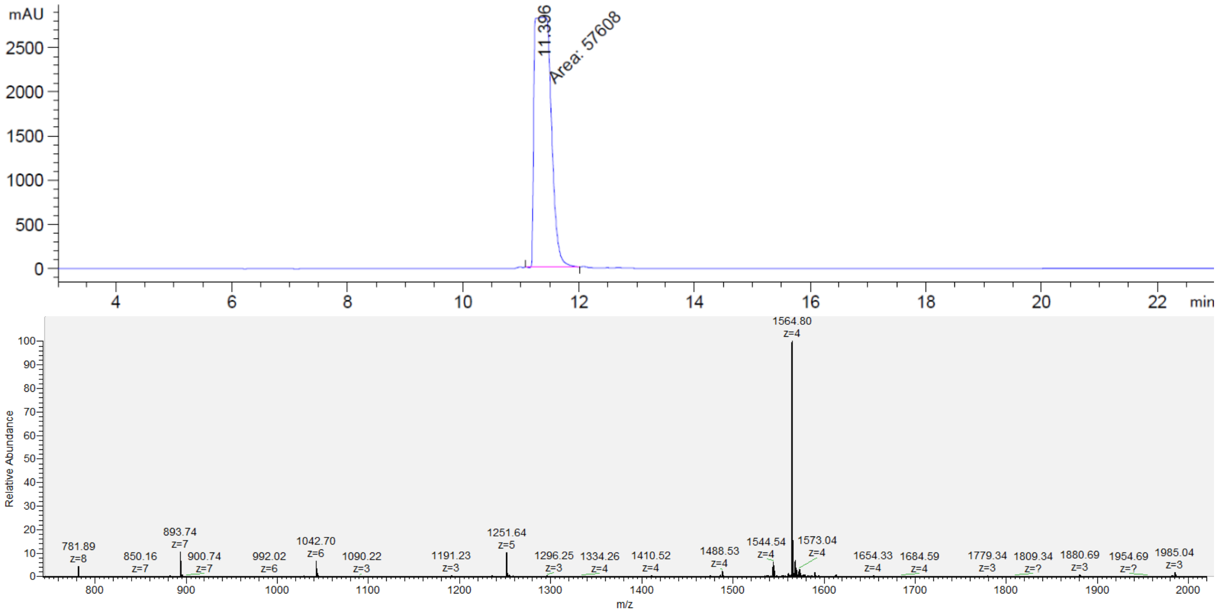

Figure S28. DNA$_{catalyst}$-alkyne HPLC trace (Abs 260 nm) and mass spectrometry data. Calculated mass(z) = 781.9(8), 893.7(7), 1042.7(6), 1251.6(5), 1564.8(4).

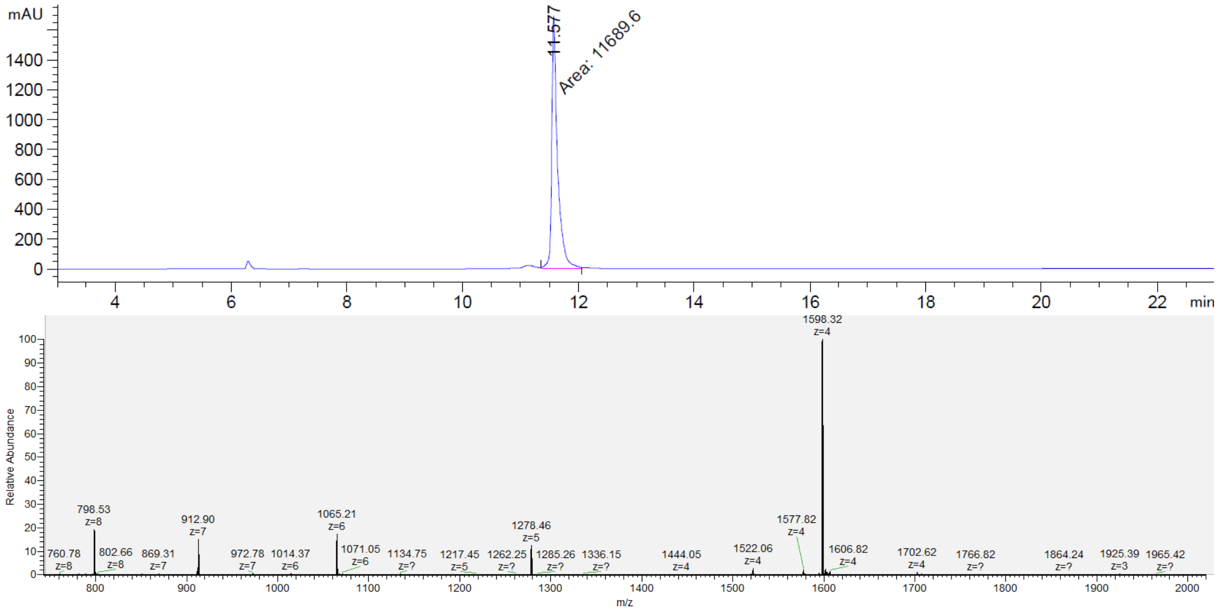

Figure S29. DNA$_{DMAP}$[1] HPLC trace (Abs 260 nm) and mass spectrometry data. Calculated mass(z) = 798.5(8), 912.9(7), 1065.2(6), 1278.4(5), 1598.3(4).

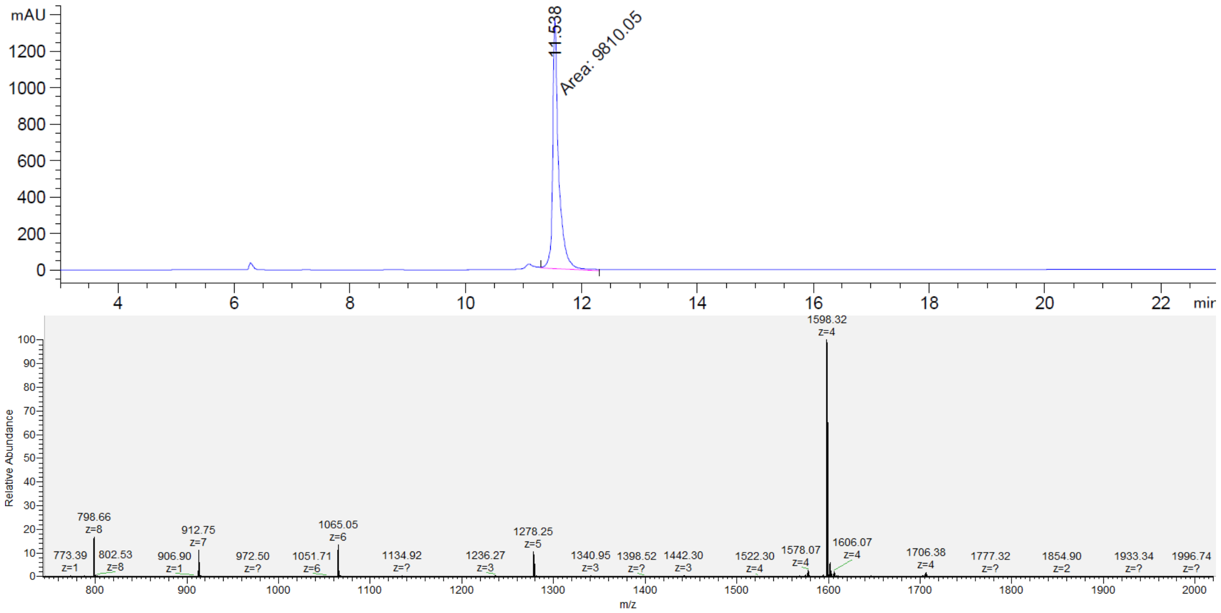

Figure S30. DNA$_{DMAP}$^{2} HPLC trace (Abs 260 nm) and mass spectrometry data. Calculated mass(z) = 798.5(8), 912.9(7), 1065.2(6), 1278.4(5), 1598.3(4).

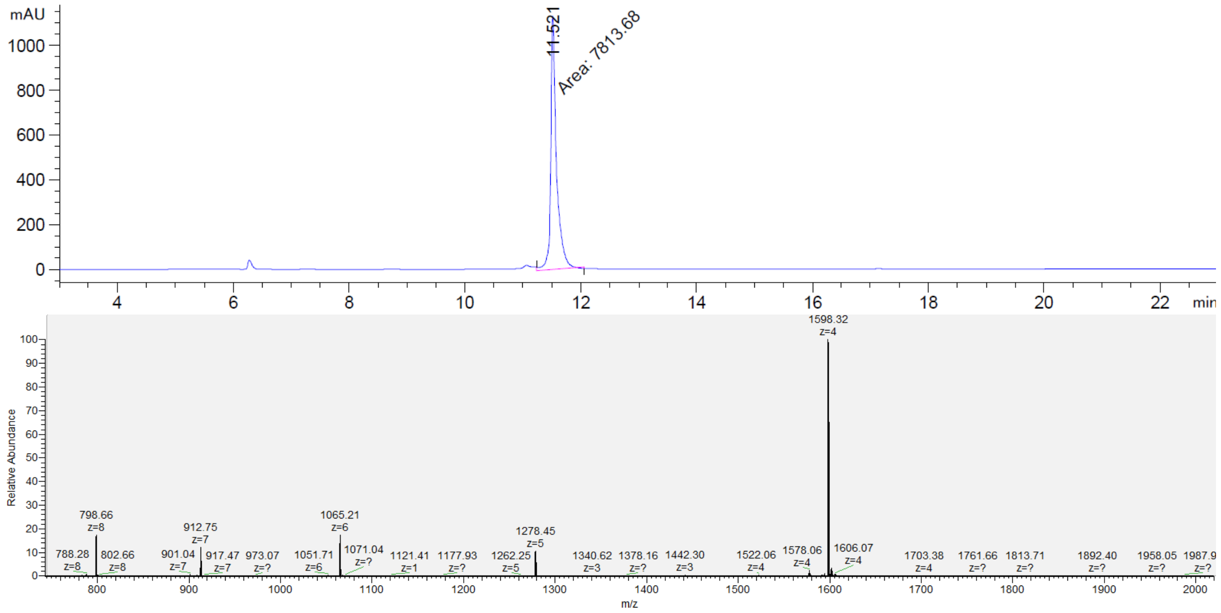

Figure S31. DNA$_{DMAP}$^{3} HPLC trace (Abs 260 nm) and mass spectrometry data. Calculated mass(z) = 798.5(8), 912.9(7), 1065.2(6), 1278.4(5), 1598.3(4).

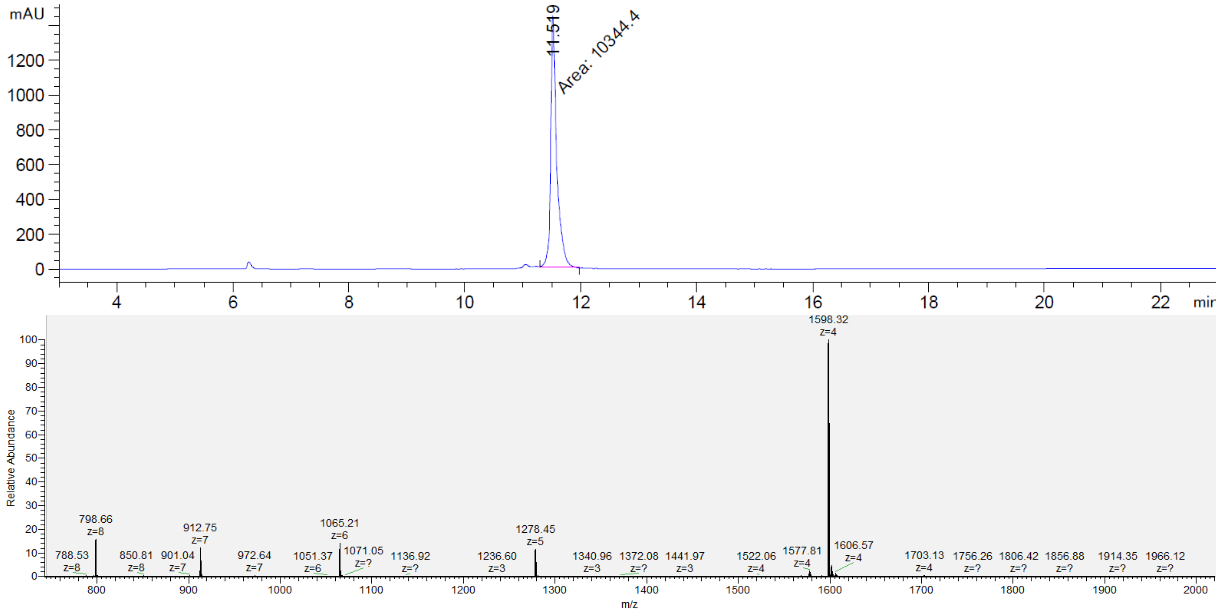

Figure S32. DNA$_{DMAP}$^{4} HPLC trace (Abs 260 nm) and mass spectrometry data. Calculated mass(z) = 798.5(8), 912.9(7), 1065.2(6), 1278.4(5), 1598.3(4).

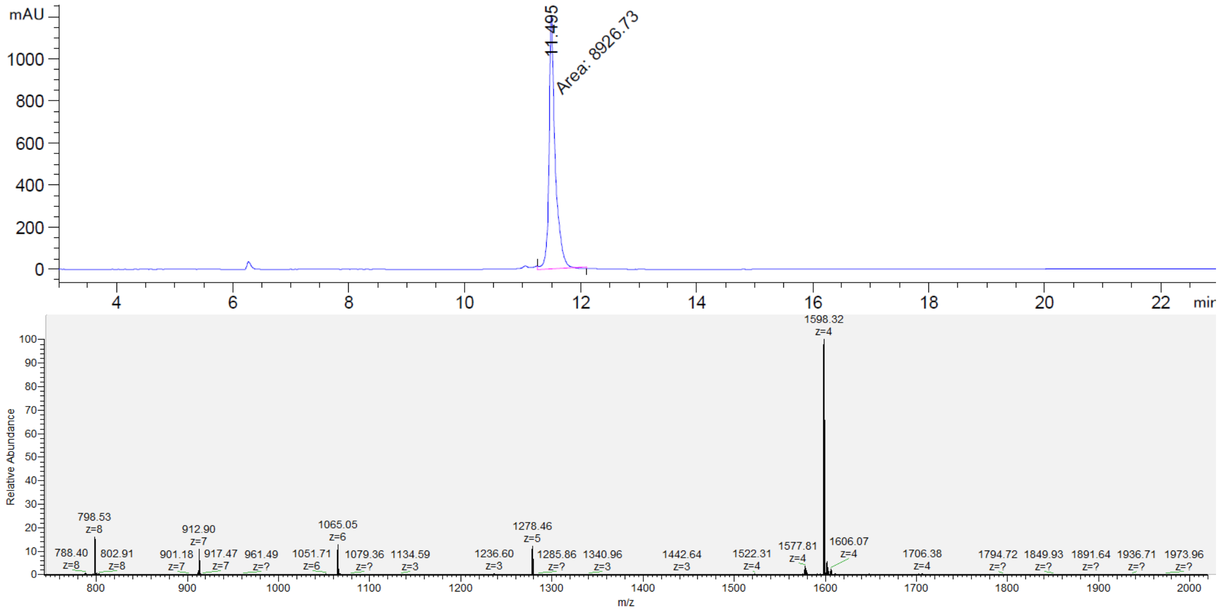

Figure S33. DNA$_{DMAP}$^{6} HPLC trace (Abs 260 nm) and mass spectrometry data. Calculated mass(z) = 798.5(8), 912.9(7), 1065.2(6), 1278.4(5), 1598.3(4).

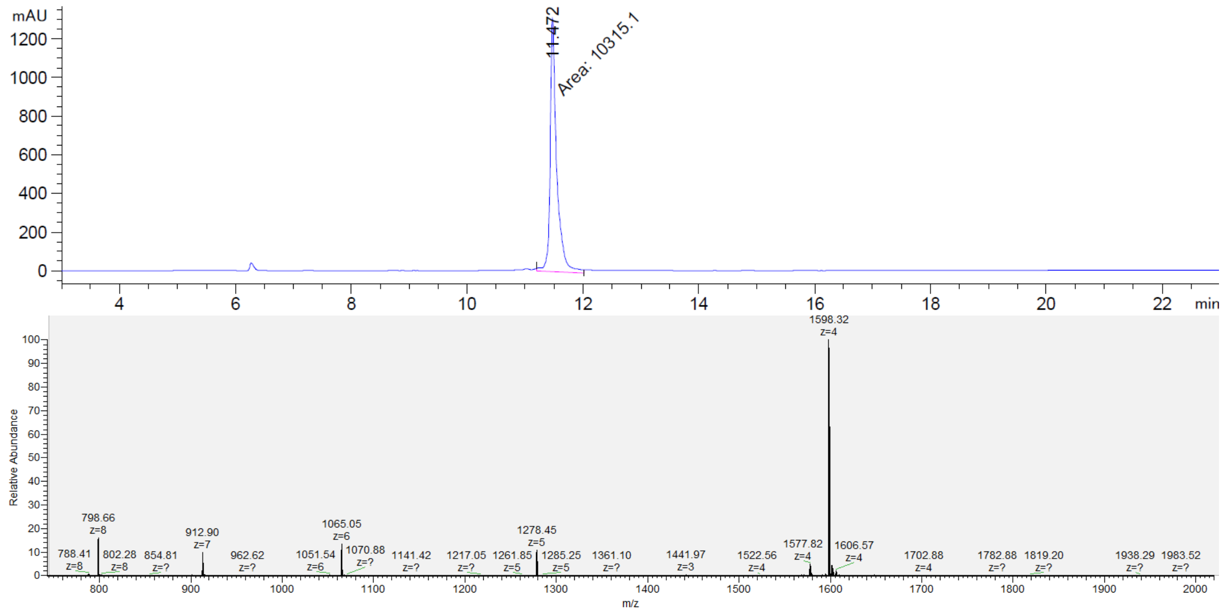

Figure S34. DNA_{DMAP}^{8} HPLC trace (Abs 260 nm) and mass spectrometry data. Calculated mass(z) = 798.5(8), 912.9(7), 1065.2(6), 1278.4(5), 1598.3(4).

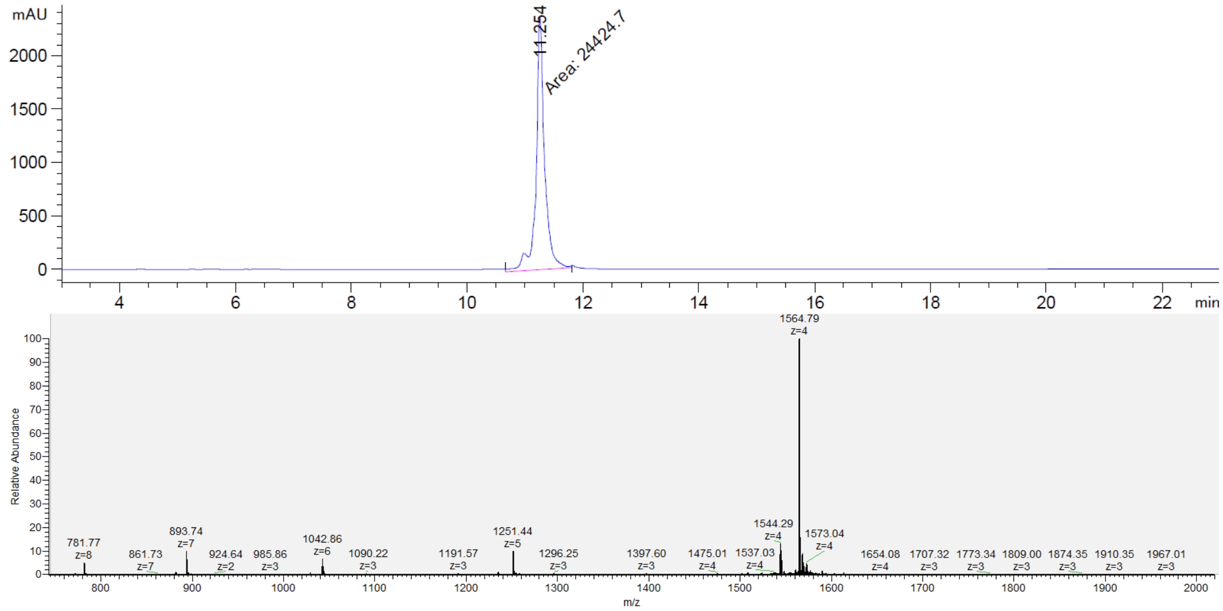

Figure S35. DNA_{PyOx}^{1} HPLC trace (Abs 260 nm) and mass spectrometry data. Calculated mass(z) = 781.9(8), 893.7(7), 1042.7(6), 1251.6(5), 1564.8(4).

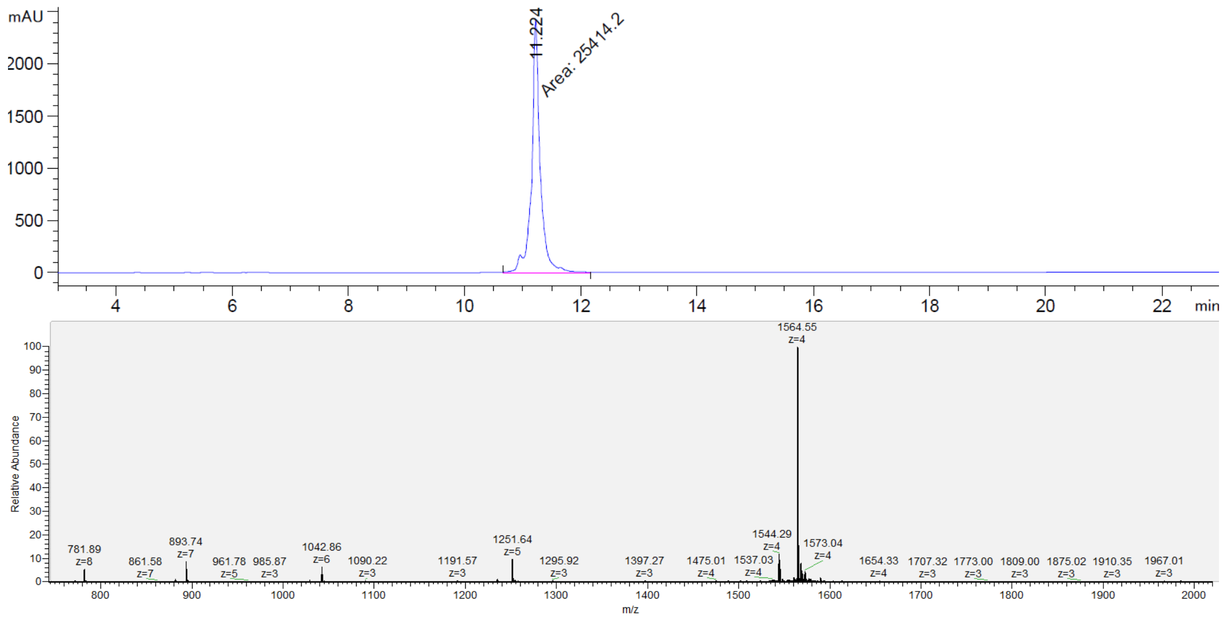

Figure S36. DNA_{PyOx}^{2} HPLC trace (Abs 260 nm) and mass spectrometry data. Calculated mass(z) = 781.9(8), 893.7(7), 1042.7(6), 1251.6(5), 1564.8(4).

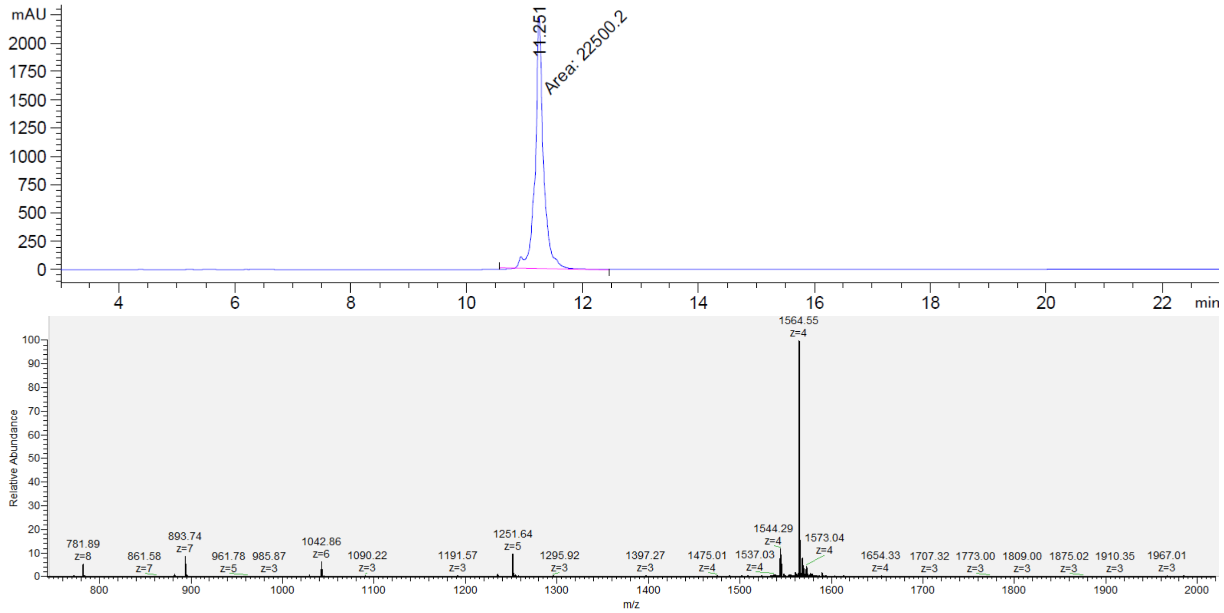

Figure S37. DNA_{PyOx}^{3} HPLC trace (Abs 260 nm) and mass spectrometry data. Calculated mass(z) = 781.9(8), 893.7(7), 1042.7(6), 1251.6(5), 1564.8(4).

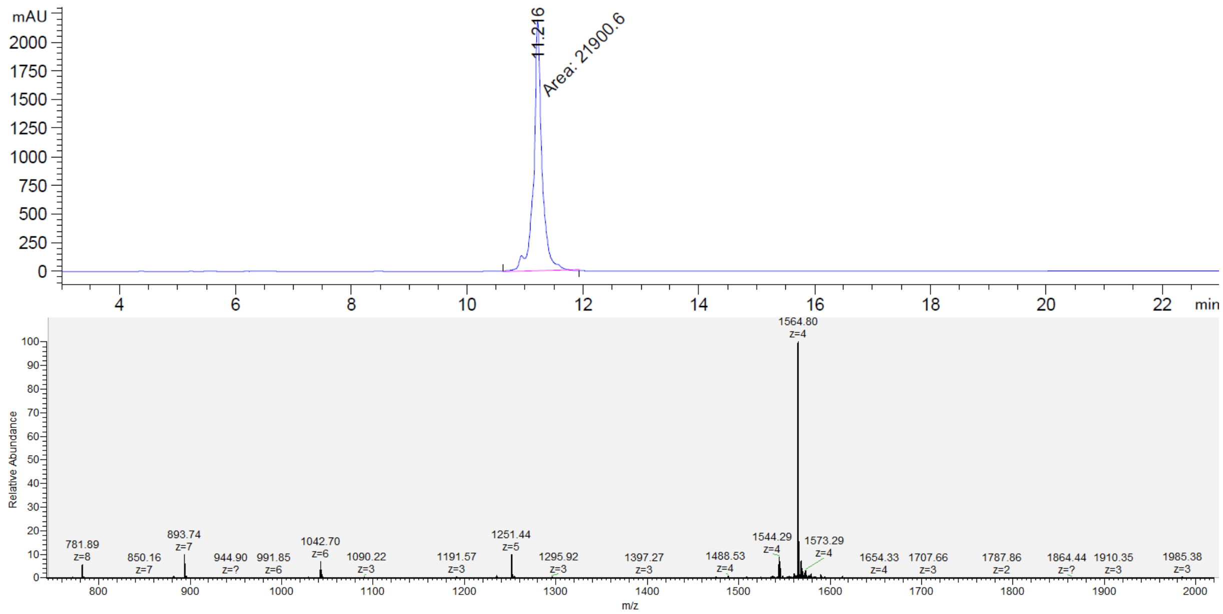

Figure S38. DNA$_{PyOx}$^{4} HPLC trace (Abs 260 nm) and mass spectrometry data. Calculated mass(z) = 781.9(8), 893.7(7), 1042.7(6), 1251.6(5), 1564.8(4).

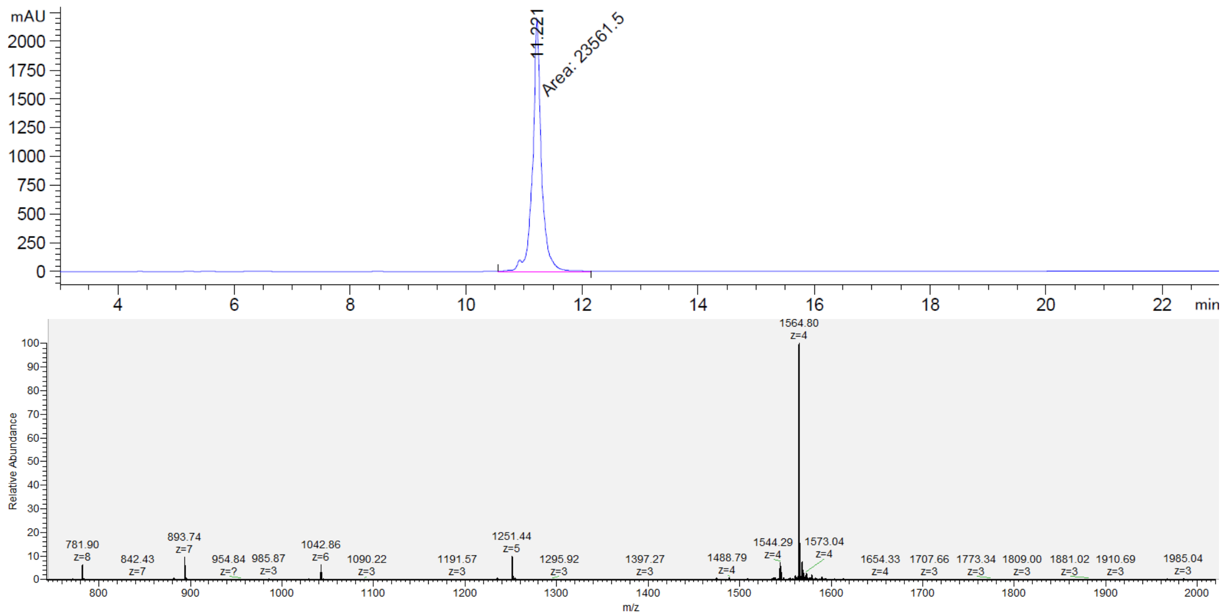

Figure S39. DNA$_{PyOx}$^{6} HPLC trace (Abs 260 nm) and mass spectrometry data. Calculated mass(z) = 781.9(8), 893.7(7), 1042.7(6), 1251.6(5), 1564.8(4).

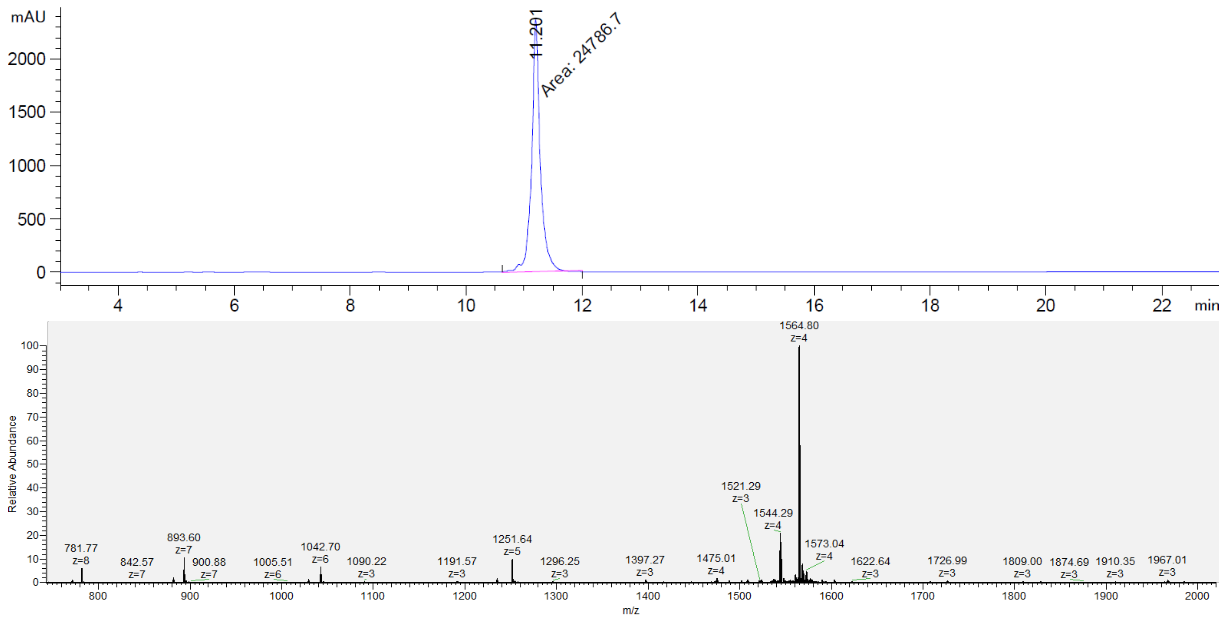

Figure S40. DNA$_{PyOx}$^{8} HPLC trace (Abs 260 nm) and mass spectrometry data. Calculated mass(z) = 781.9(8), 893.7(7), 1042.7(6), 1251.6(5), 1564.8(4).

2.6 NMR data of organic compounds

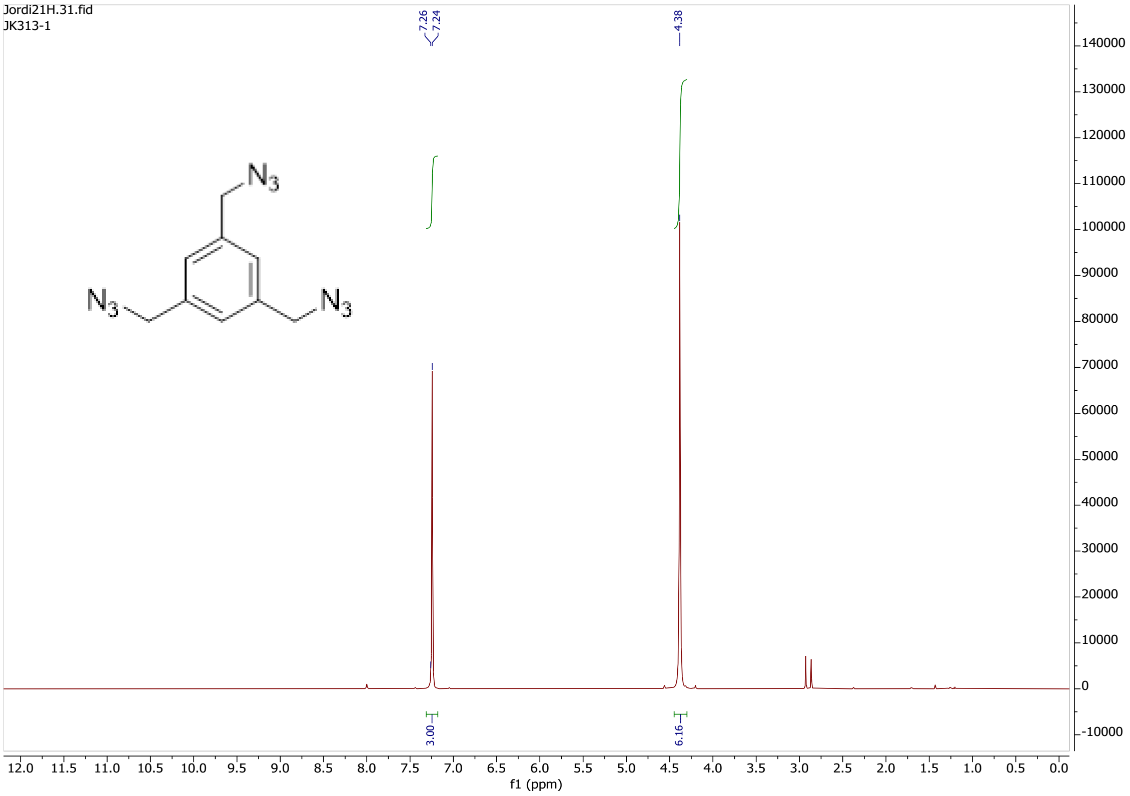

Figure S41. ¹H NMR spectrum of 1,3,5-tris(azidomethyl)benzene.

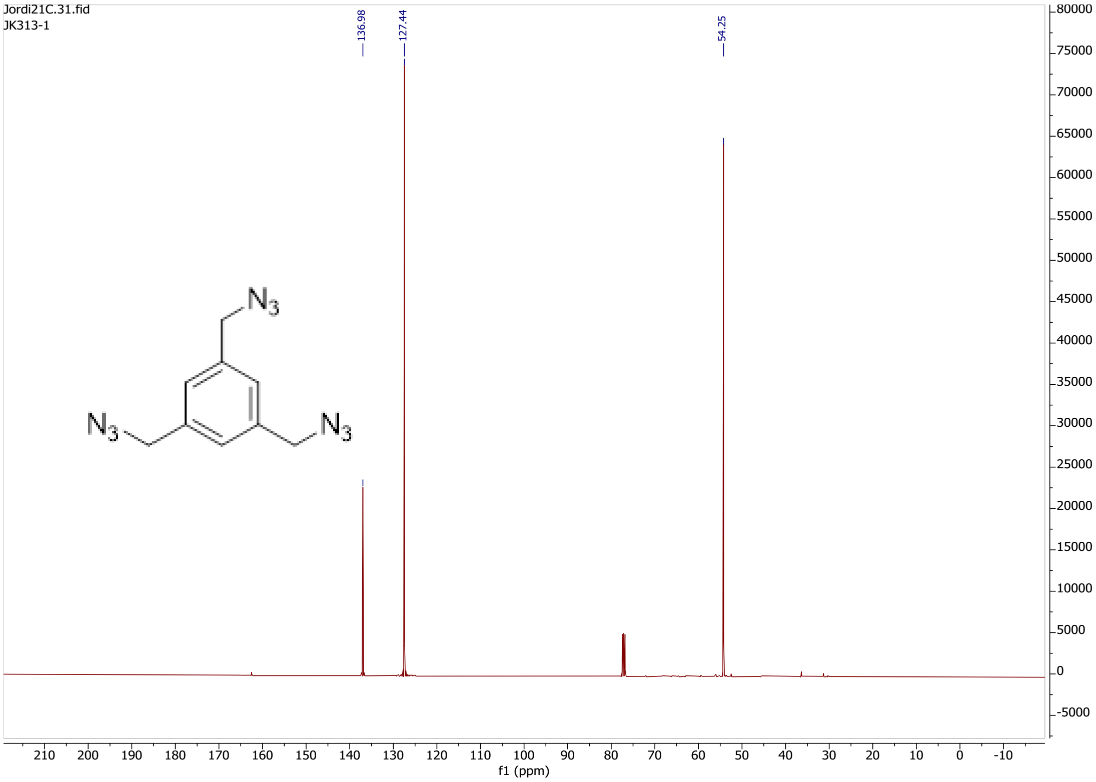

Figure S42. ¹³C NMR spectrum of 1,3,5-tris(azidomethyl)benzene.

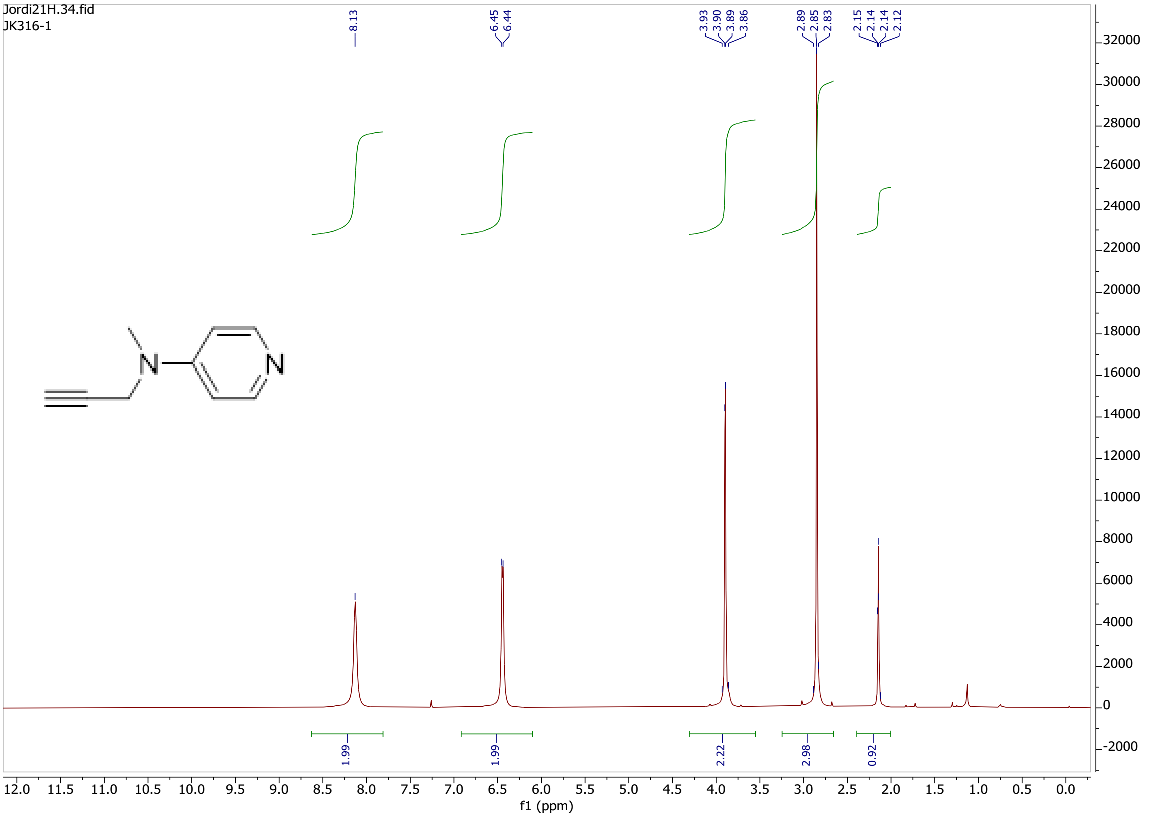

Figure S43. ^1H NMR spectrum of alkyne-DMAP.

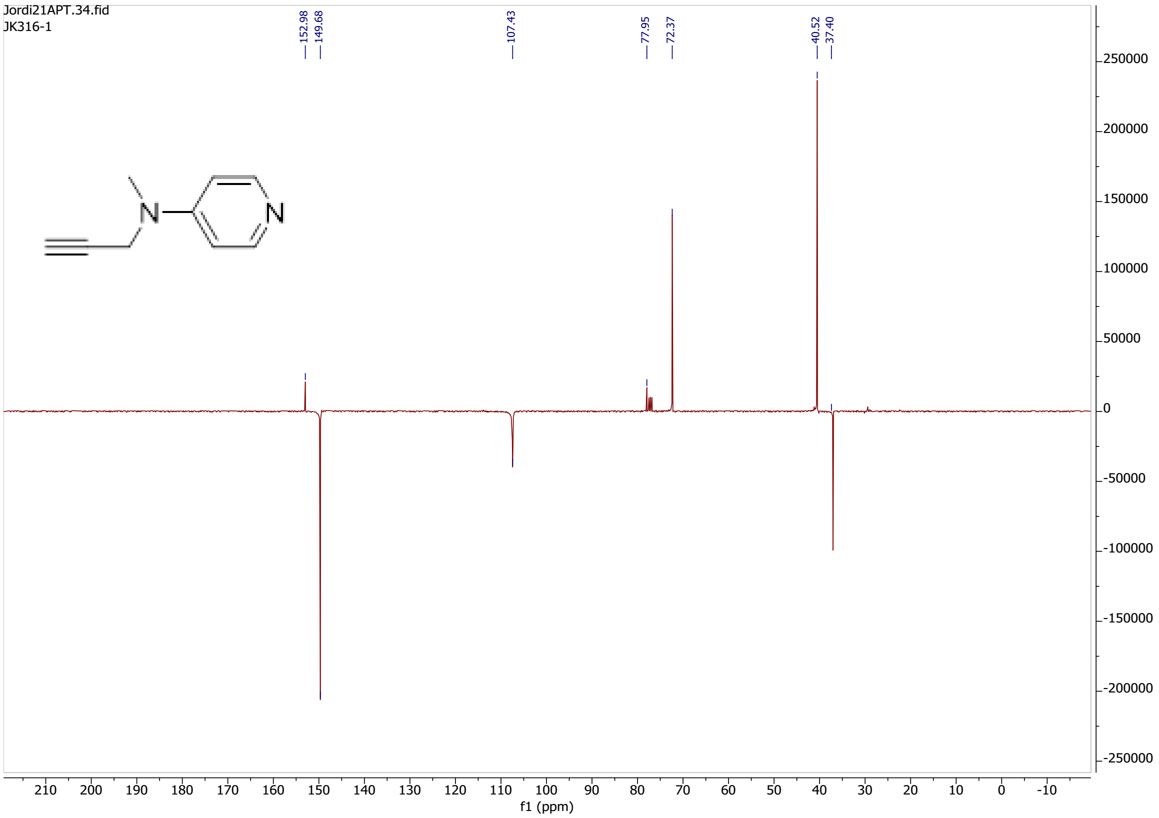

Figure S44. ^{13}C NMR spectrum of alkyne-DMAP.

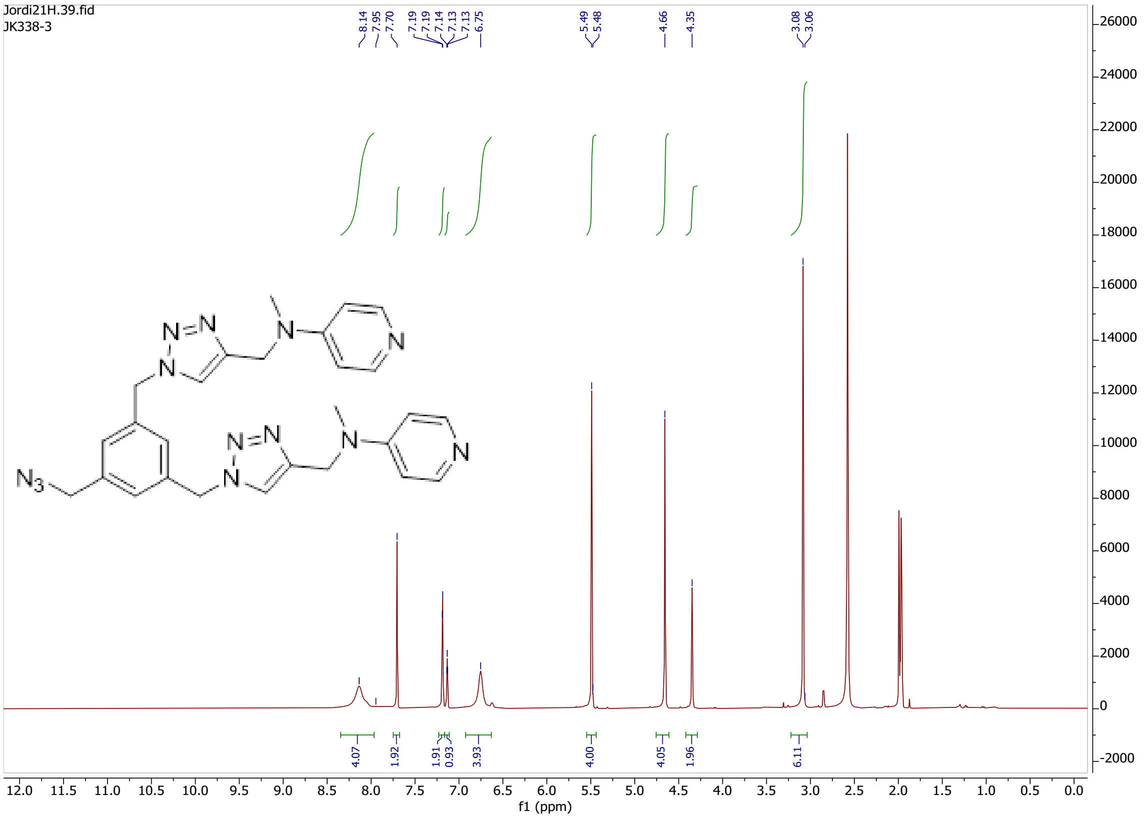

Figure S45. ^{1}H NMR spectrum of azido-diDMAP.

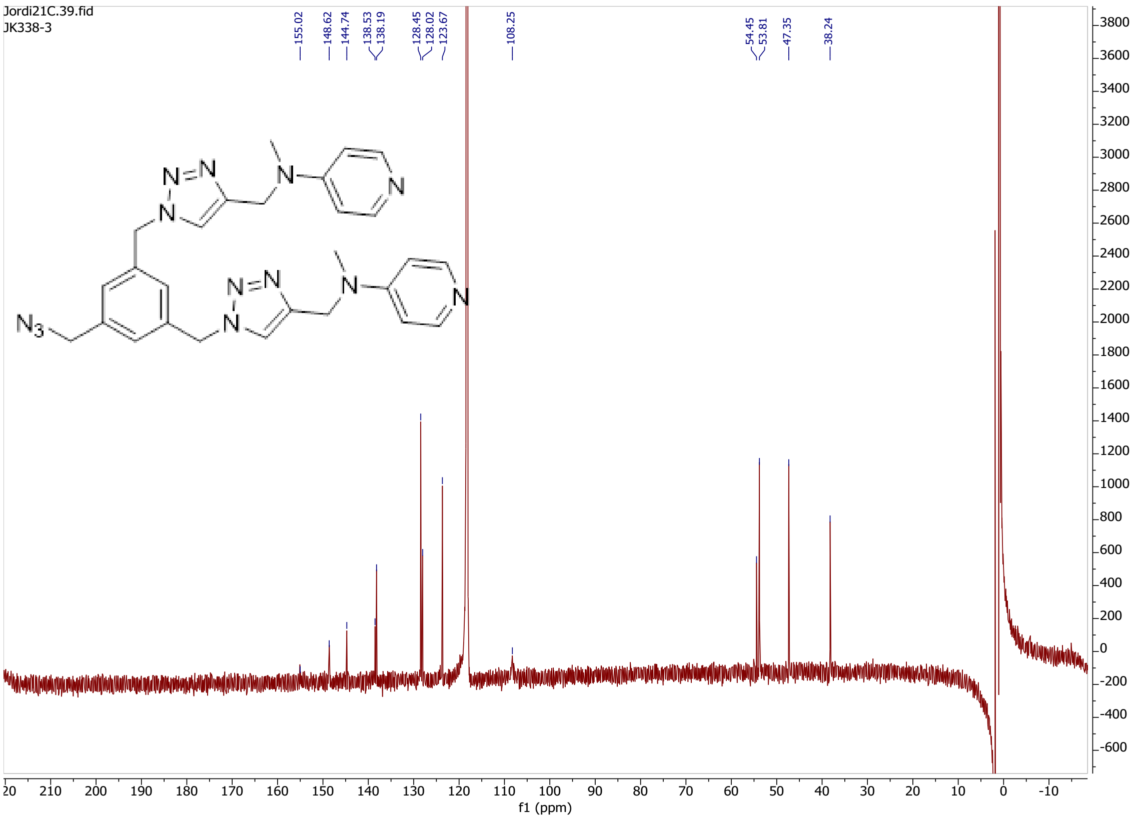

Figure S46. ^{13}C NMR spectrum of azido-diDMAP.

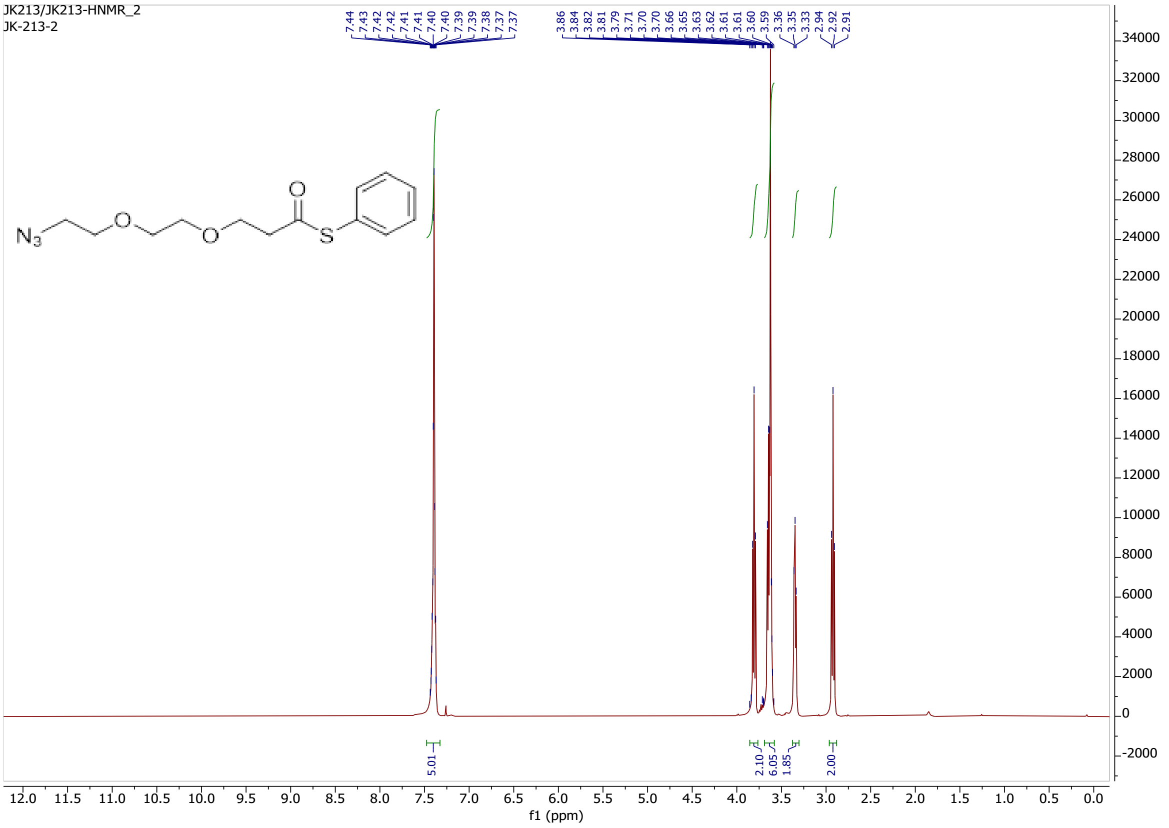

*Figure S47. ^{1}H NMR spectrum of azido-thioester **1.***

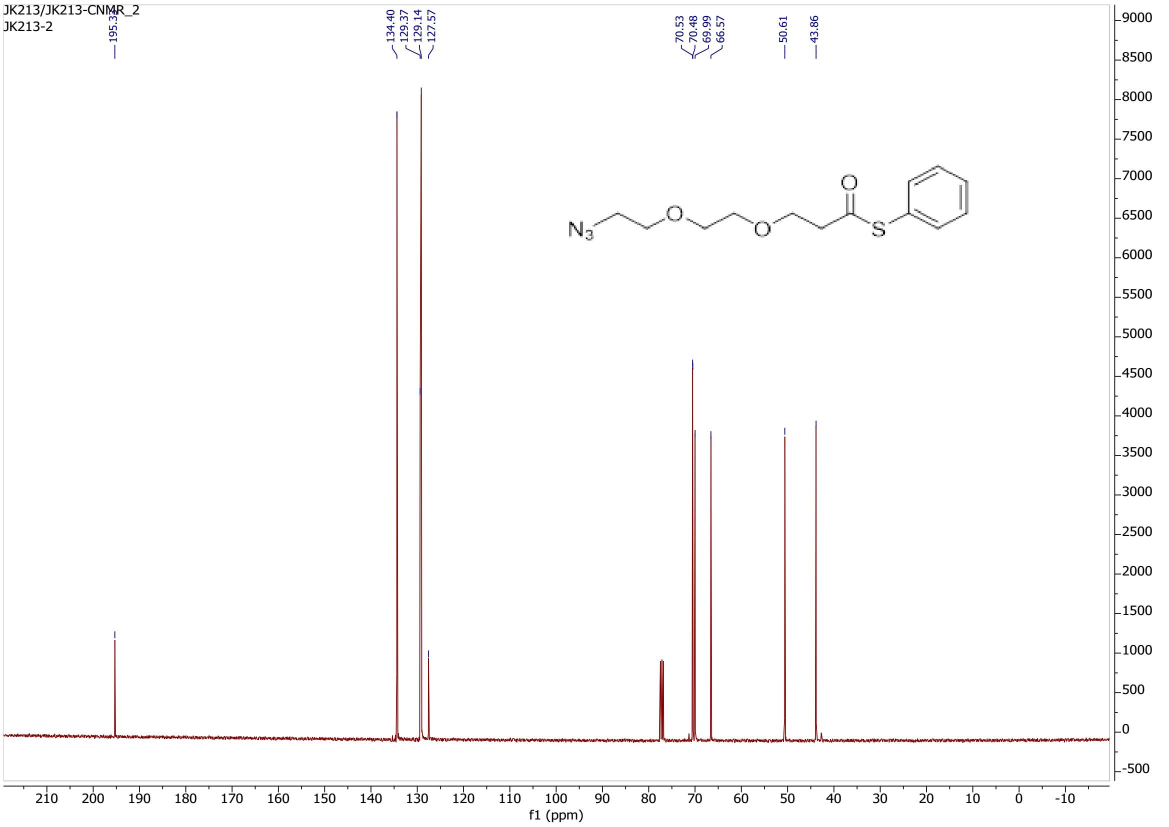

*Figure S48. ^{13}C NMR spectrum of azido-thioester **1.***

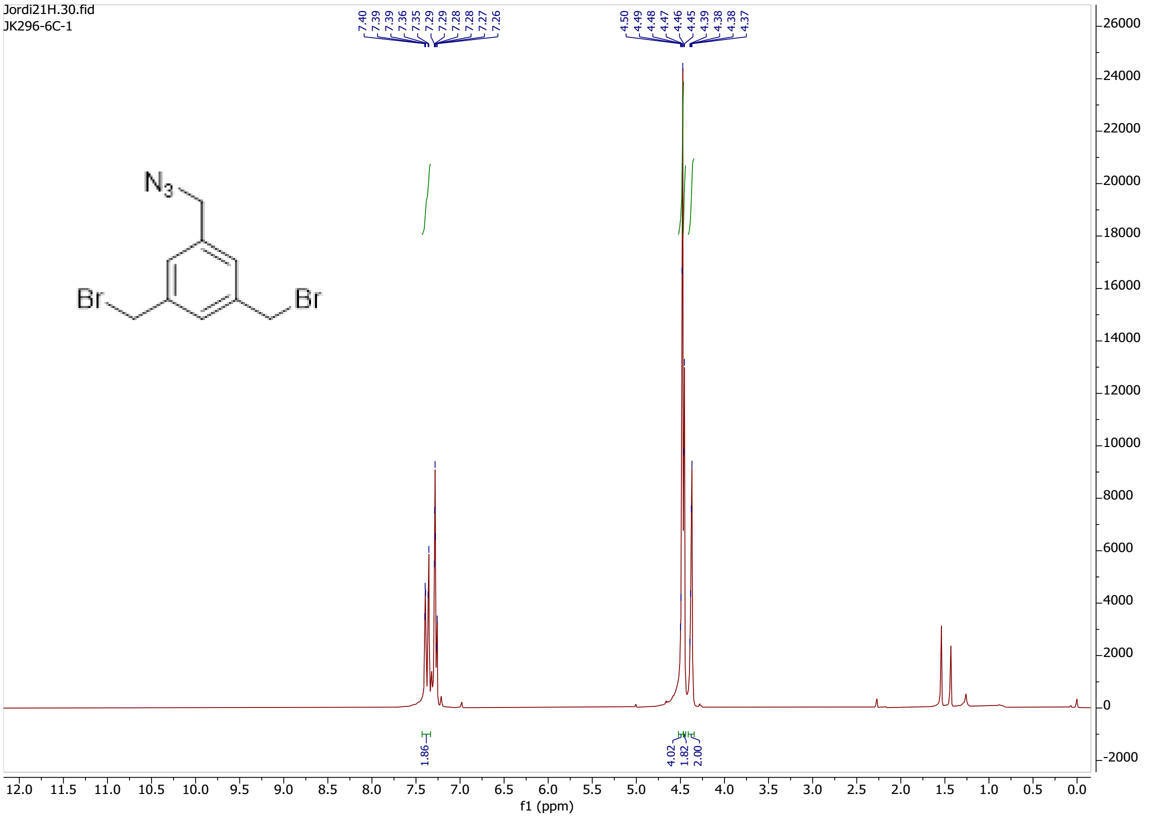

Figure S49. ¹H NMR spectrum of 1-(azidomethyl)-3,5-bis(bromomethyl)benzene.

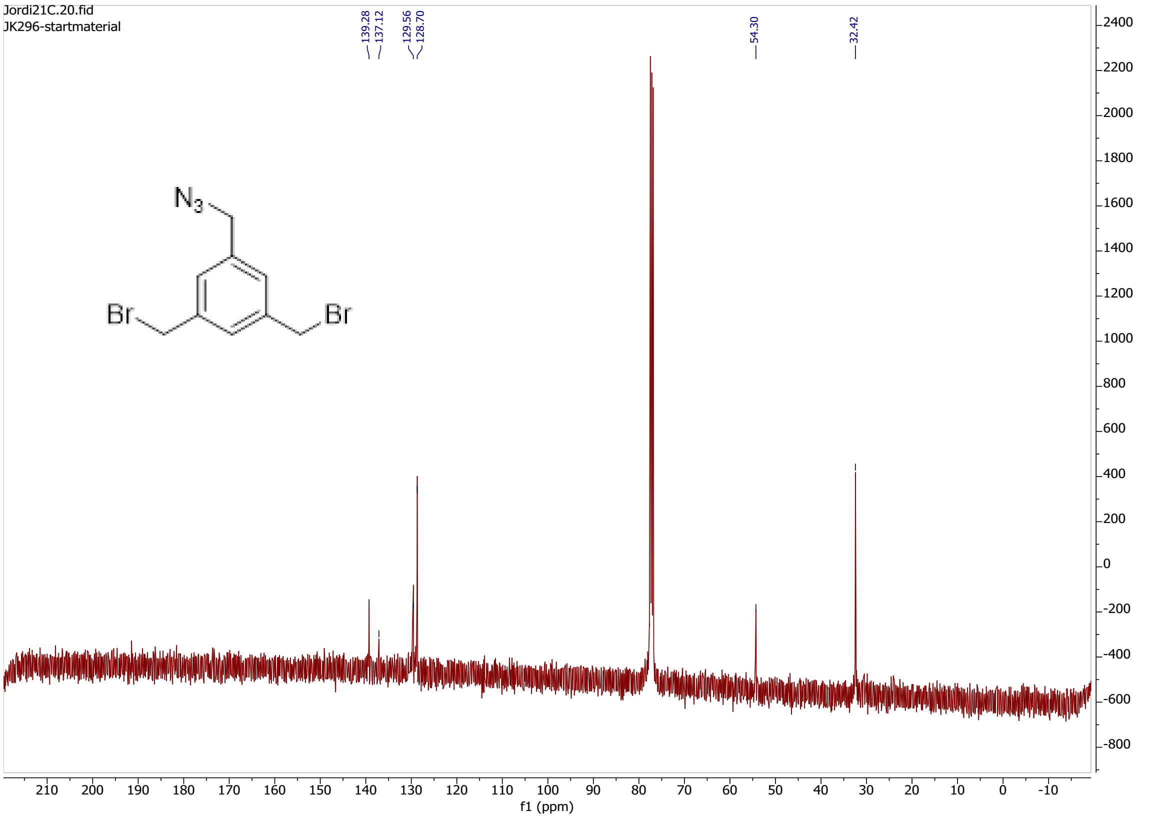

Figure S50. ¹³C NMR spectrum of 1-(azidomethyl)-3,5-bis(bromomethyl)benzene.

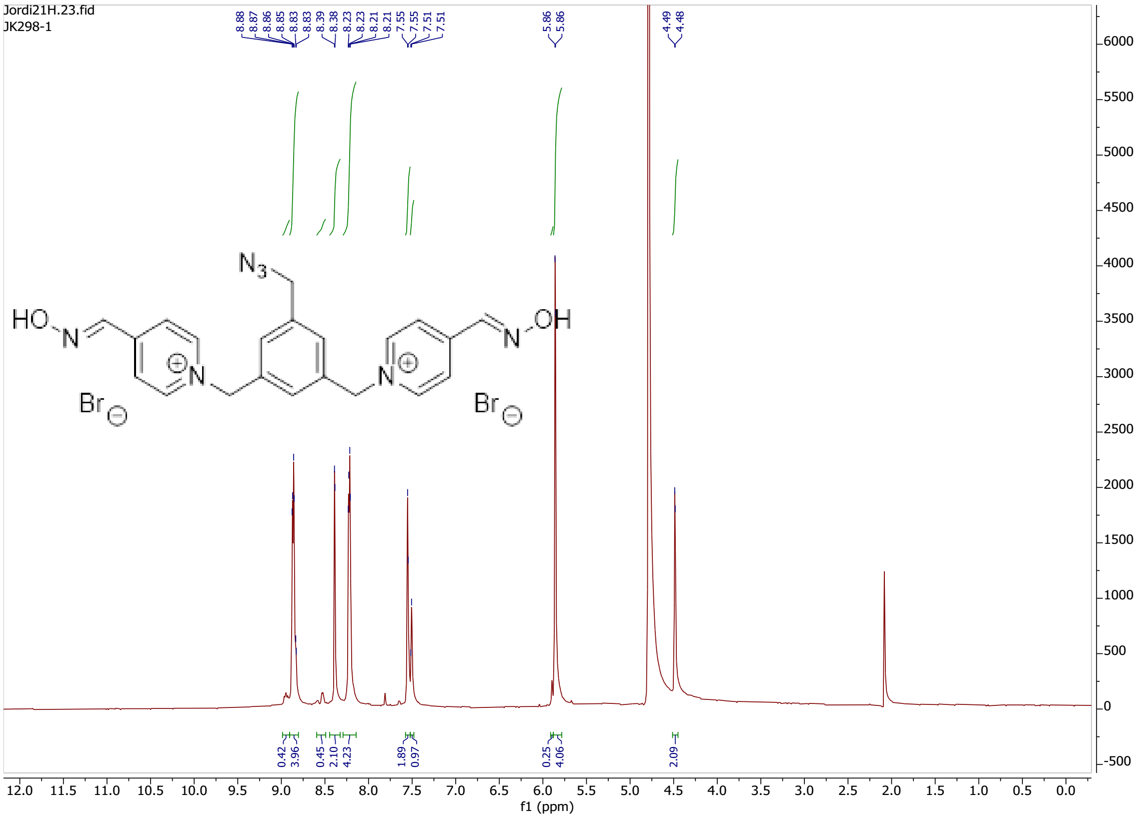

Figure S51. ^{1}H NMR spectrum of azido-diPyOx.

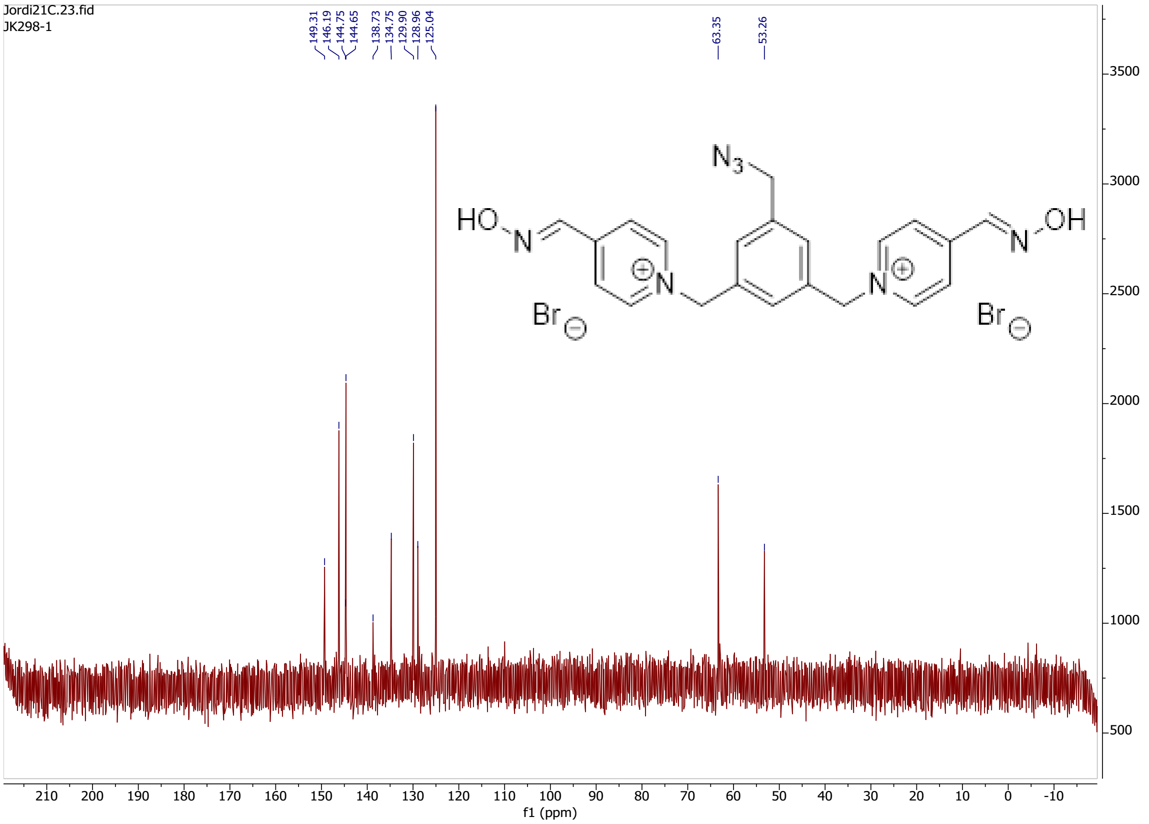

Figure S52. ^{13}C NMR spectrum of azido-diPyOx.

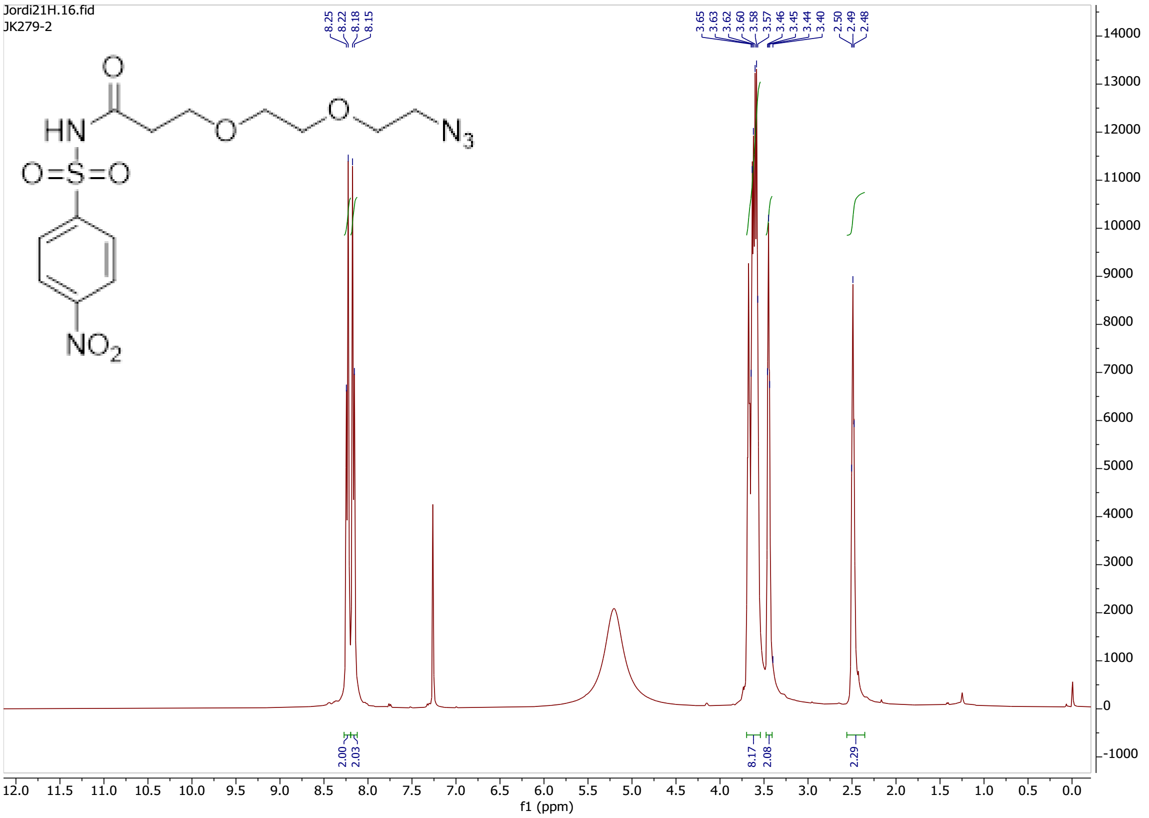

Figure S53. ^{1}H NMR spectrum of azido-ANANS precursor.

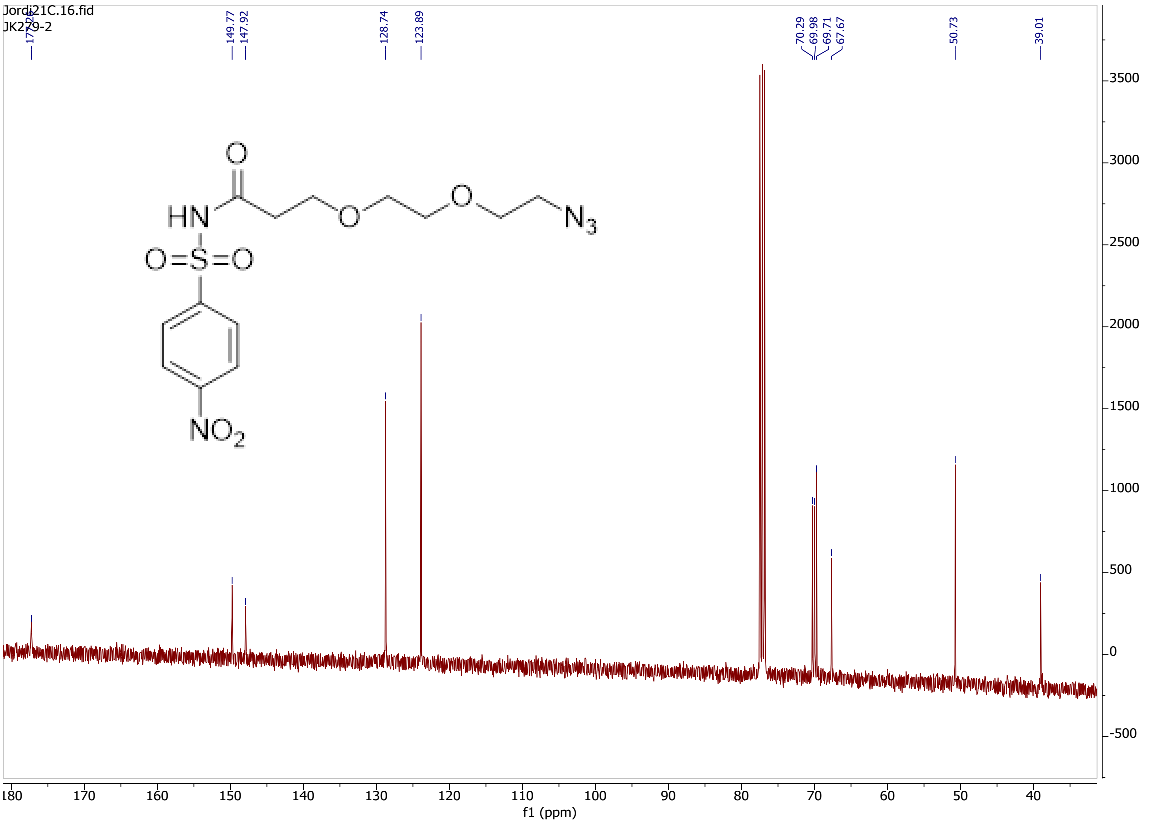

Figure S54. ^{13}C NMR spectrum of azido-ANANS precursor.

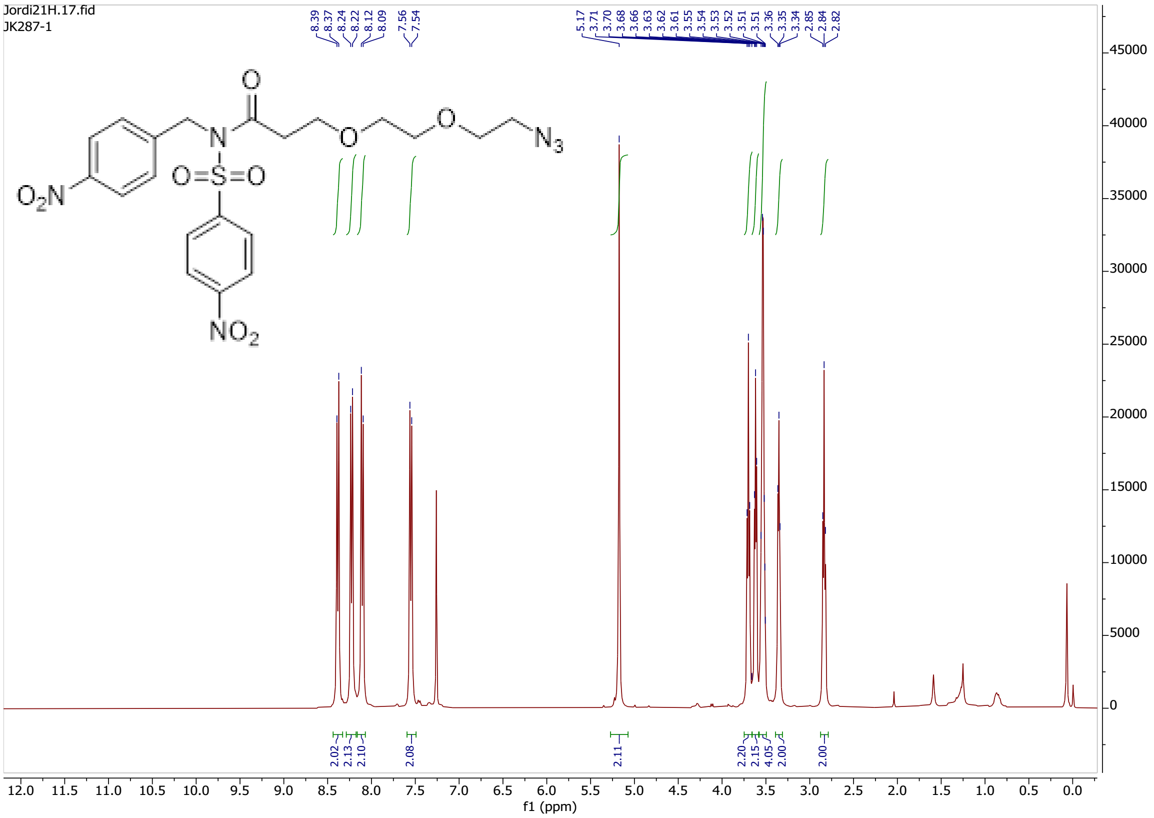

*Figure S55. ^{1}H NMR spectrum of azido-ANANS **2**.*

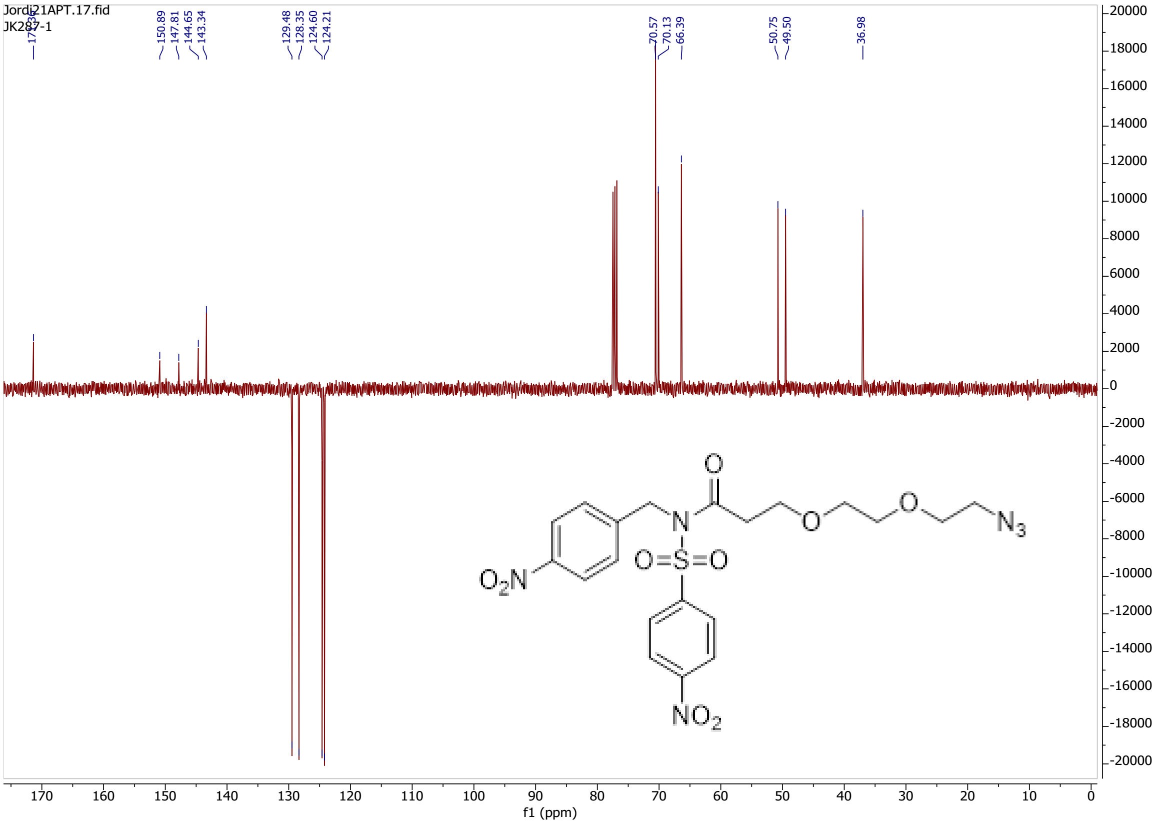

*Figure S56. ^{13}C APT NMR spectrum of azido-ANANS **2**.*

2.7 HPLC traces and purity of final compounds

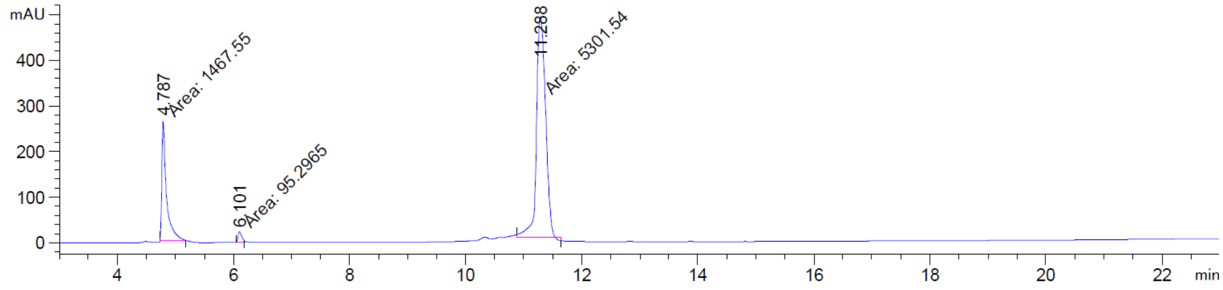

Figure S57. HPLC trace of azido-diDMAP (elution @ 4.8 and 11.3 min). Calculated purity = 99%.

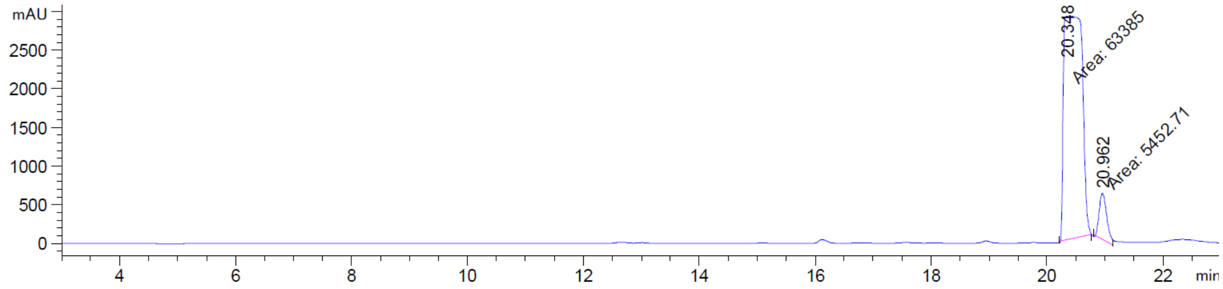

*Figure S58. HPLC trace of azido-thioester **1** (elution @ 20.3 and 21.0 min). Calculated purity = 99%.*

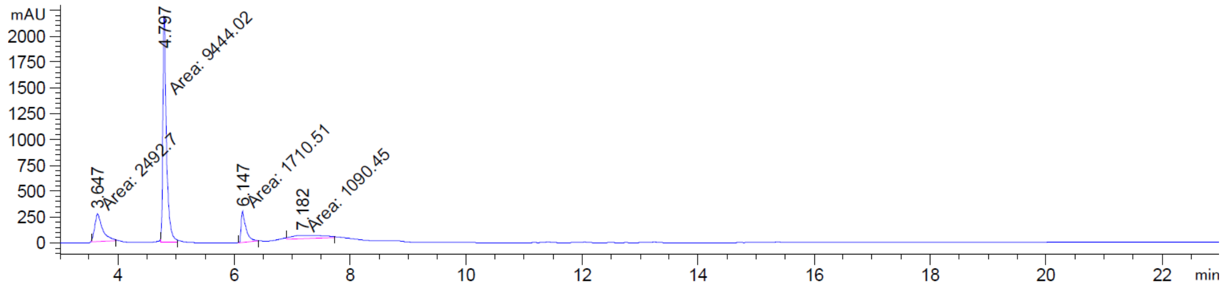

Figure S59. HPLC trace of azido-diPyOx (elution @ 3.6, 4.8 and 6.1 min). Calculated purity = 93%.

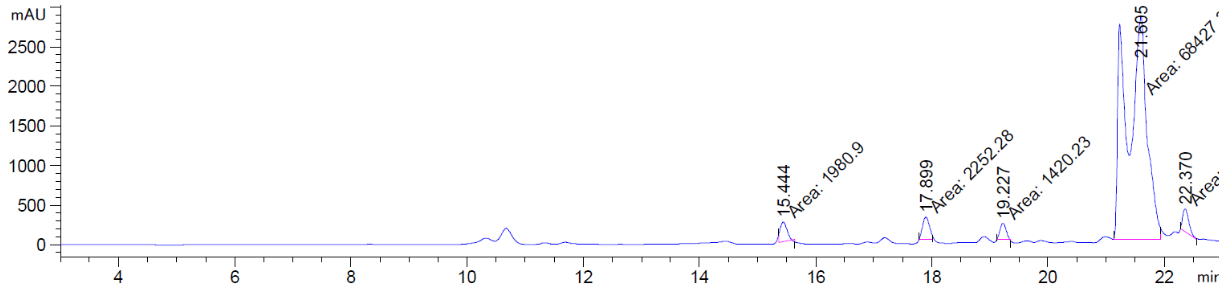

*Figure S60. HPLC trace of azido-ANANS **2** (elution @ 21.1 and 21.6 min). Calculated purity = 90%. Peak at ~10 min is contamination found which was also found in blanc runs. The other impurities at 15.4, 17.9, 19,2 and 22.4 were inert side-products that did not interfere with the conjugation reactions. Both peaks between 21 and 22 displayed the same HRMS mass, suggesting the presence of two conformational isomers.*
